# Supplementary material for: A Negative Energy Balance Is Associated with Metabolic Dysfunctions in the Hypothalamus of a Humanized Preclinical Model of Alzheimer’s Disease, the 5XFAD Mouse
Source: Int J Mol Sci. 2021 May 20;22(10):5365. doi: 10.3390/ijms22105365 (PMC8161294; doi:10.3390/ijms22105365)
Supplement: Supplementary file 1 [file ijms-22-05365-s001.zip › Figure S1.pdf]

Figure S1

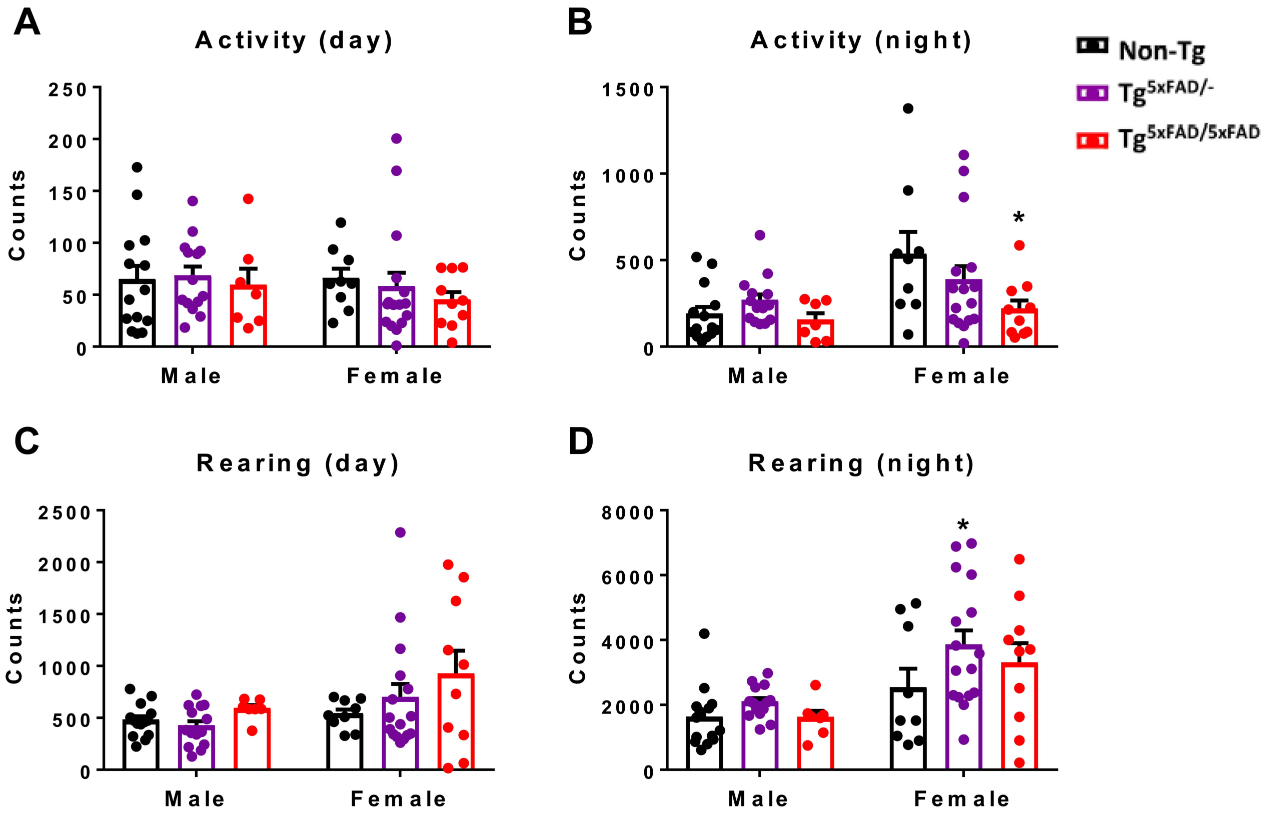

Figure S1: Activity (A,B) and rearing (C,D) measured as counts by infrared in 5xFAD mice placed in metabolic cages for 48 h, showing decreased activity in Tg5xFAD/5xFAD females during the night (n = 8–15 per group). Two-way ANOVA analysis with Tukey's post hoc test: * = $p < 0.05$ versus same-sex non-Tg group.
