# Supplementary material for: A Negative Energy Balance Is Associated with Metabolic Dysfunctions in the Hypothalamus of a Humanized Preclinical Model of Alzheimer’s Disease, the 5XFAD Mouse
Source: Int J Mol Sci. 2021 May 20;22(10):5365. doi: 10.3390/ijms22105365 (PMC8161294; doi:10.3390/ijms22105365)
Supplement: Supplementary file 1 [file ijms-22-05365-s001.zip › Figure S2.pdf]

Figure S2

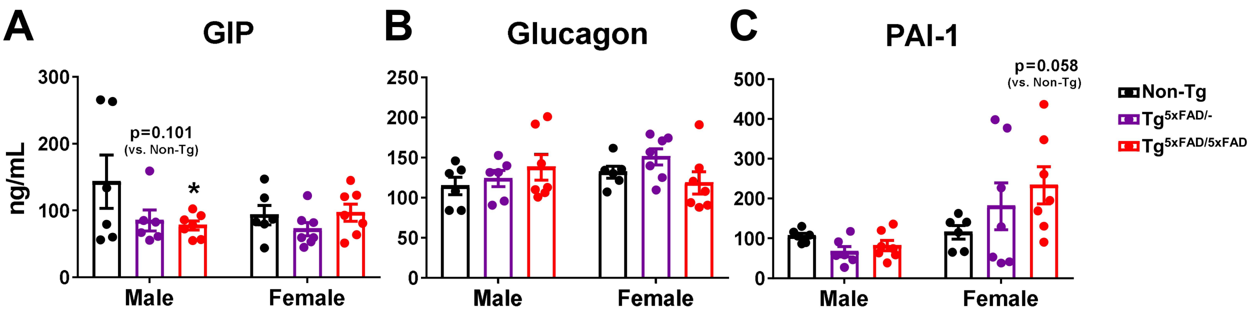

Figure S2: Plasma levels of grastric inhibitory polypeptide ((A), GIP), glucagon (B), and plas-minogen activator inhibitor-1 ((C), PAI-1) plasma levels showing a tendency to decreased GIP in males and increased PAI-1 in females (n = 6–7 per group).
