# Supplementary material for: A Negative Energy Balance Is Associated with Metabolic Dysfunctions in the Hypothalamus of a Humanized Preclinical Model of Alzheimer’s Disease, the 5XFAD Mouse
Source: Int J Mol Sci. 2021 May 20;22(10):5365. doi: 10.3390/ijms22105365 (PMC8161294; doi:10.3390/ijms22105365)
Supplement: Supplementary file 1 [file ijms-22-05365-s001.zip › Figure S3.pdf]

# Figure S3

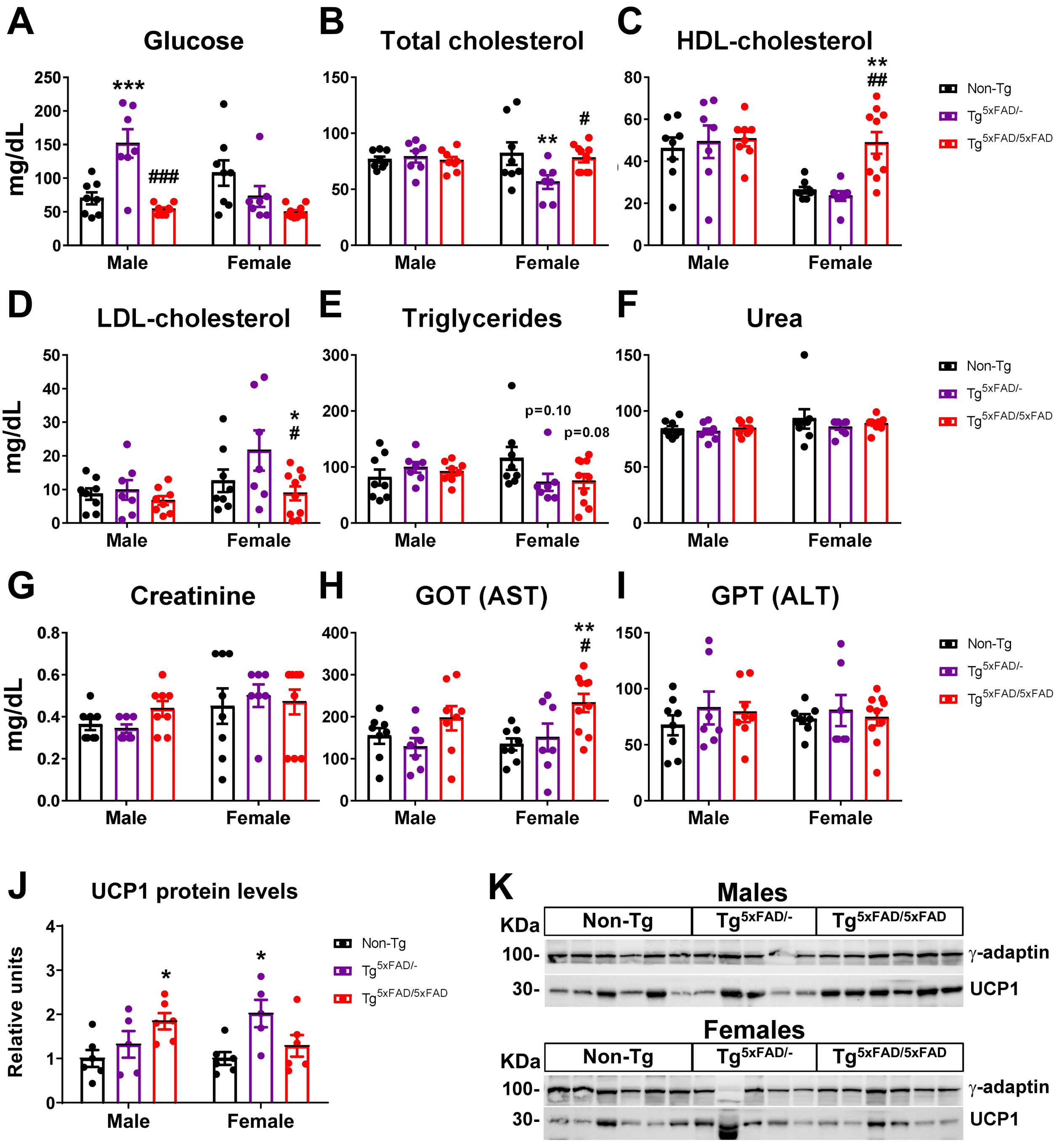


Figure S3: Plasma biochemical analysis of glucose (A), lipids (B-E), urea (F), creatinine (G), and hepatic enzyme (H,I) levels showing decreased trigyceride, total cholesterol, and LDL, and increased HDL and GOT in 5xFAD female mice. (J) Representation of Western blot membranes and (K) densitometric evaluation of UCP1 levels in white adipose tissue of 5xFAD mice at 6 months of age, showing increased UCP1 levels in transgenic 5xFAD male and female mice (n = 5–6 per group). Protein levels were normalized with γ-adaptin. Non-Tg males were set as 1 for protein relative units. Two-way ANOVA analysis with Tukey's post hoc test: * = $p < 0.05$, ** = $p < 0.01$, *** = $p < 0.001$ vs. same-sex non-Tg group; # = $p < 0.05$, ## = $p < 0.01$, ### = $p < 0.001$ versus same-sex Tg5xFAD/- group.
