# Supplementary material for: A Negative Energy Balance Is Associated with Metabolic Dysfunctions in the Hypothalamus of a Humanized Preclinical Model of Alzheimer’s Disease, the 5XFAD Mouse
Source: Int J Mol Sci. 2021 May 20;22(10):5365. doi: 10.3390/ijms22105365 (PMC8161294; doi:10.3390/ijms22105365)
Supplement: Supplementary file 1 [file ijms-22-05365-s001.zip › Figure S4.pdf]

Figure S4

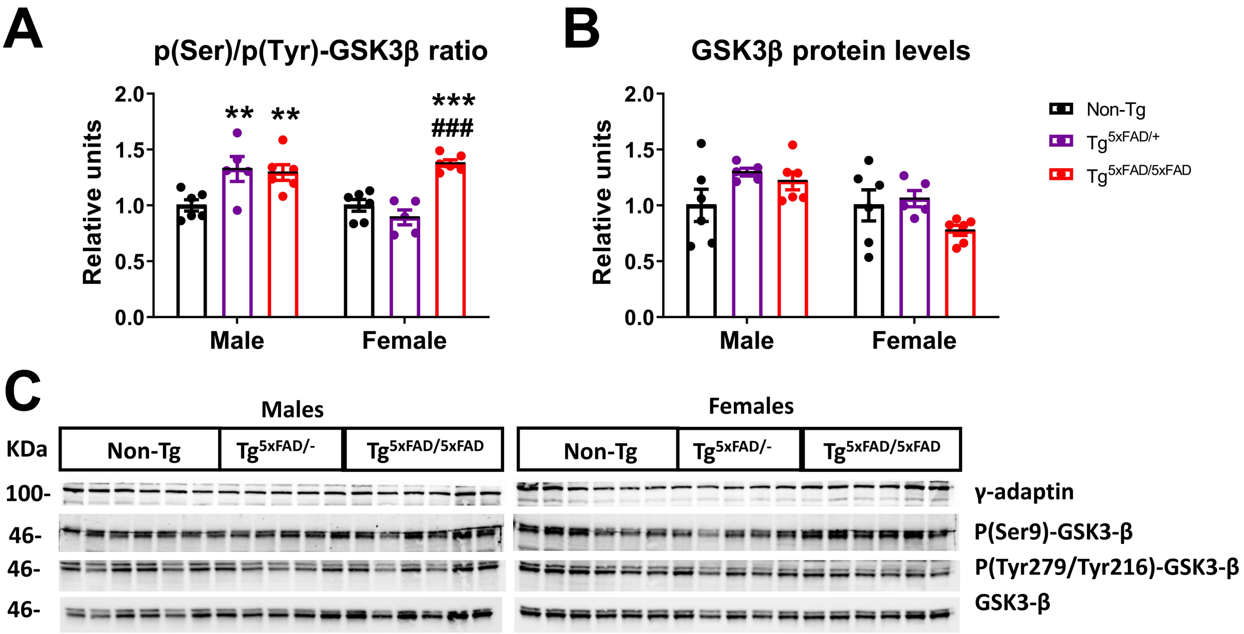

Figure S4: Densitometric evaluation of the ratio of serine (inhibitory) and tyrosine (activating) phosphorylation of GSK3-β (A) and GSK3-β protein levels (B) in the hypothalamus of 5xFAD mice at 6 months of age, showing increased Ser/Tyr ratio in transgenic 5xFAD male and female mice (n = 5–6 per group). Protein levels were normalized with γ-adaptin. Non-Tg males were set as 1 for protein relative units. Two-way ANOVA analysis with Tukey's post hoc test: * = $p < 0.05$, ** = $p < 0.01$, *** = $p < 0.001$ vs. same-sex non-Tg group; # = $p < 0.05$, ## = $p < 0.01$, ### = $p < 0.001$ versus same-sex Tg5xFAD/- group
