# Supplementary material for: A Negative Energy Balance Is Associated with Metabolic Dysfunctions in the Hypothalamus of a Humanized Preclinical Model of Alzheimer’s Disease, the 5XFAD Mouse
Source: Int J Mol Sci. 2021 May 20;22(10):5365. doi: 10.3390/ijms22105365 (PMC8161294; doi:10.3390/ijms22105365)

Figure S5. Unedited blots from Figure 2

LRP1 males

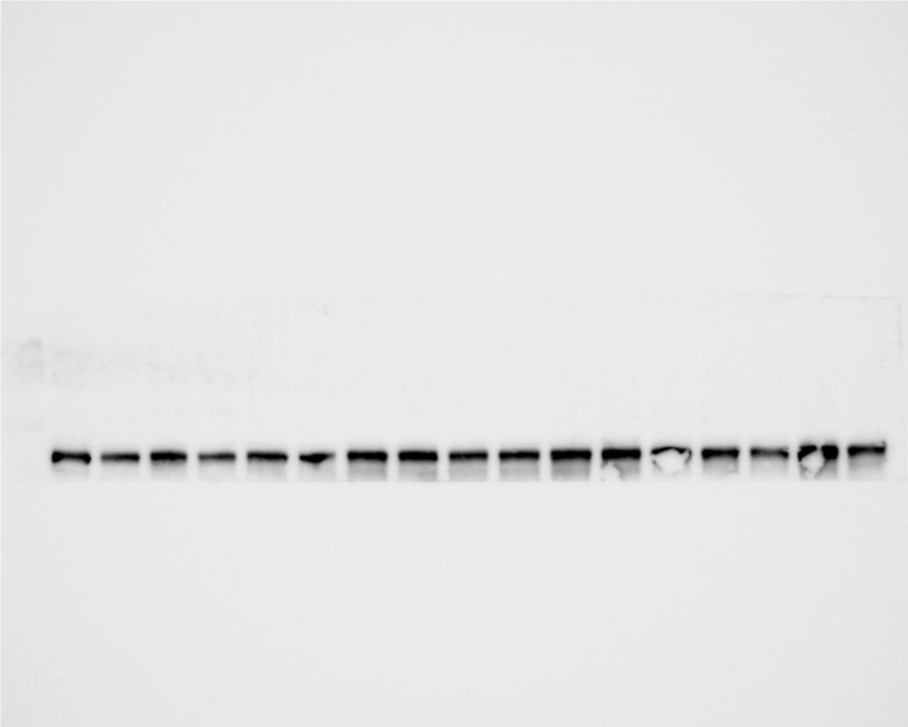

LRP1 females

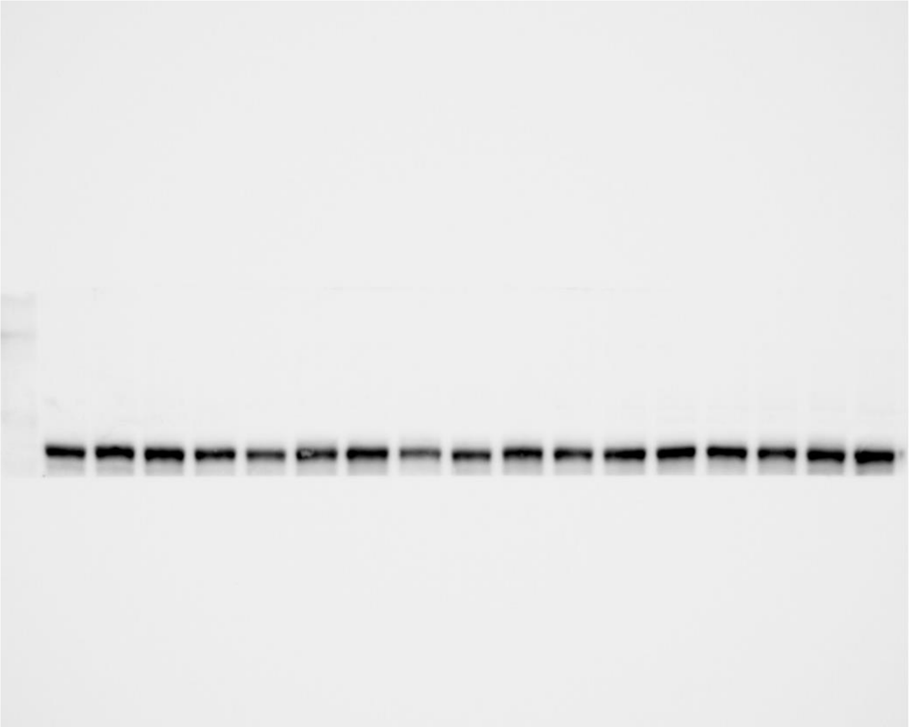

AT8-Tau males

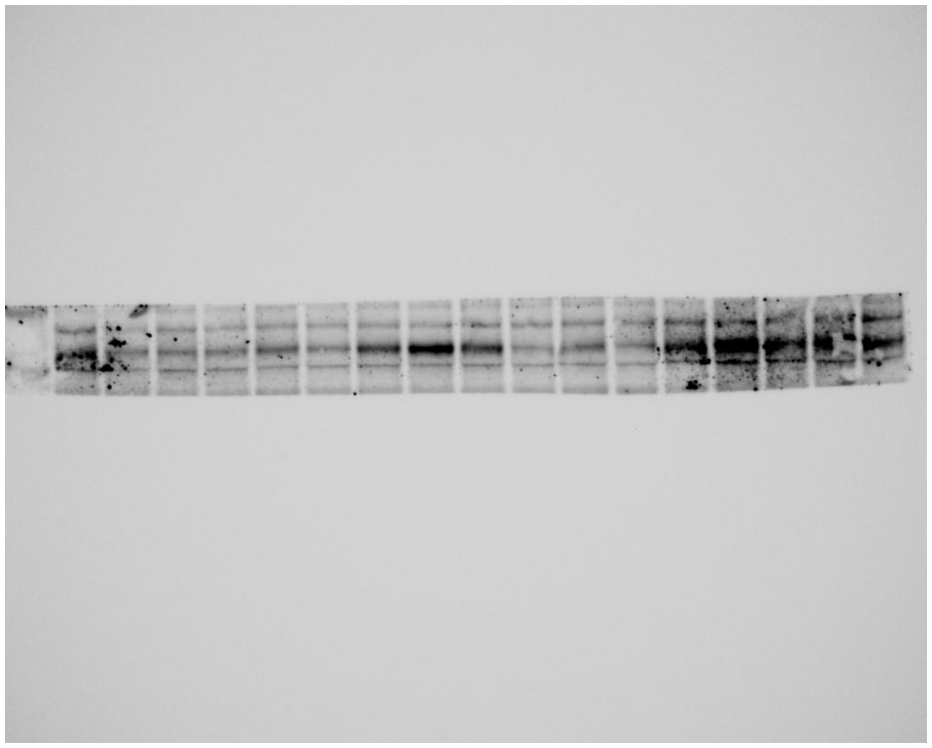

AT8-Tau females

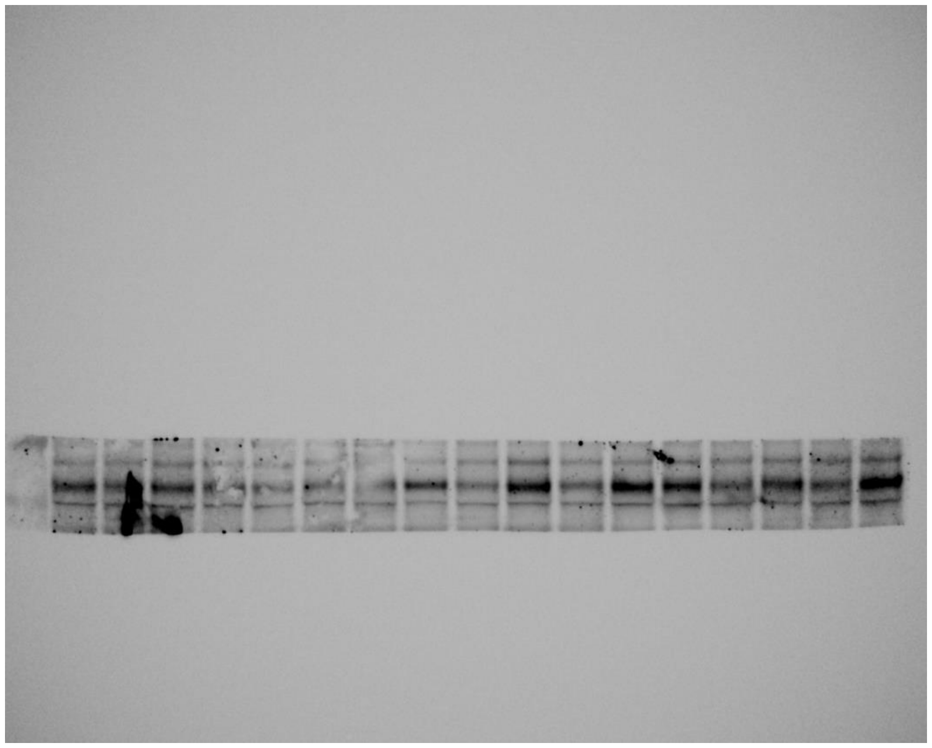

Figure S5. Unedited blots from Figure 2

Tau males

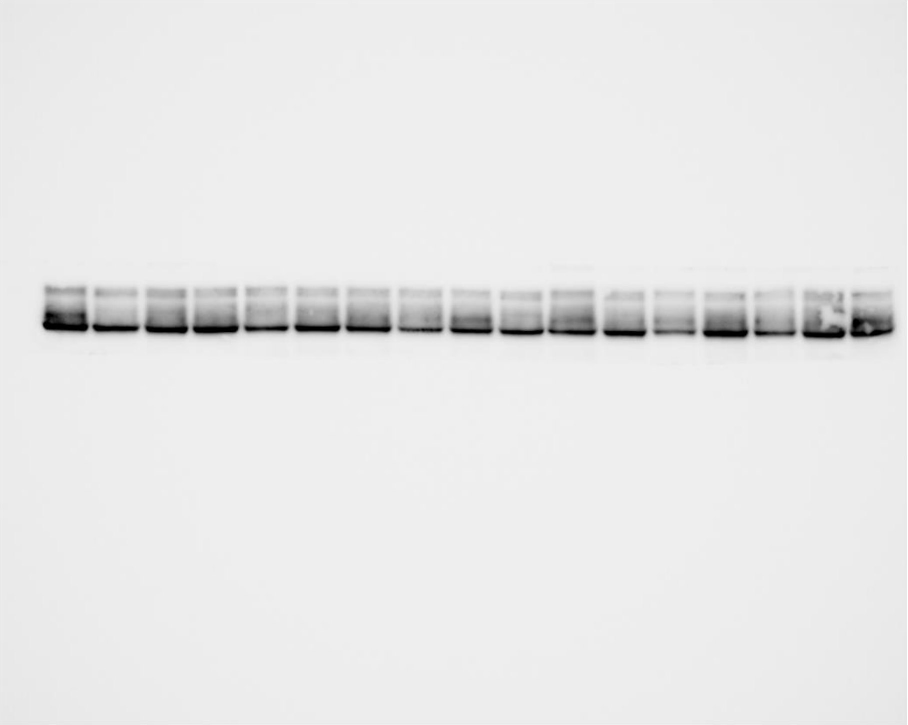

Tau females

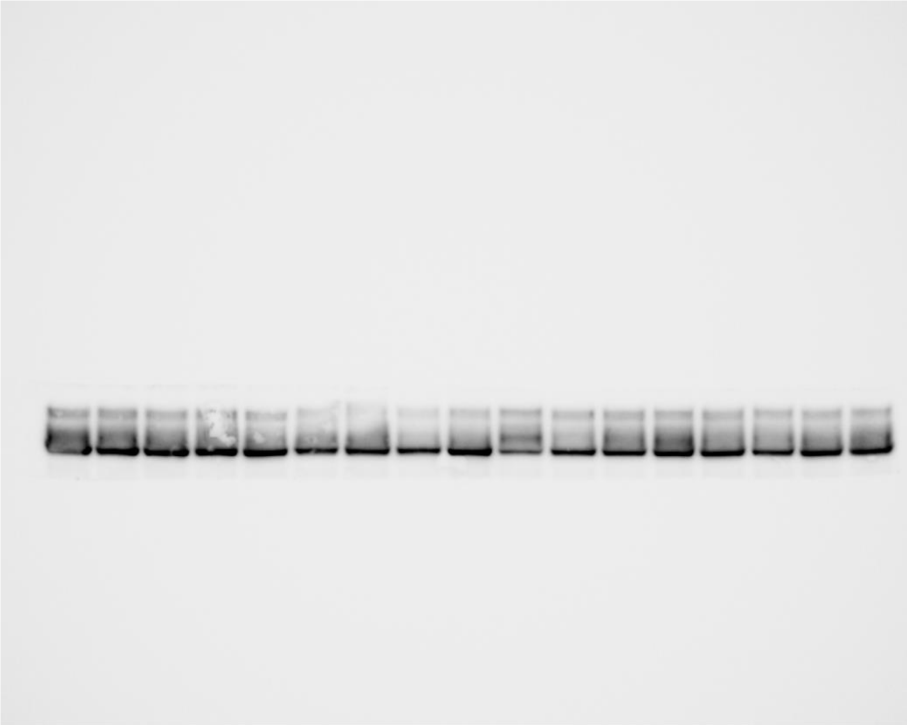

p35 males

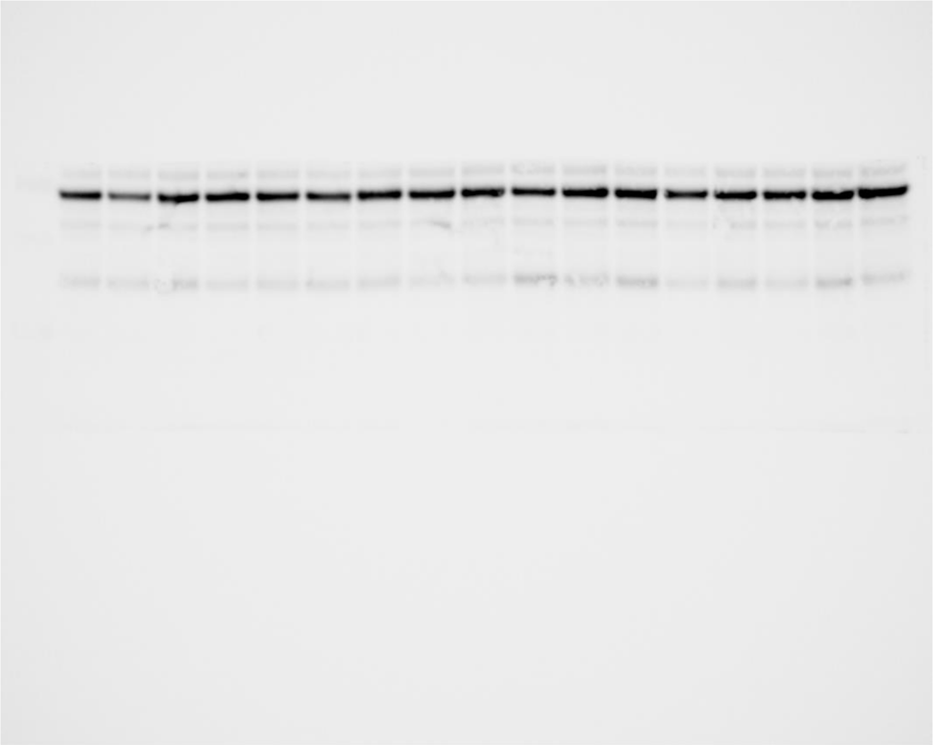

p35 females

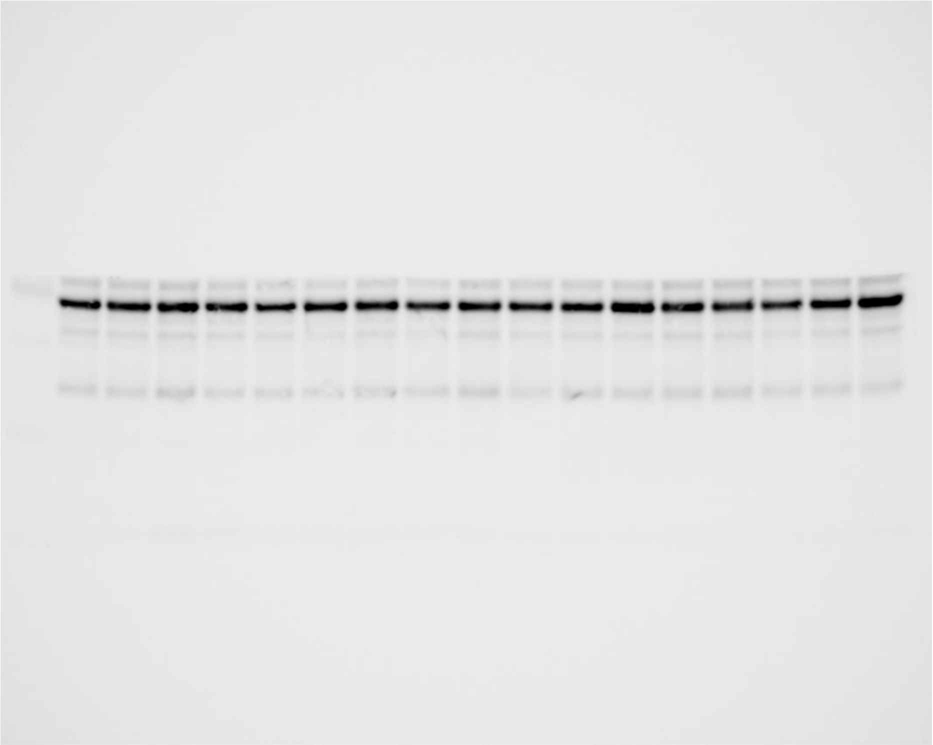

Figure S5. Unedited blots from Figure 2

CDK5 males

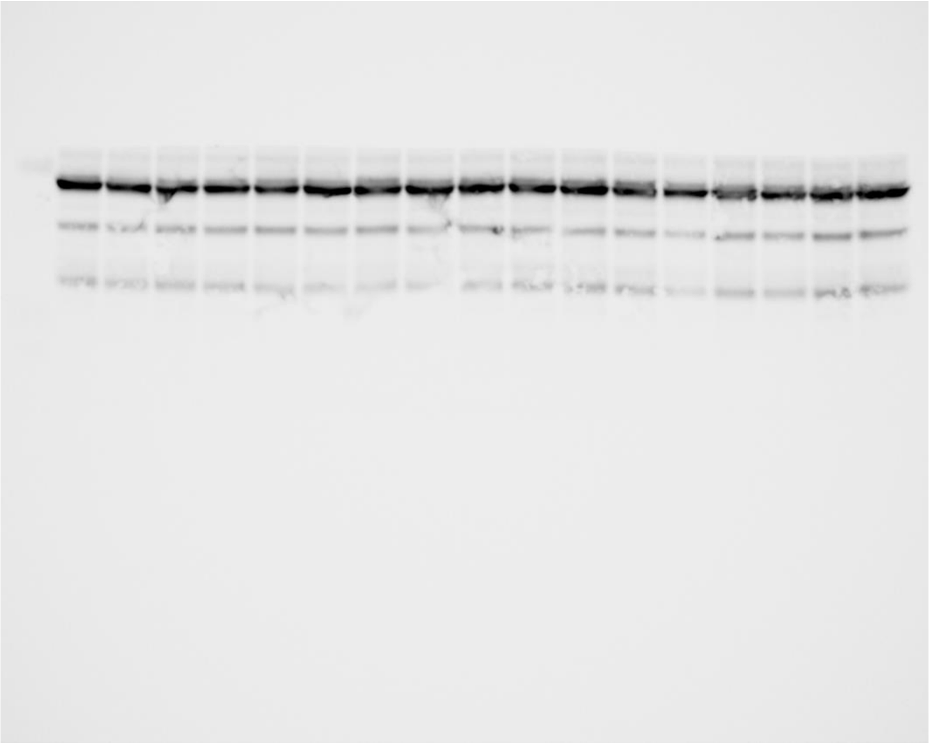

CDK5 females

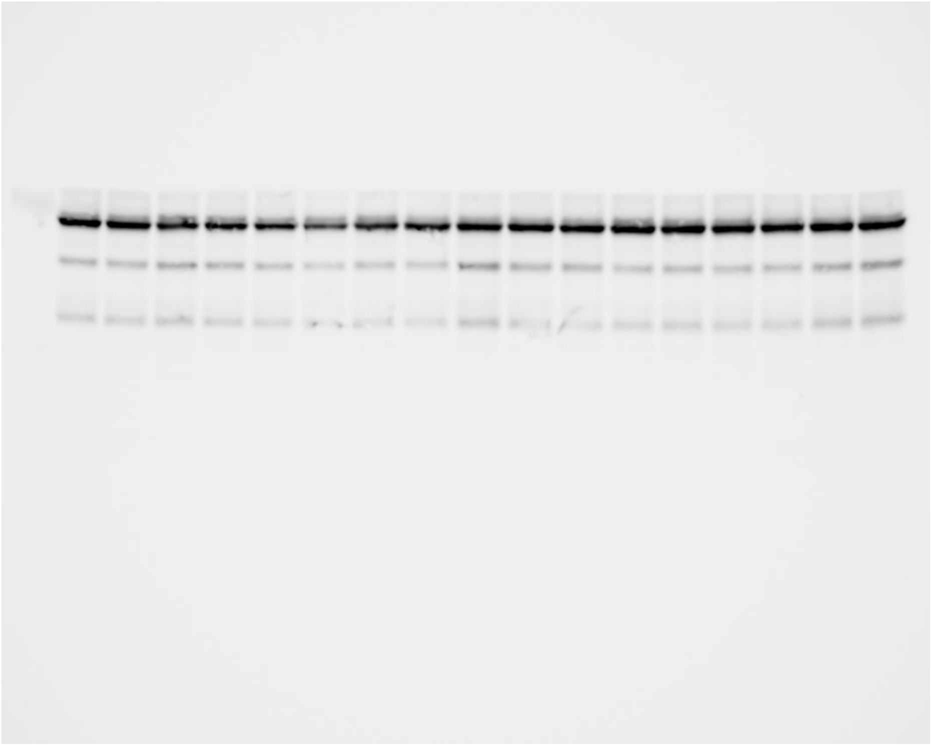

p25 males

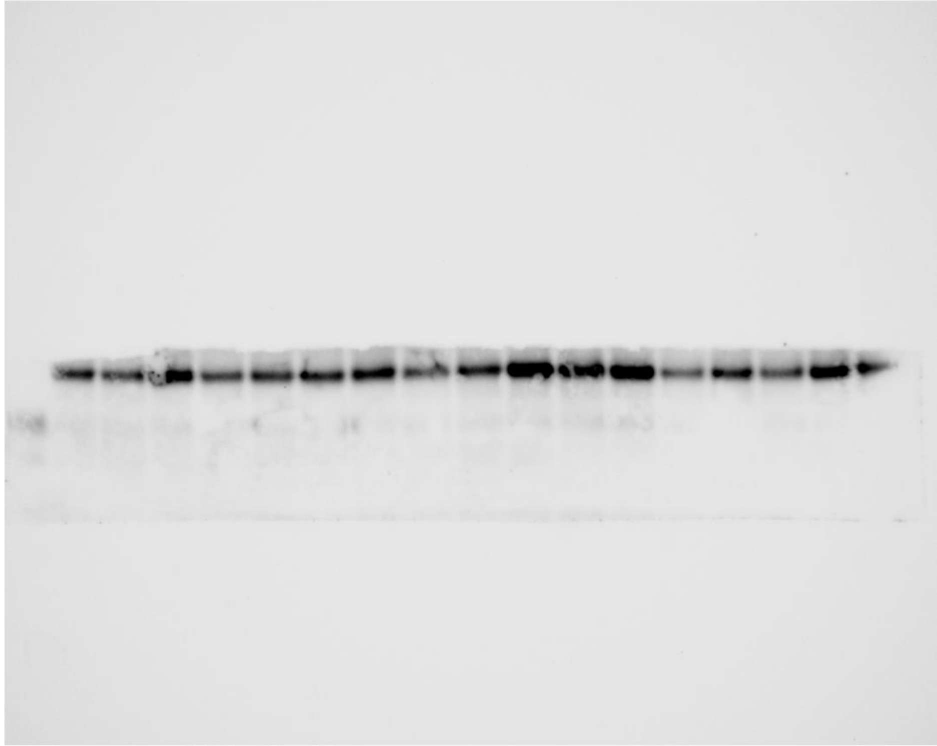

p25 females

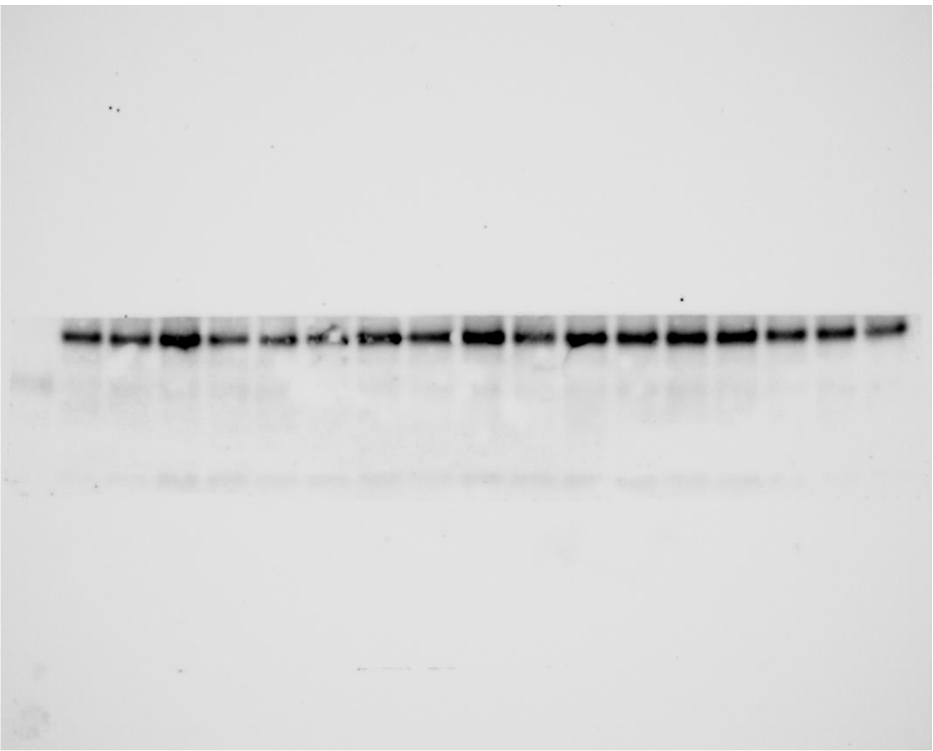

Figure S5. Unedited blots from Figure 2

γ-adaptin males

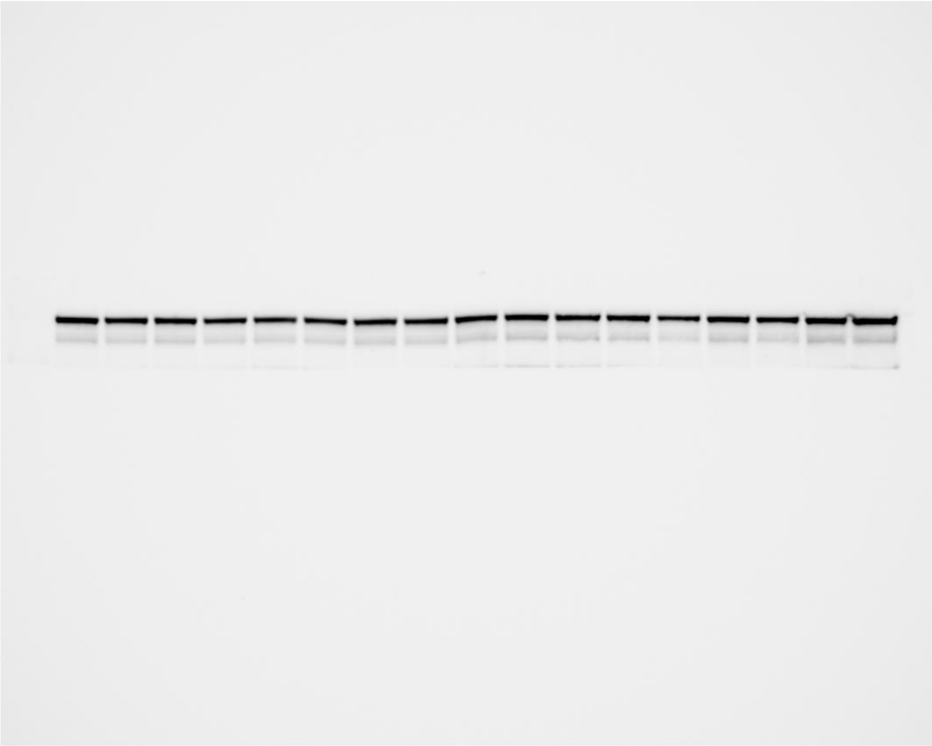

γ-adaptin females

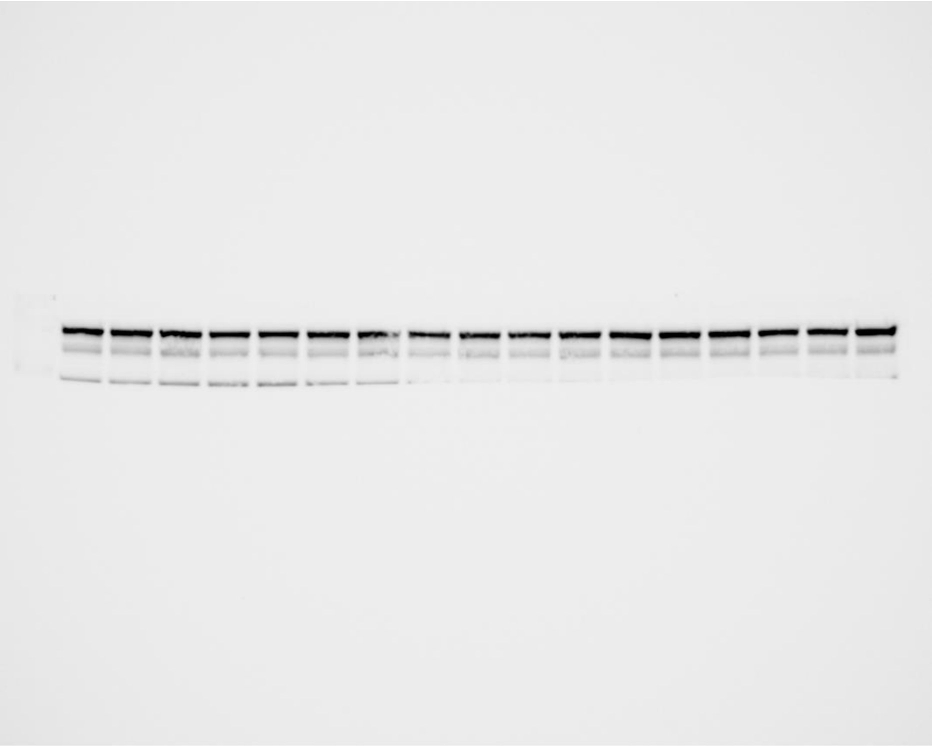

Ponceau staining males

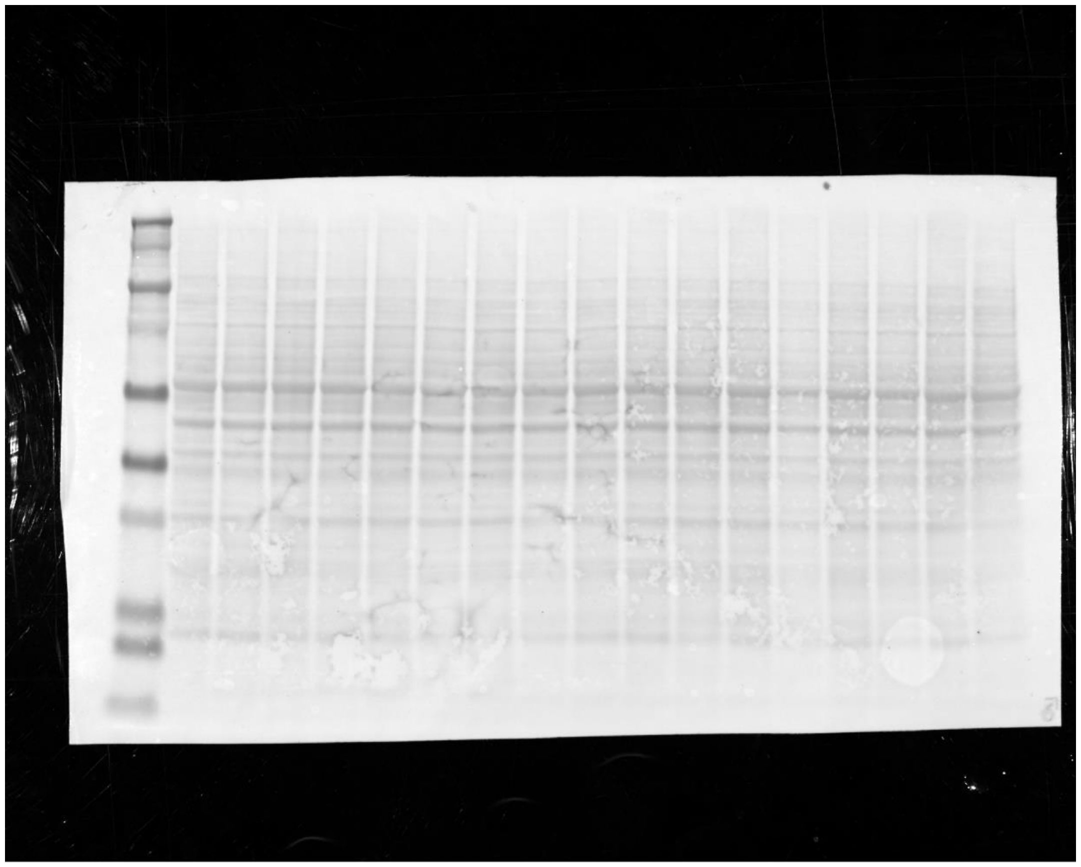

Ponceau staining females

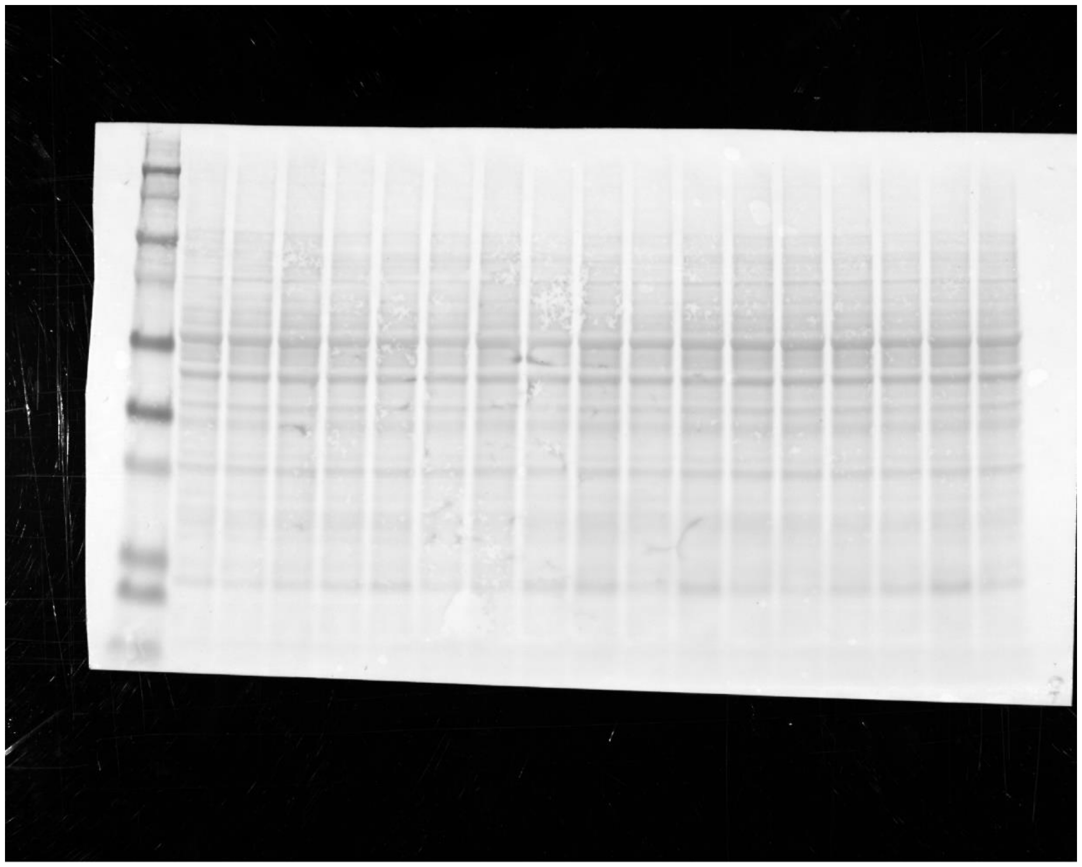

Figure S5. Unedited blots from Figure 3

GFAP males

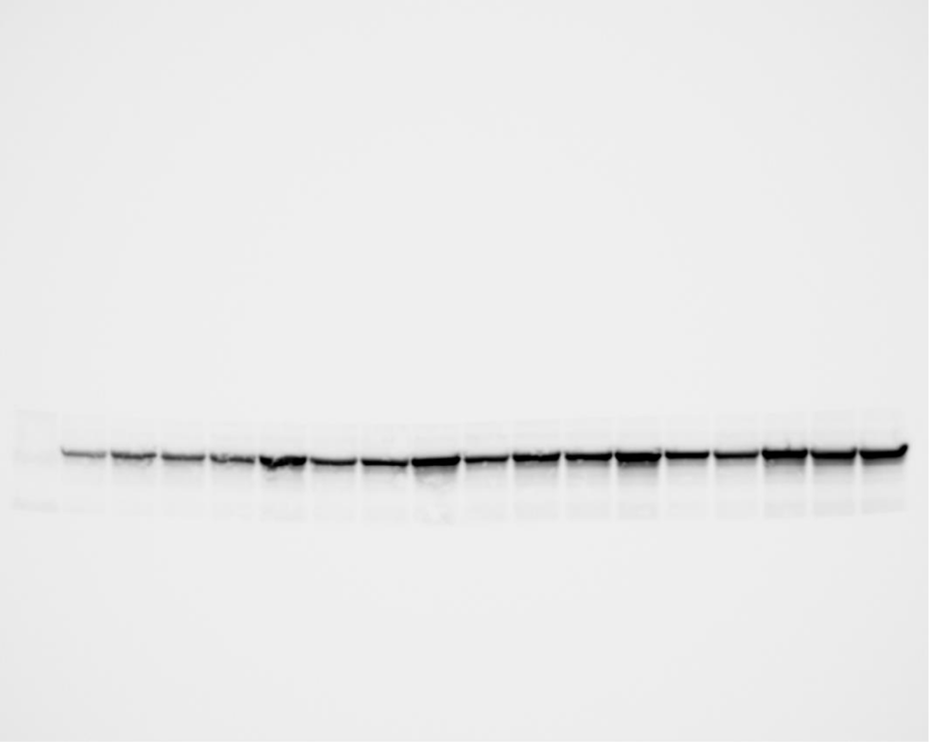

GFAP females

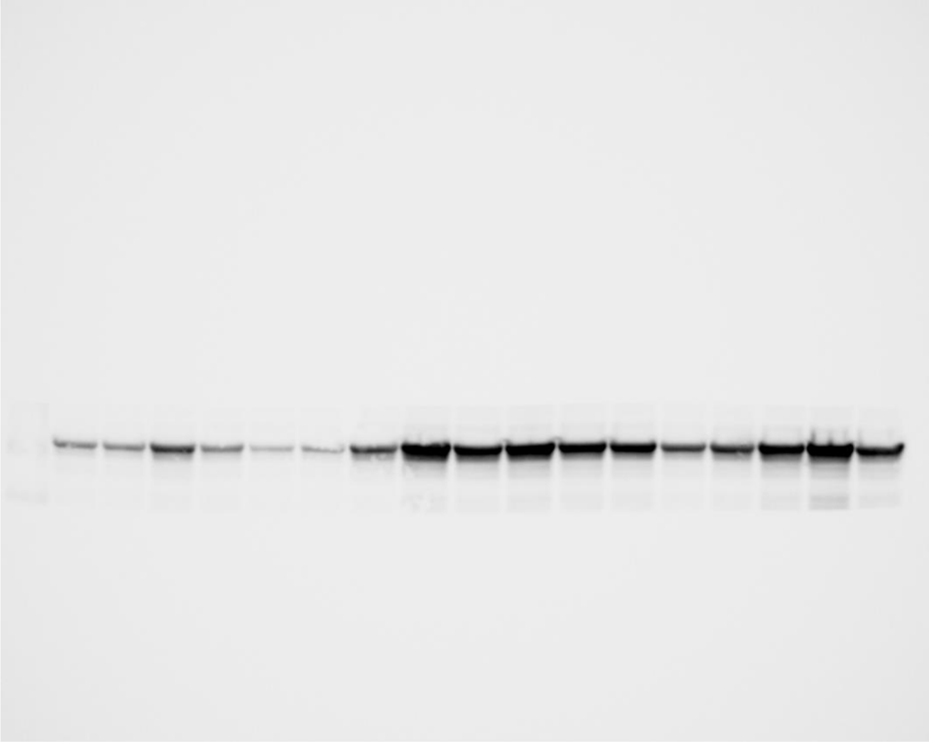

IL-1β males

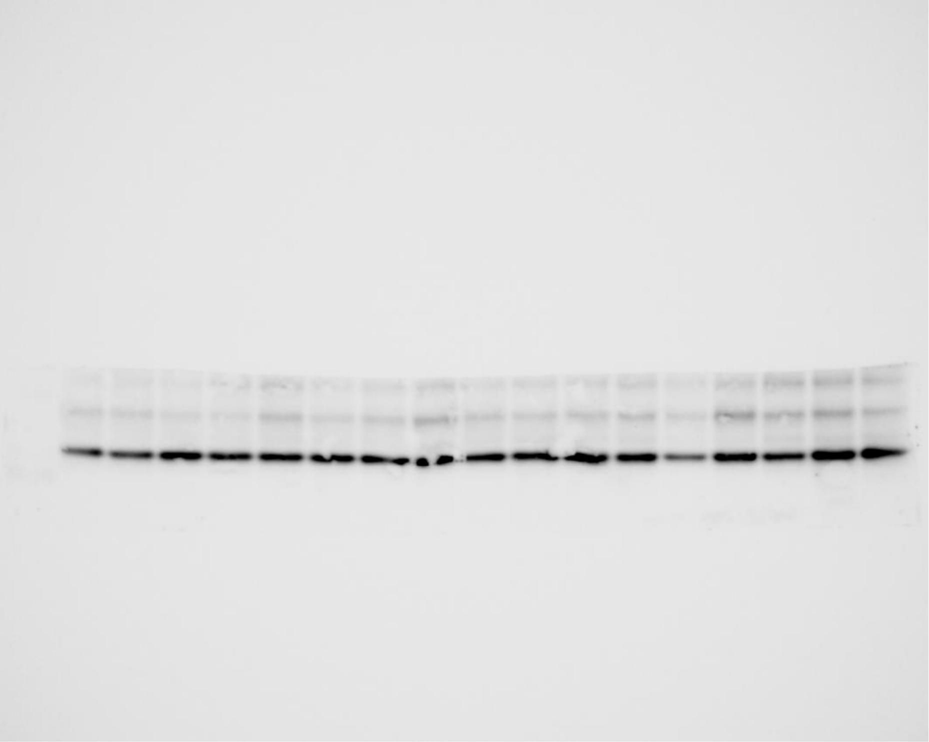

IL-1β females

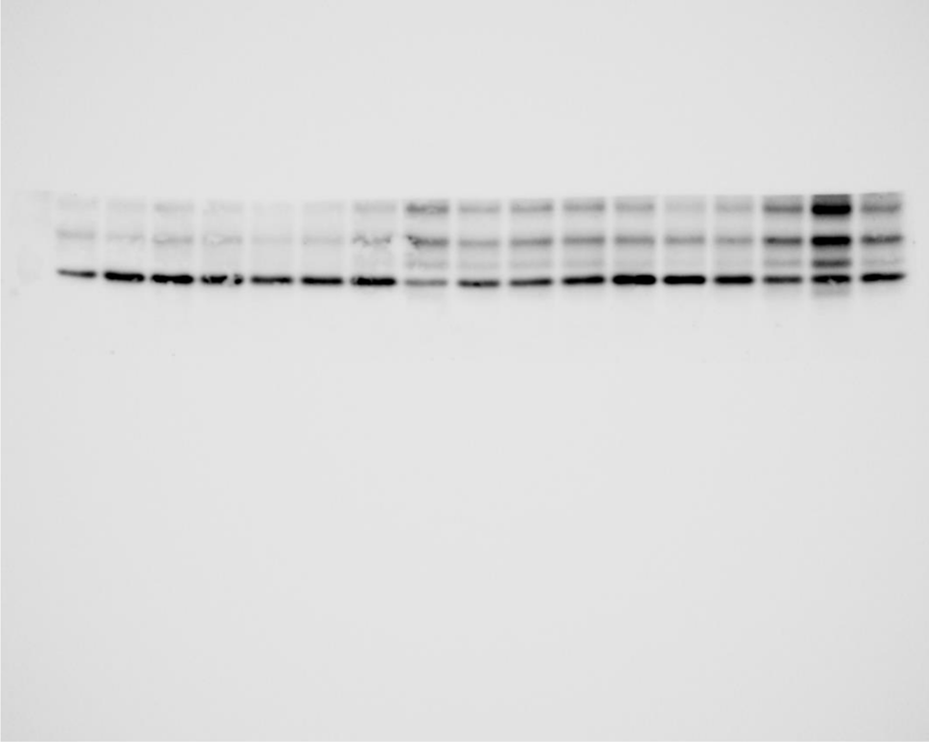

Figure S5. Unedited blots from Figure 3

IBA1 males

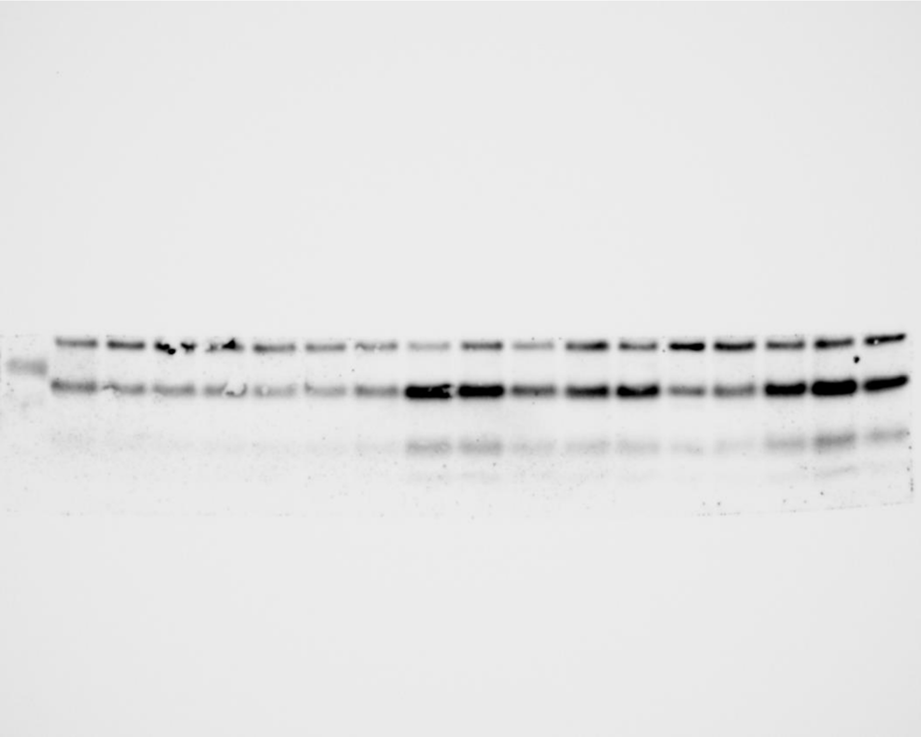

IBA1 females

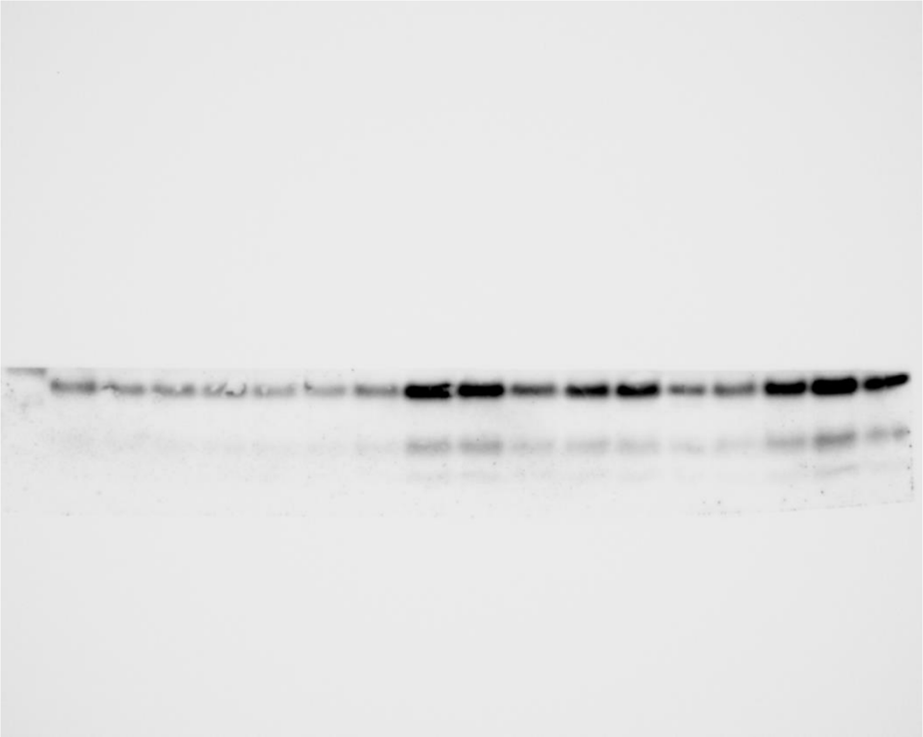

γ-adaptin males

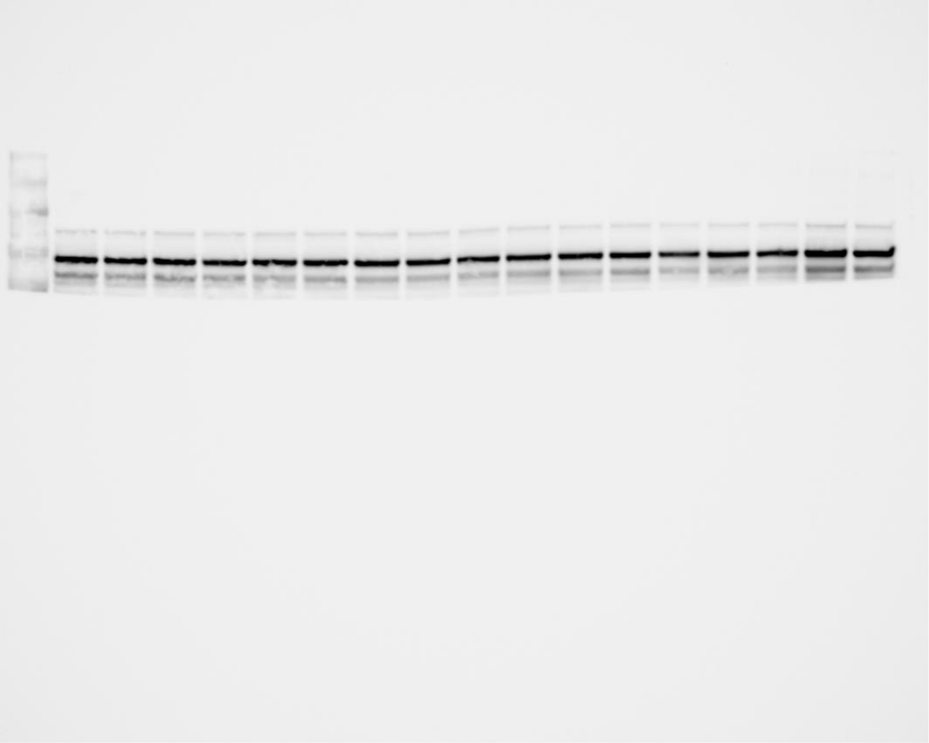

γ-adaptin females

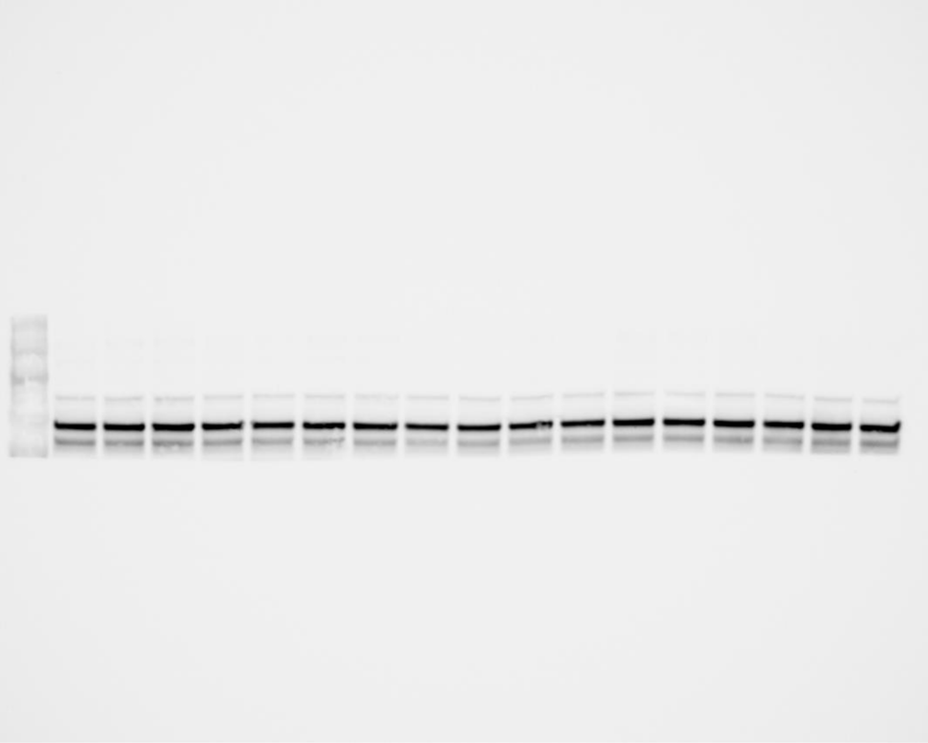

Figure S5. Unedited blots from Figure 3

Ponceau staining males

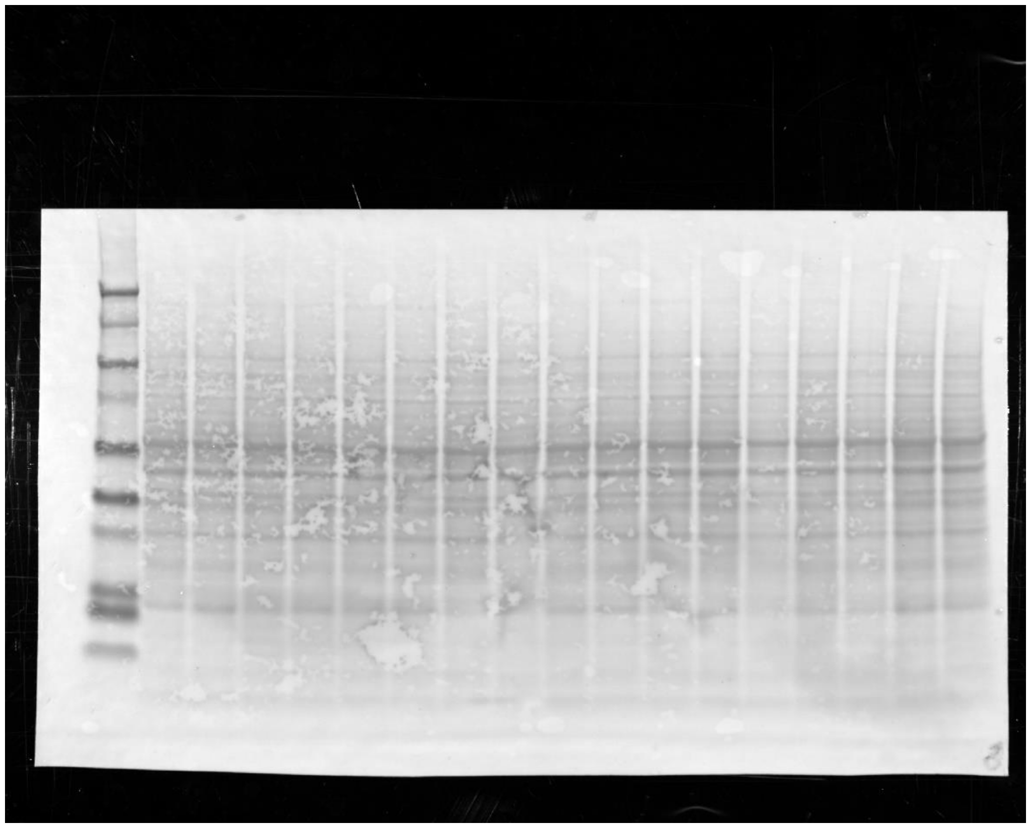

Ponceau staining females

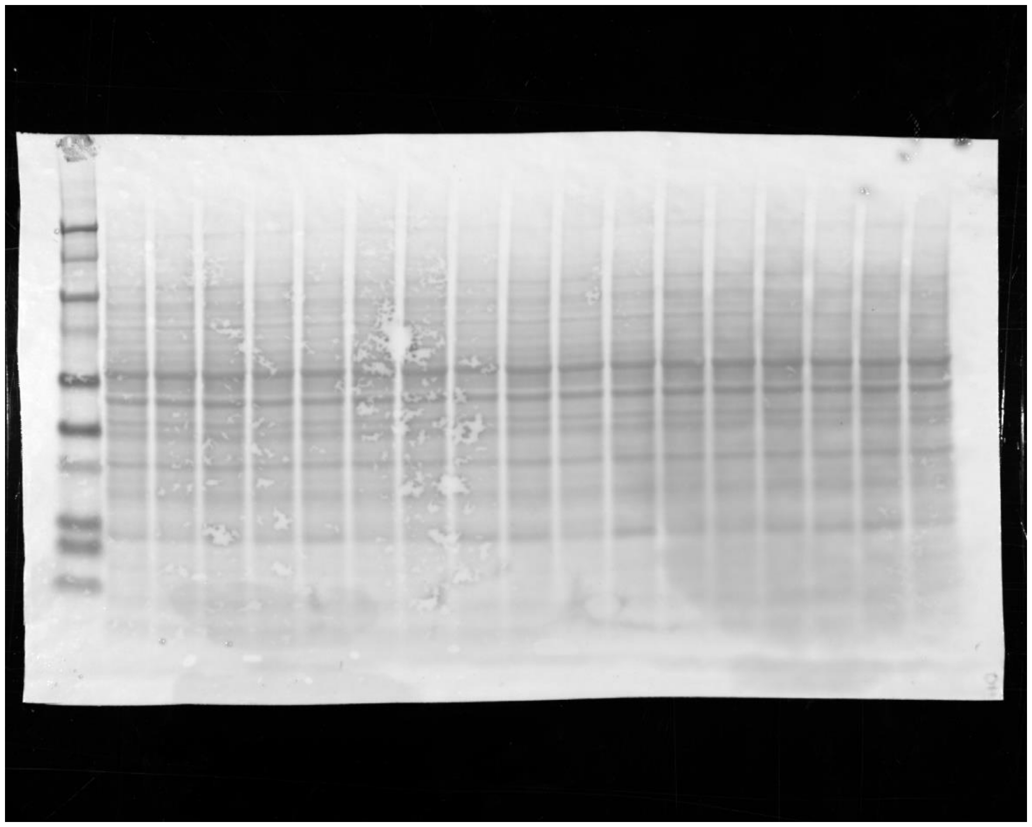

Figure S5. Unedited blots from Figure 4

p-IR males

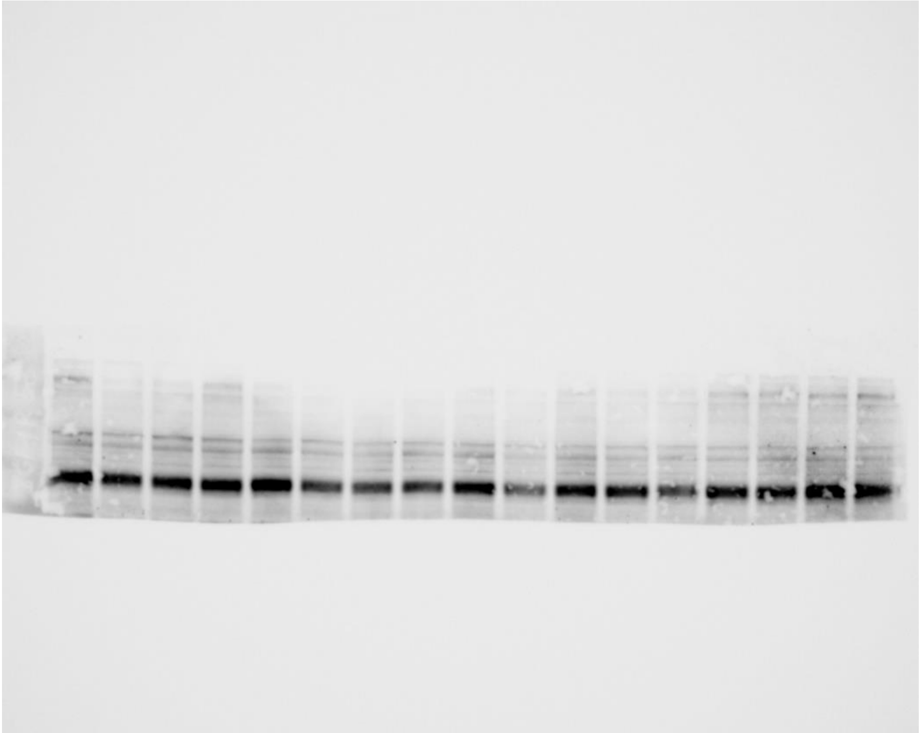

p-IR females

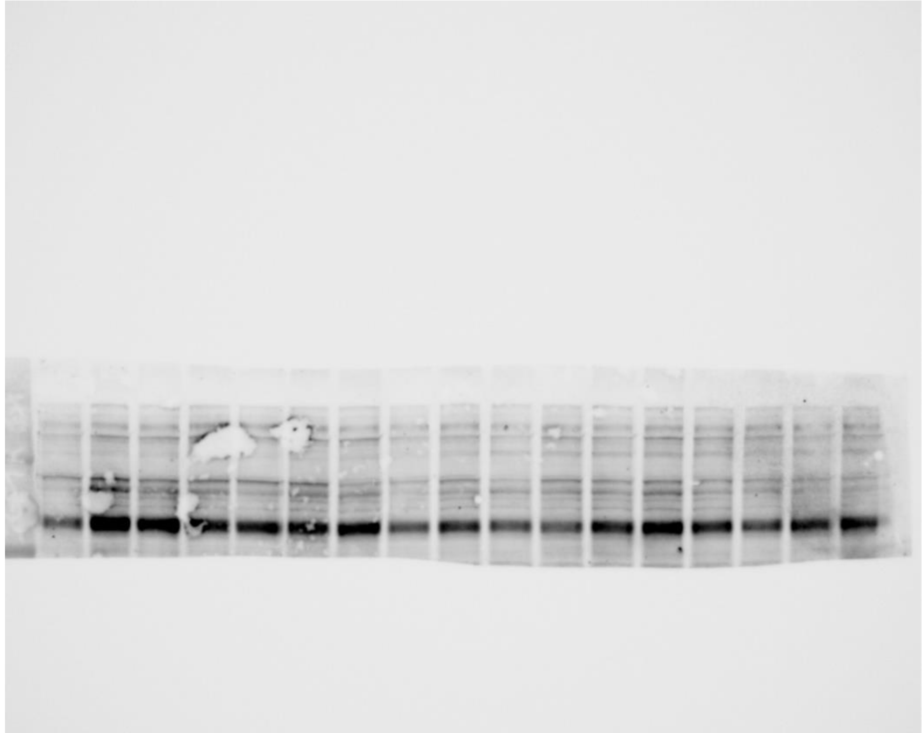

IR males

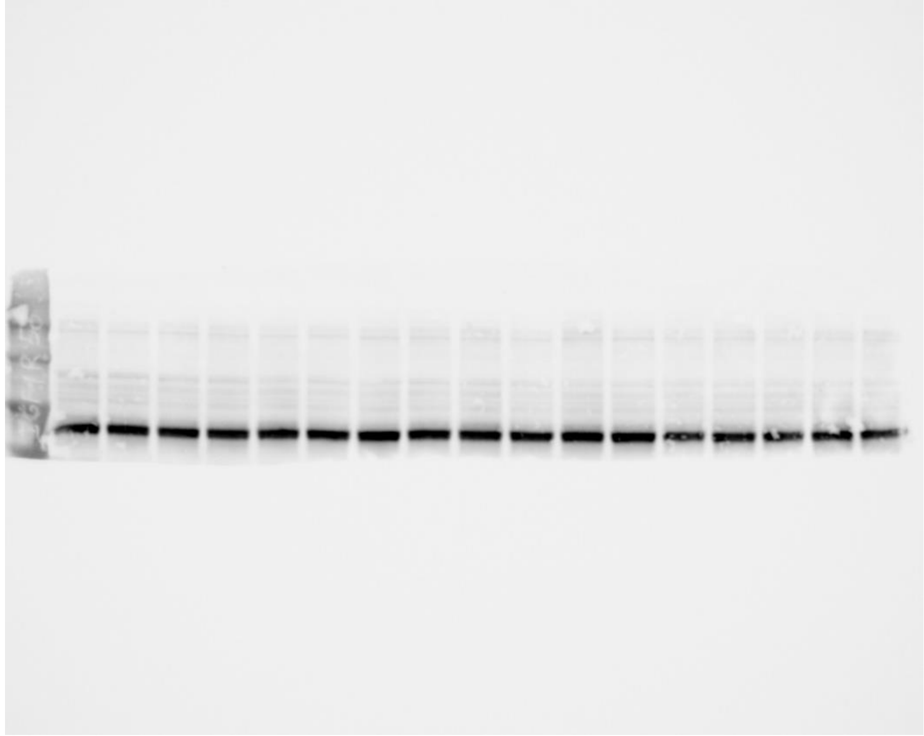

IR females

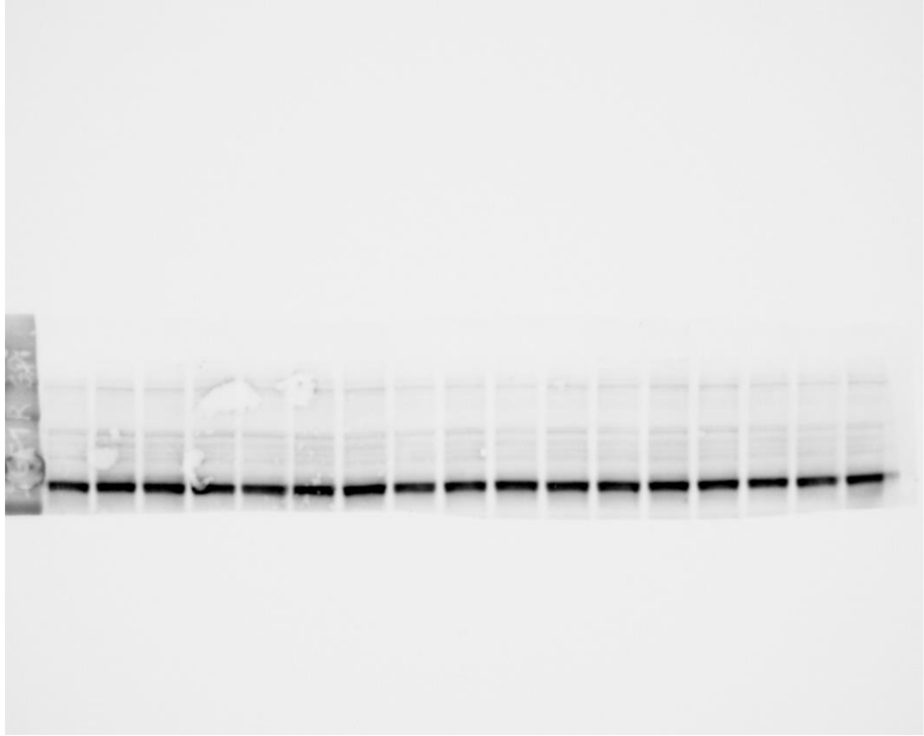

Figure S5. Unedited blots from Figure 4

GLP1R males

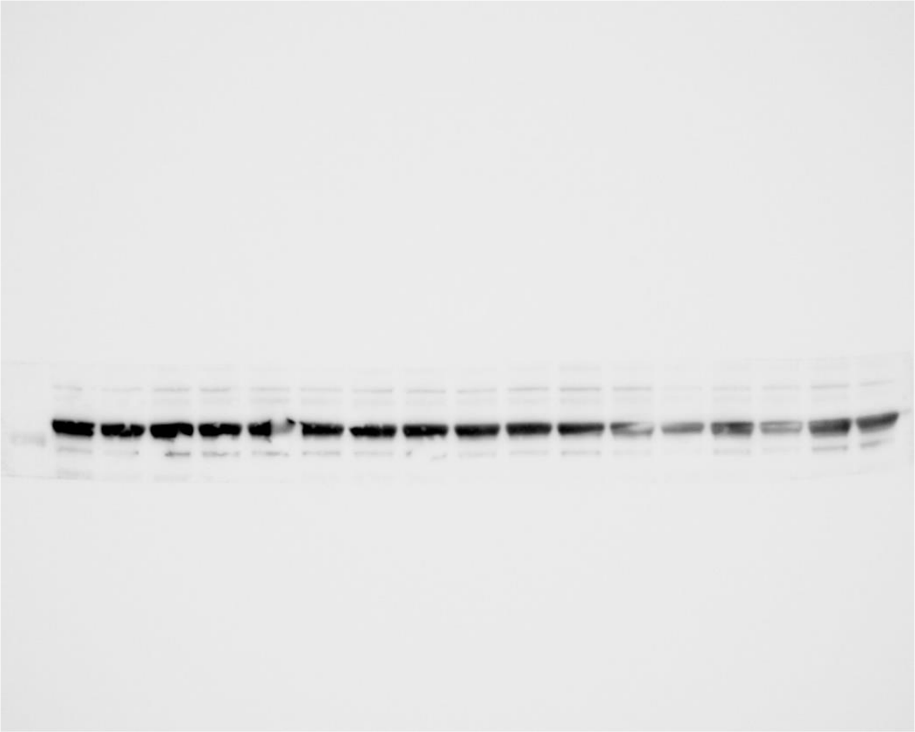

GLP1R females

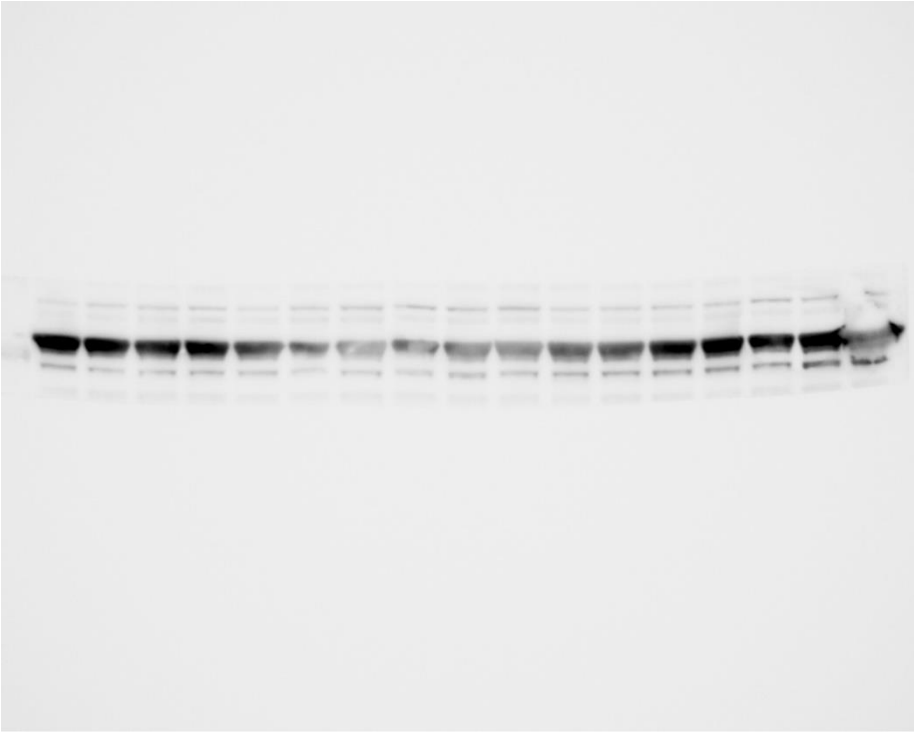

GHSR males

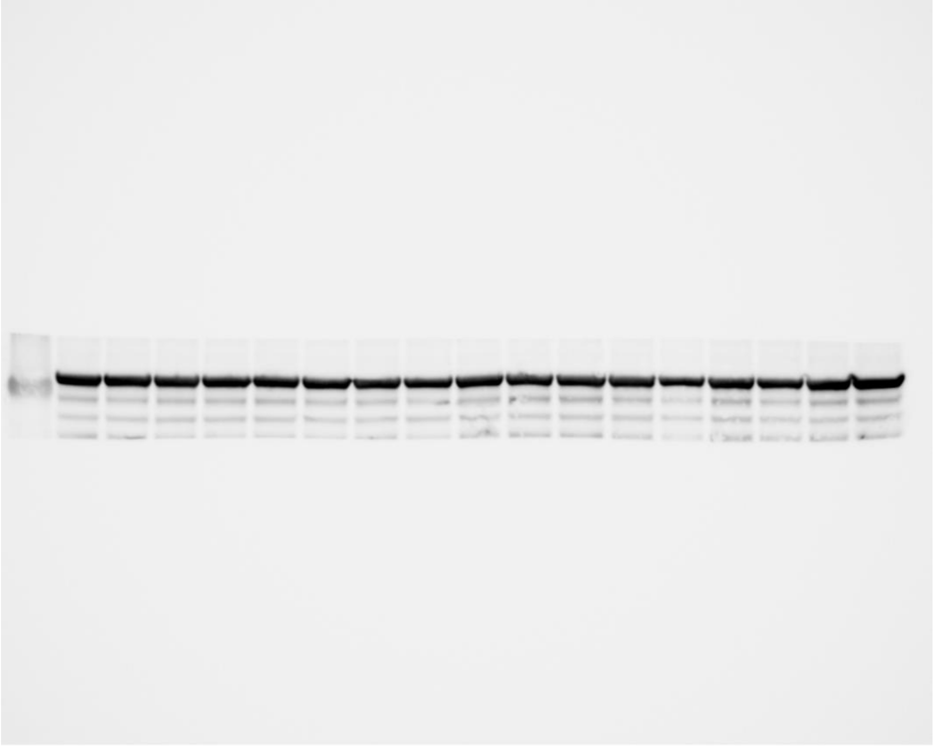

GHSR females

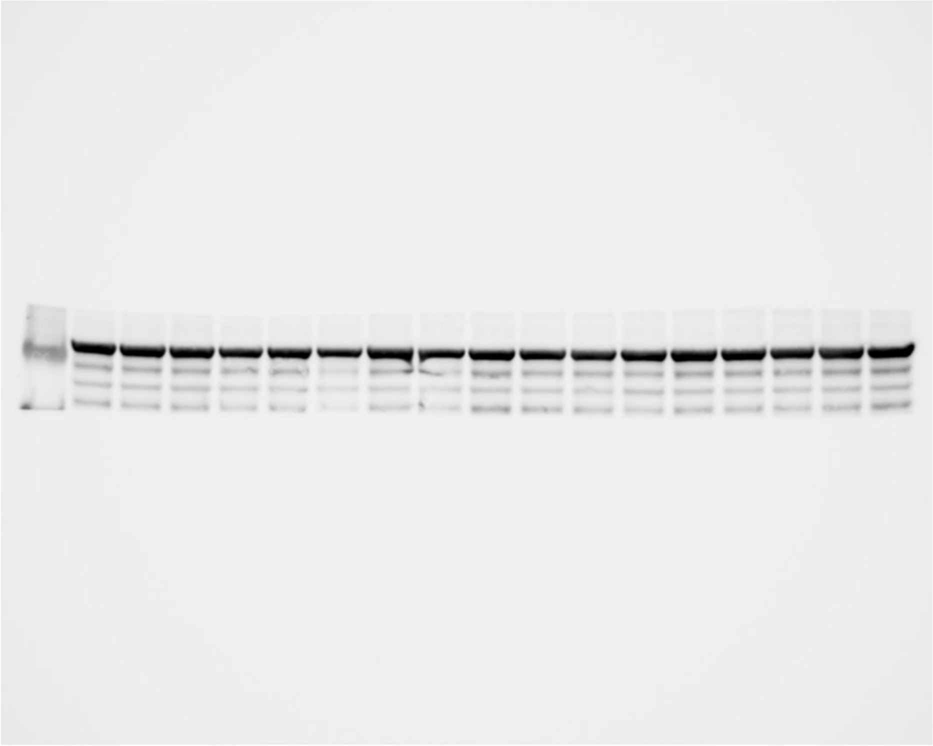

Figure S5. Unedited blots from Figure 4

γ-adaptin males

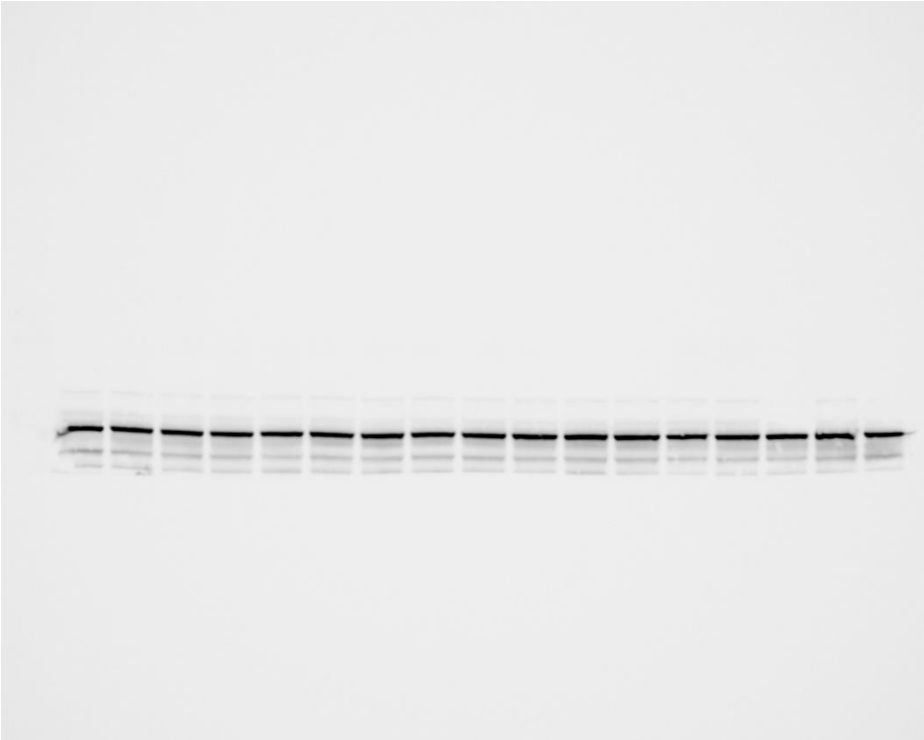

γ-adaptin females

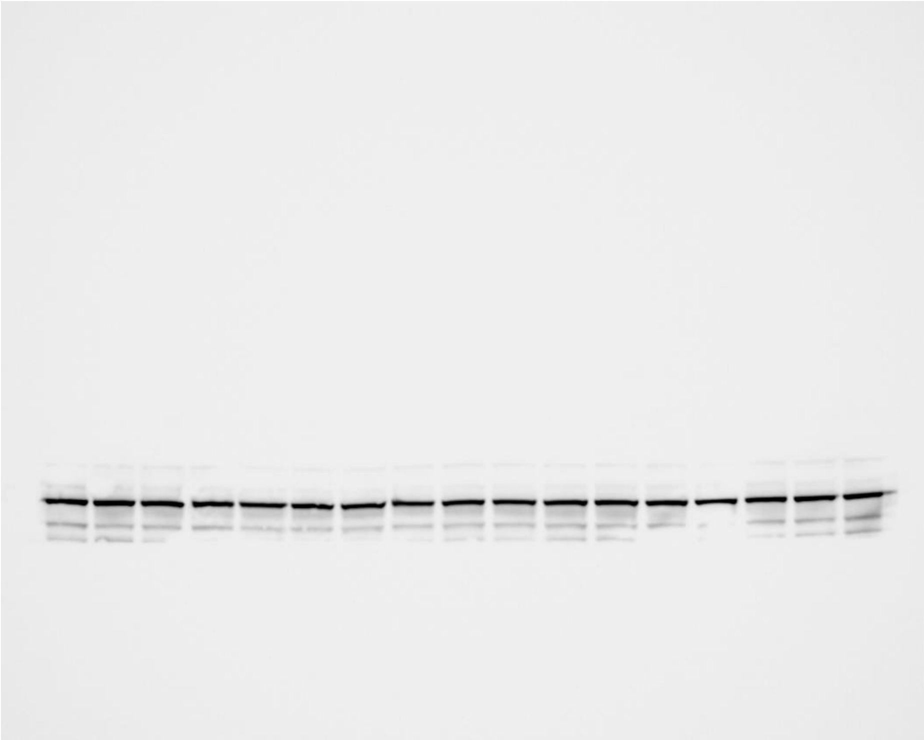

Ponceau staining males

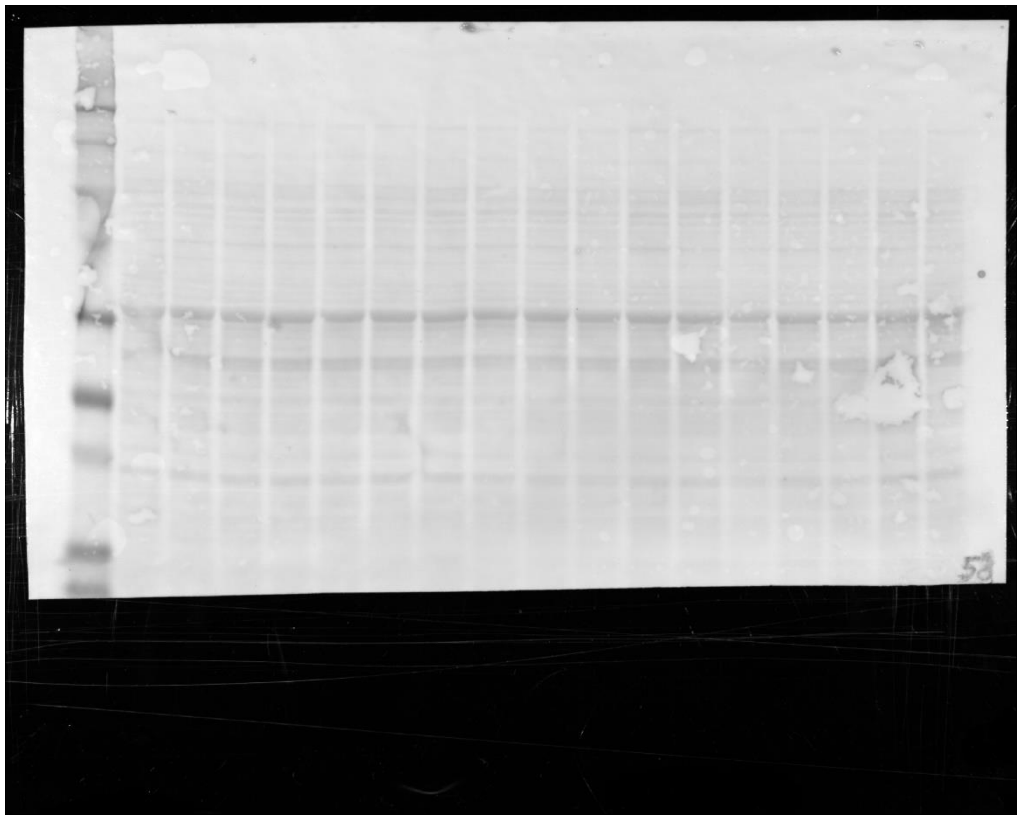

Ponceau staining females

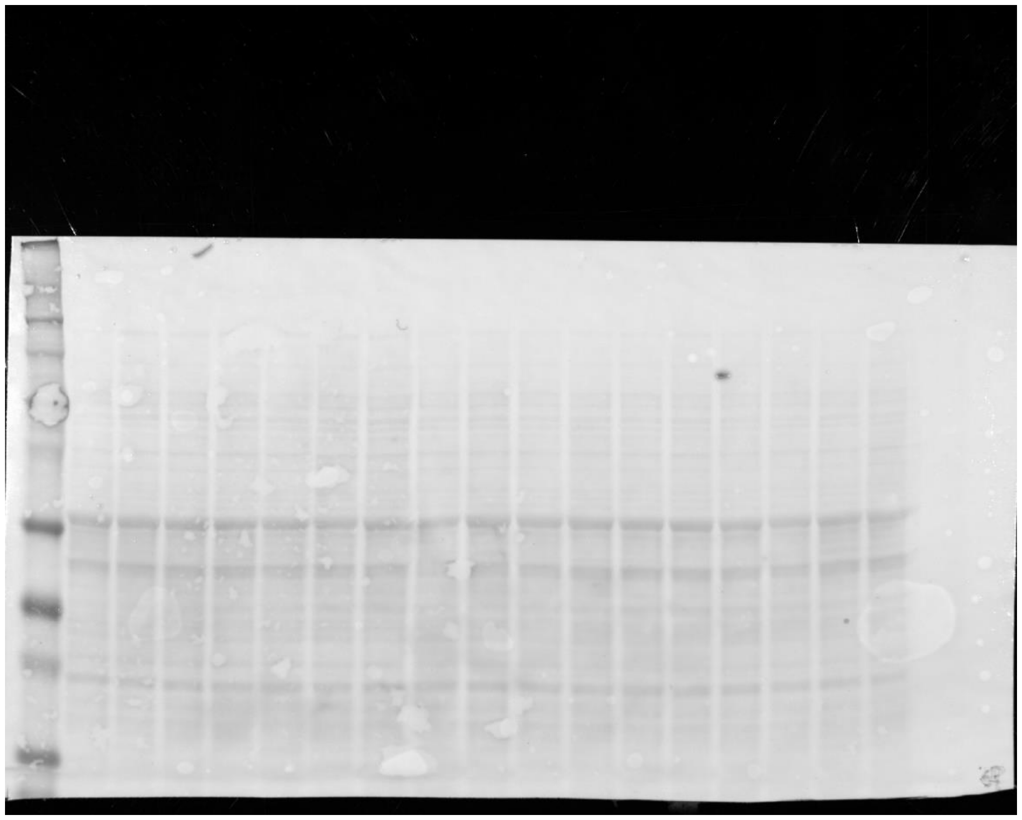

Figure S5. Unedited blots from Figure 5

TLR4 males

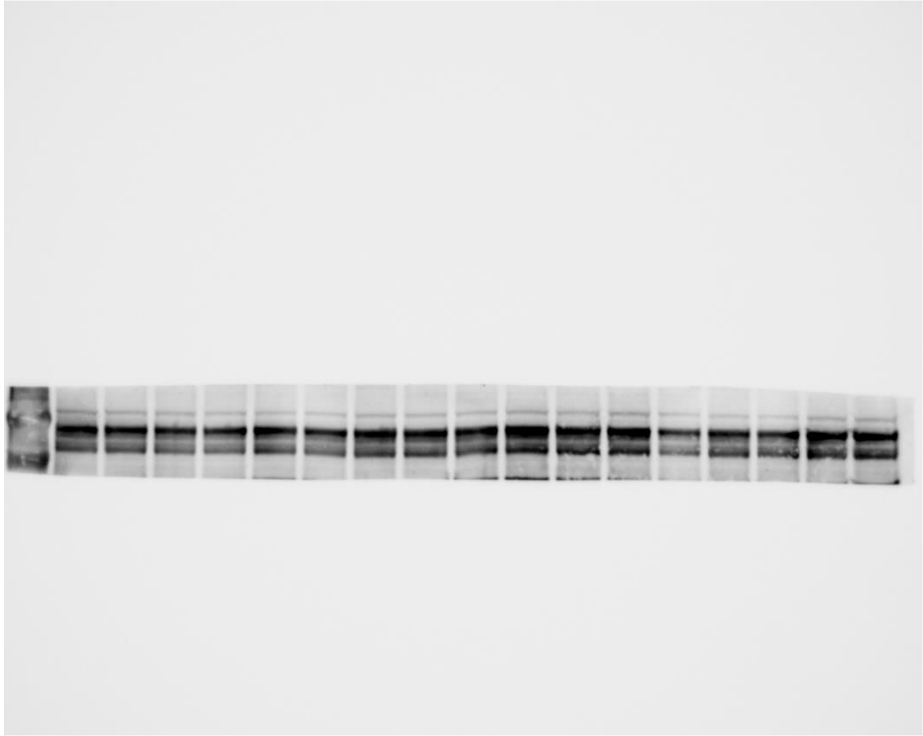

TLR4 females

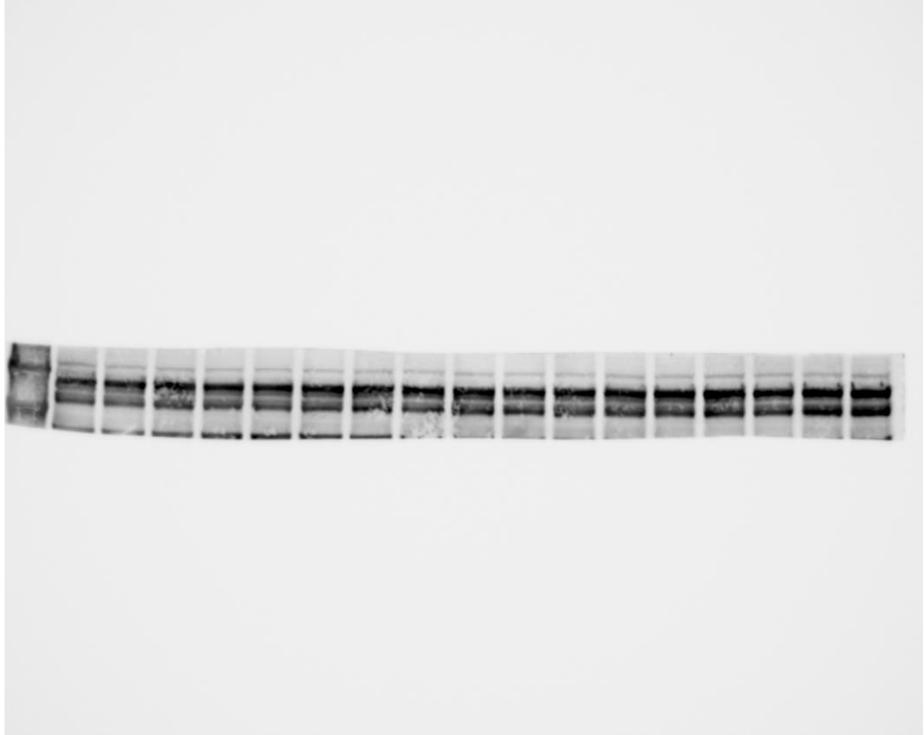

p-LEPR males

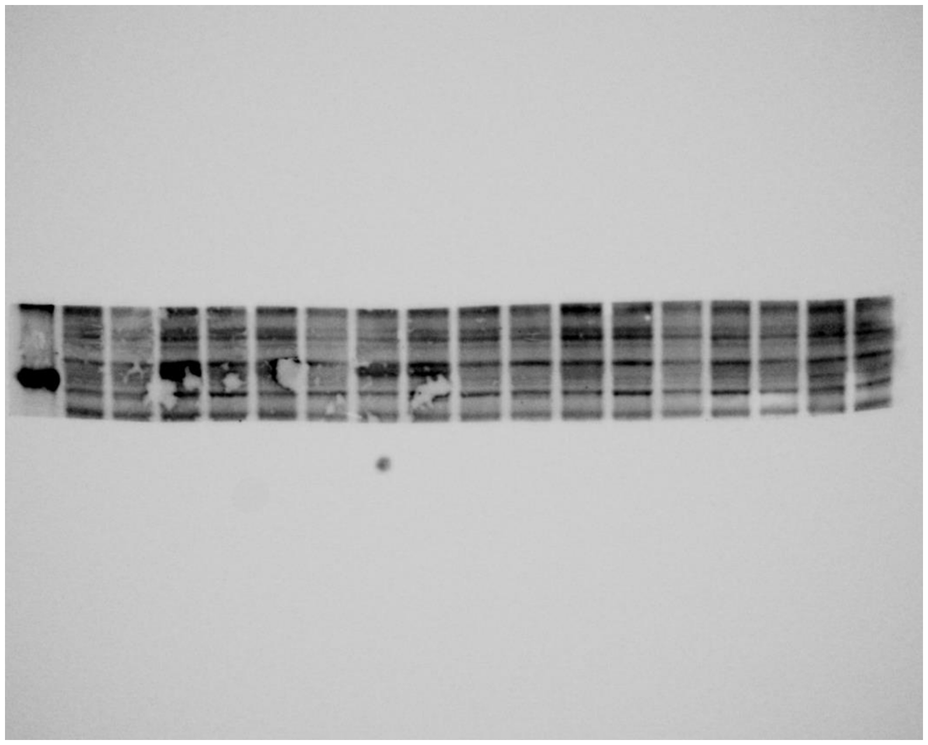

p-LEPR females

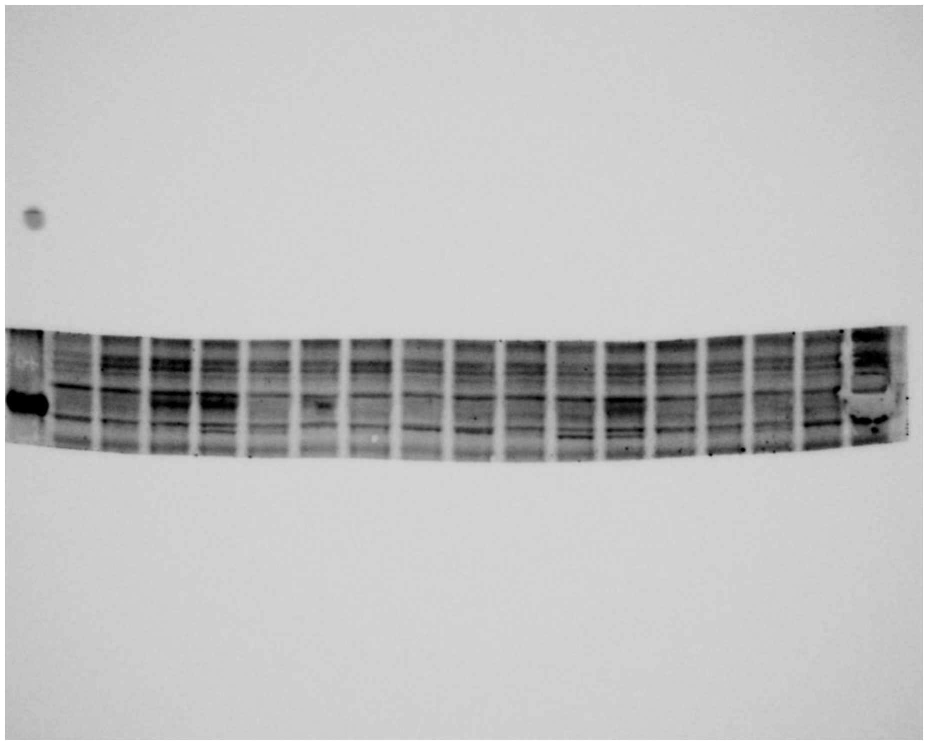

Figure S5. Unedited blots from Figure 5

LepR machos

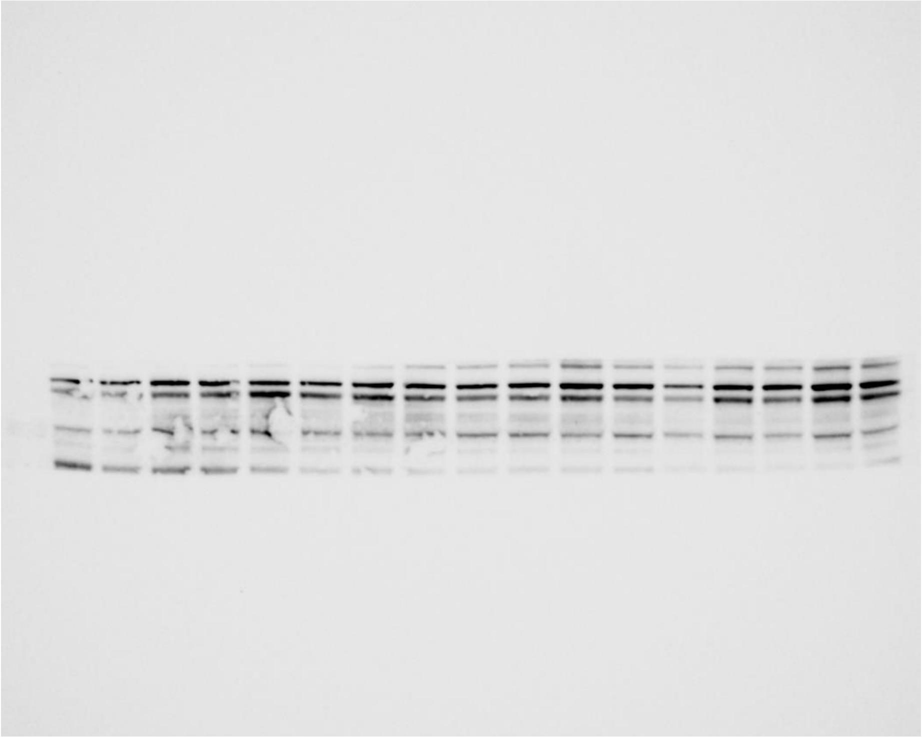

LepR hembras

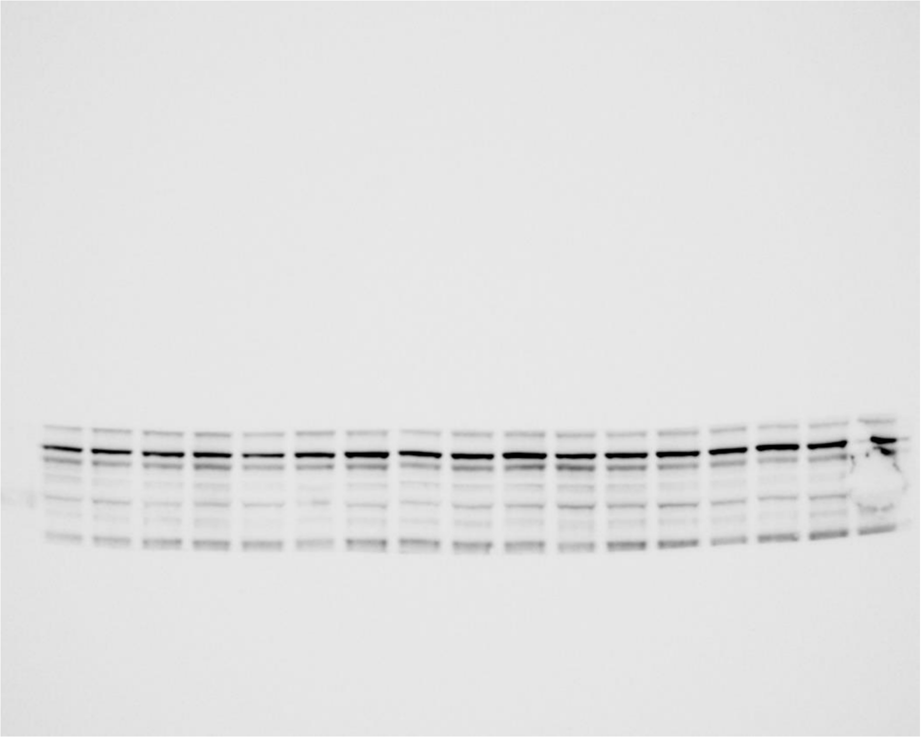

γ-adaptin males

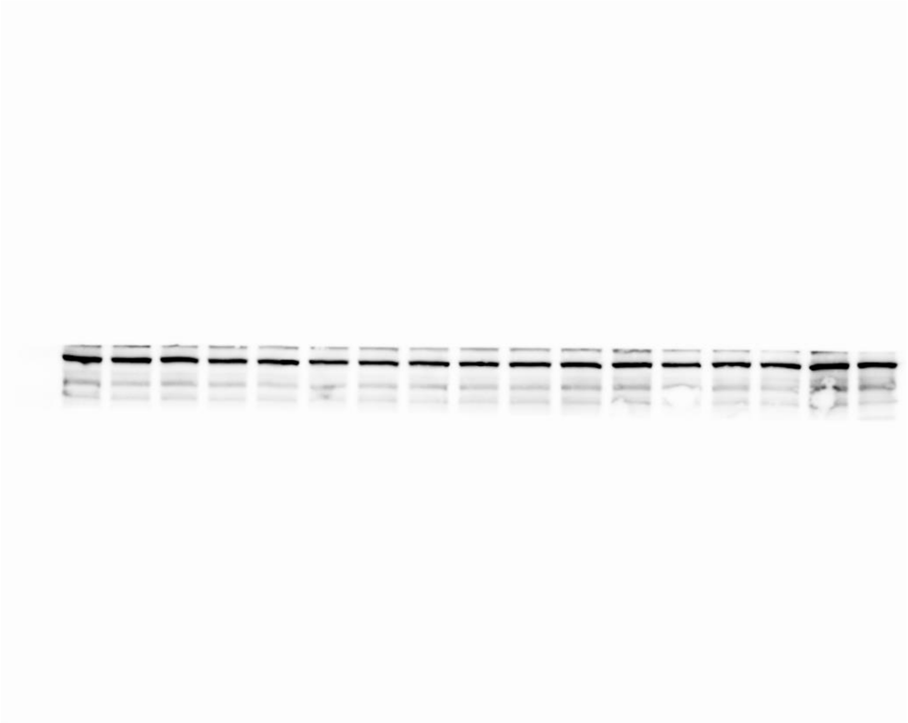

γ-adaptin females

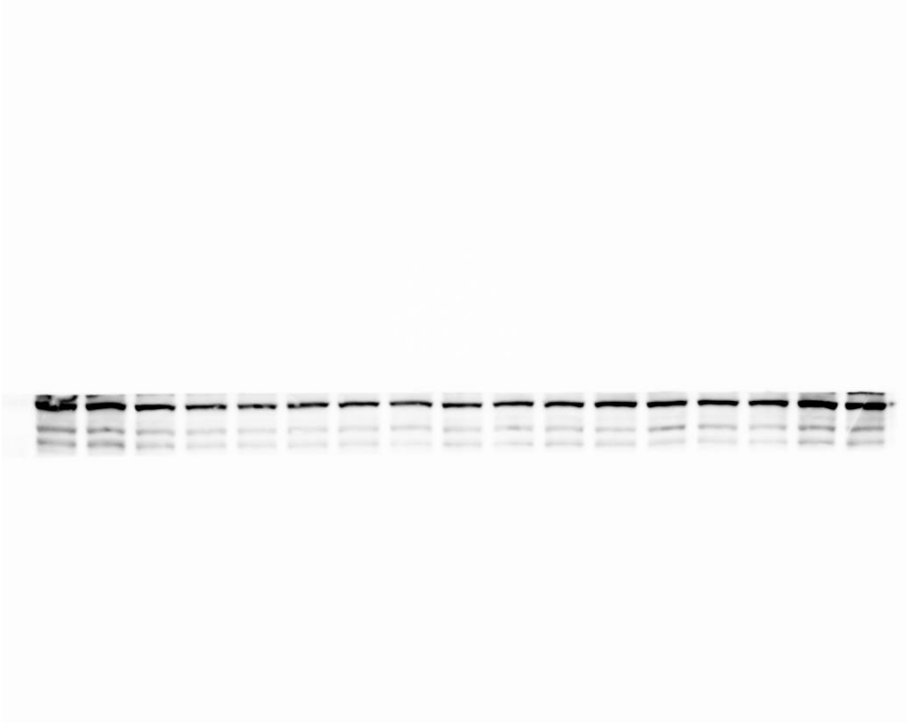

Figure S5. Unedited blots from Figure 5

Ponceau staining males

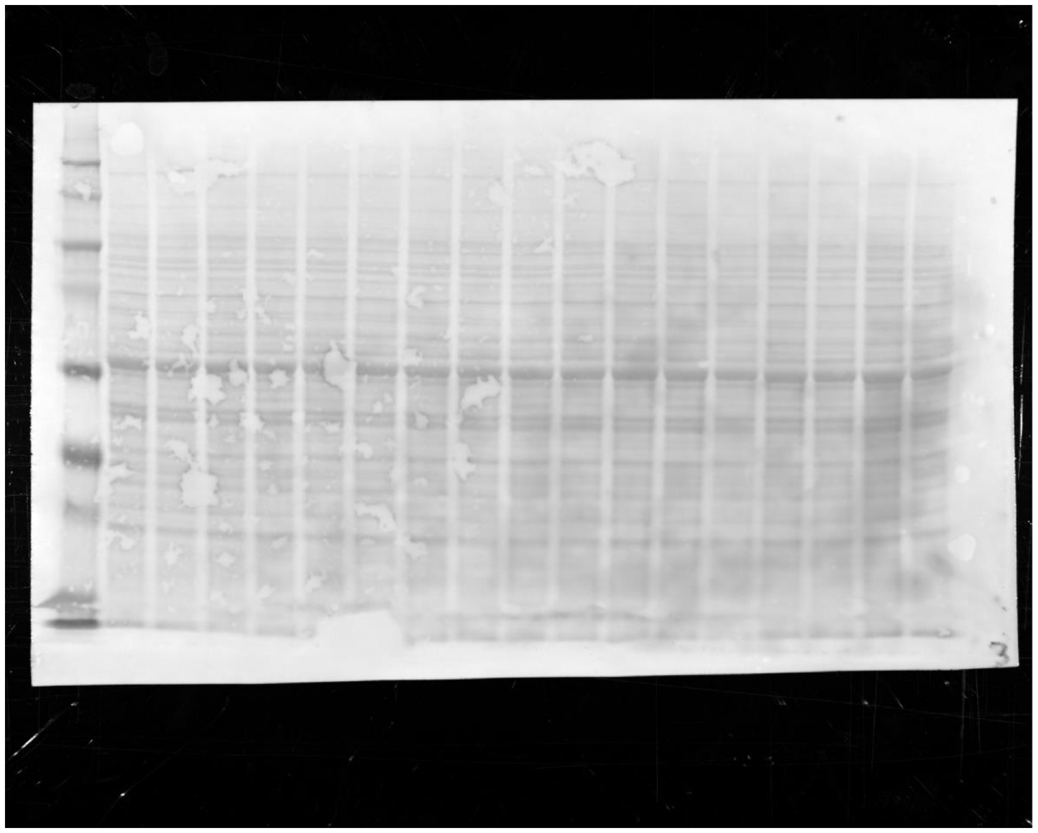

Ponceau staining females

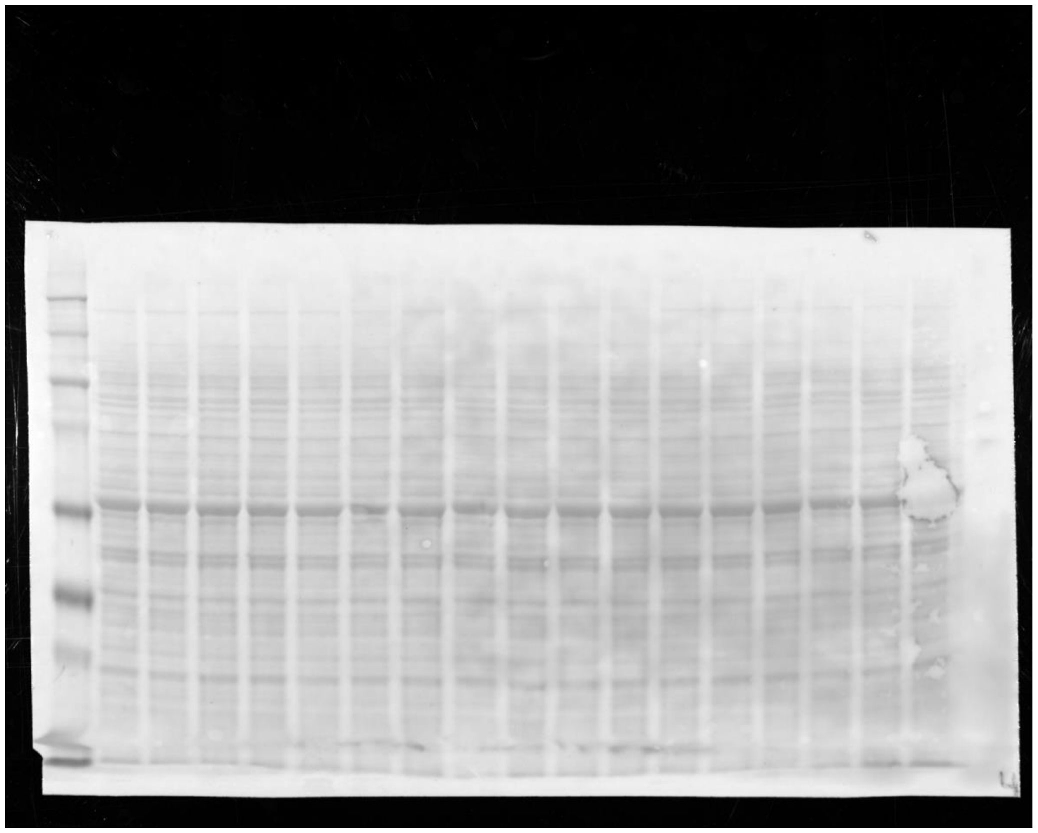

Figure S5. Unedited blots from Figure 6

IRS1 p(Ser612) males

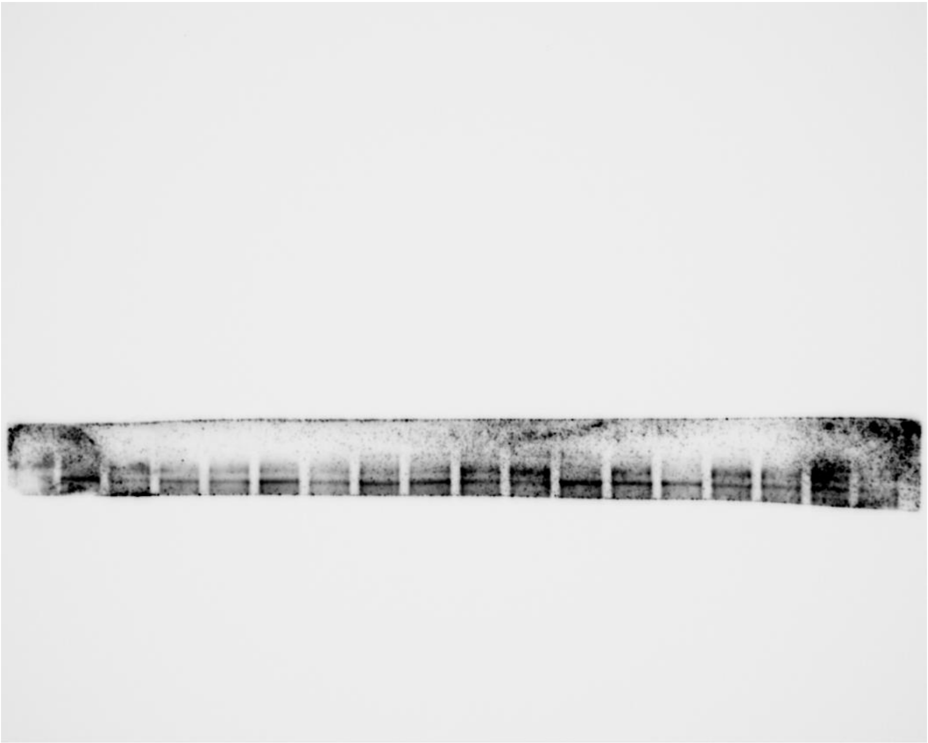

IRS1 p(Ser612) females

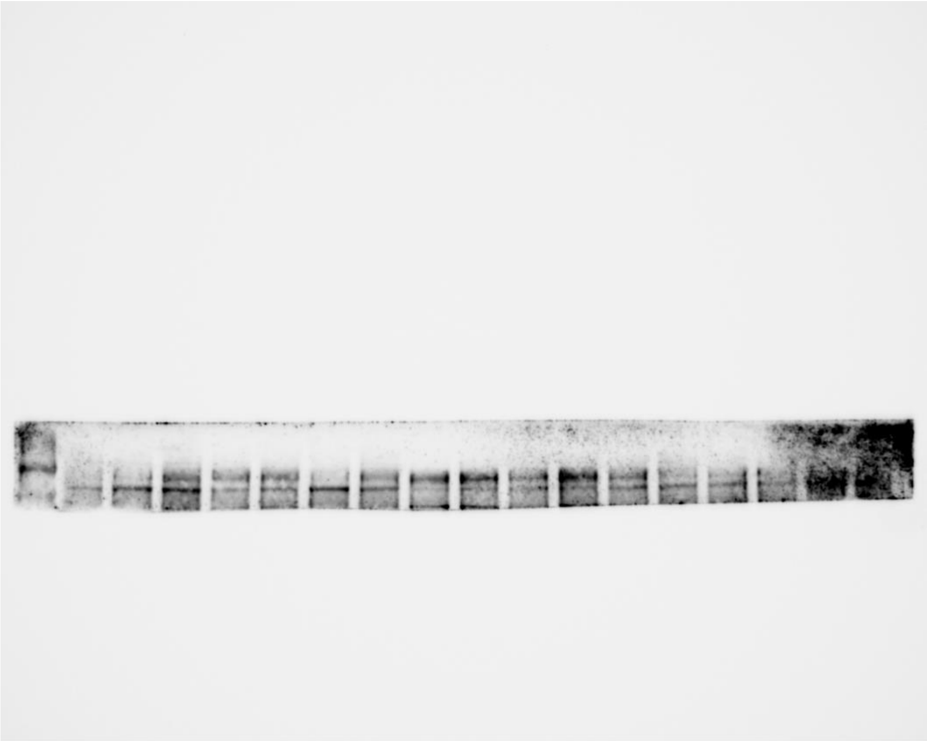

IRS1 p(Tyr892) males

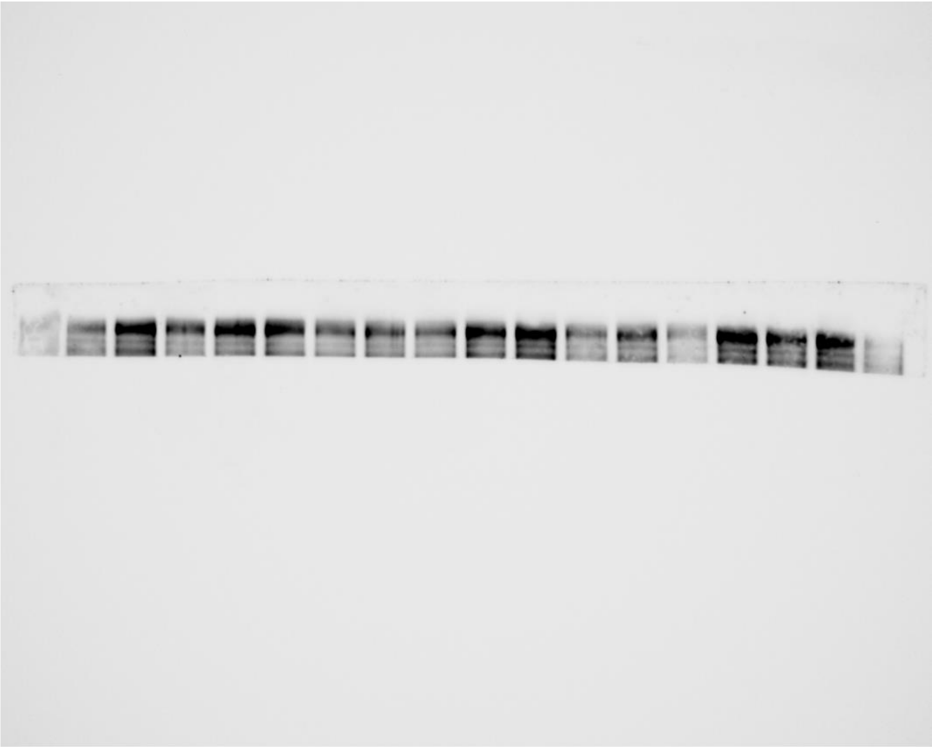

IRS1 p(Tyr892) females

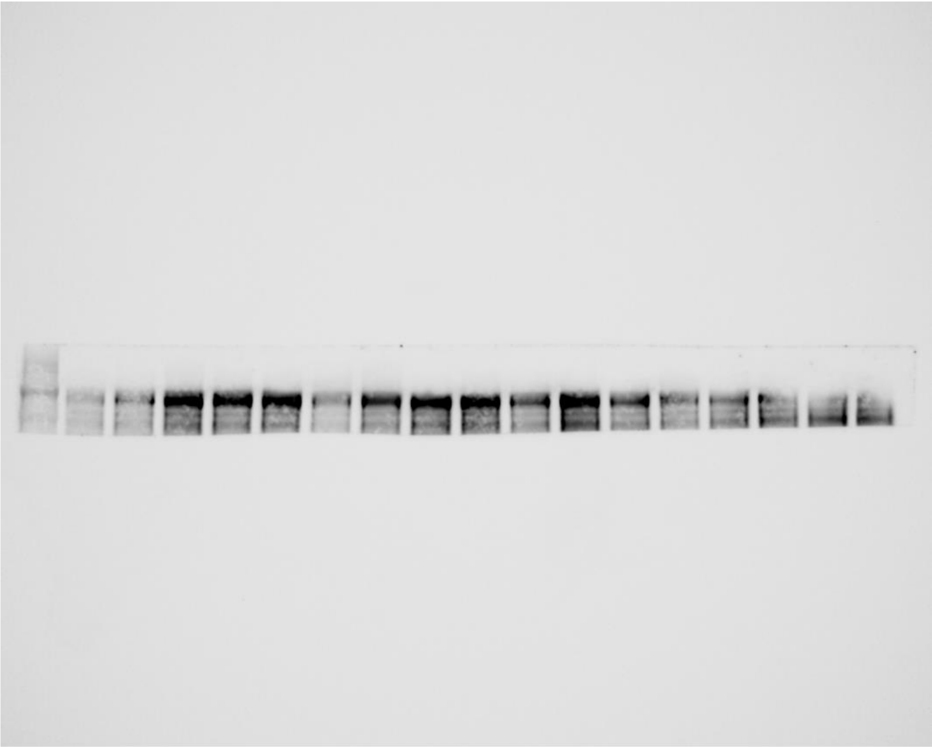

Figure S5. Unedited blots from Figure 6

IRS1 males

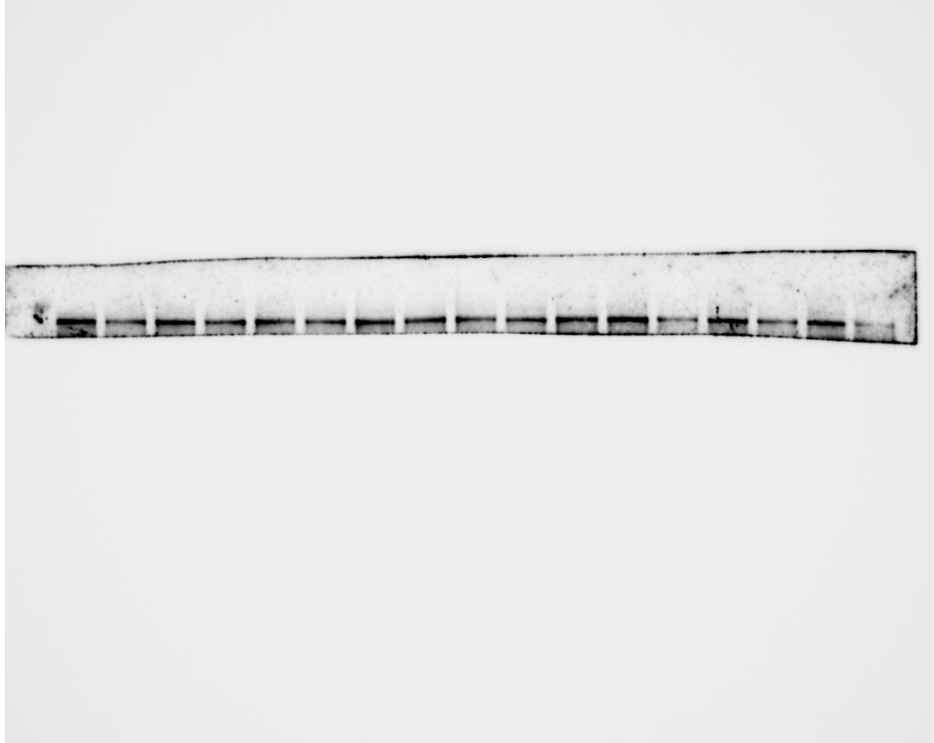

IRS1 females

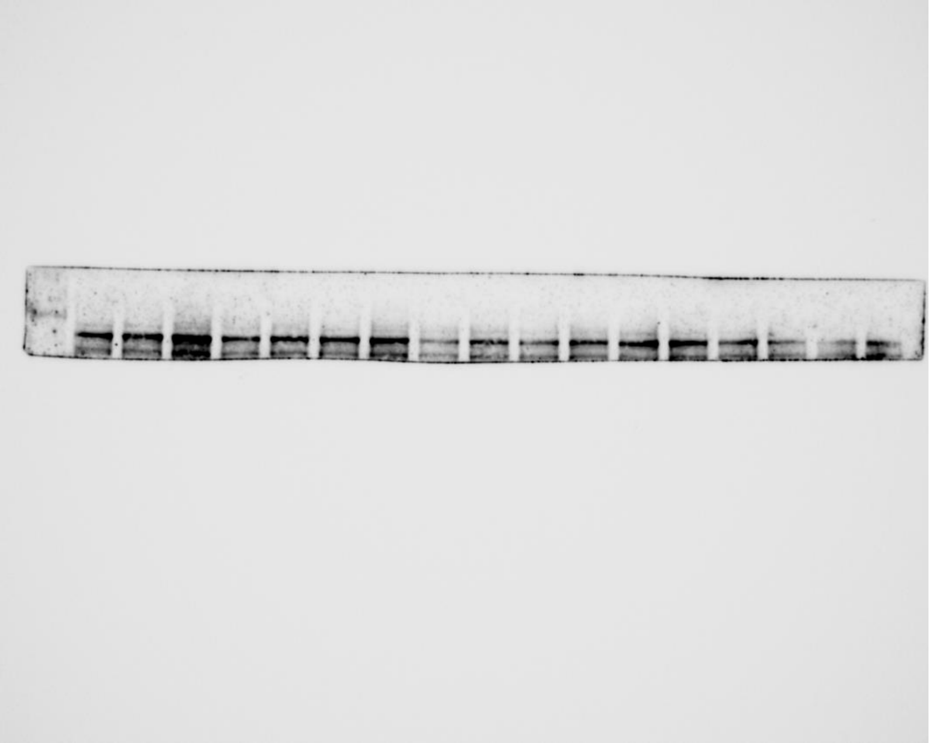

p-STAT5 males

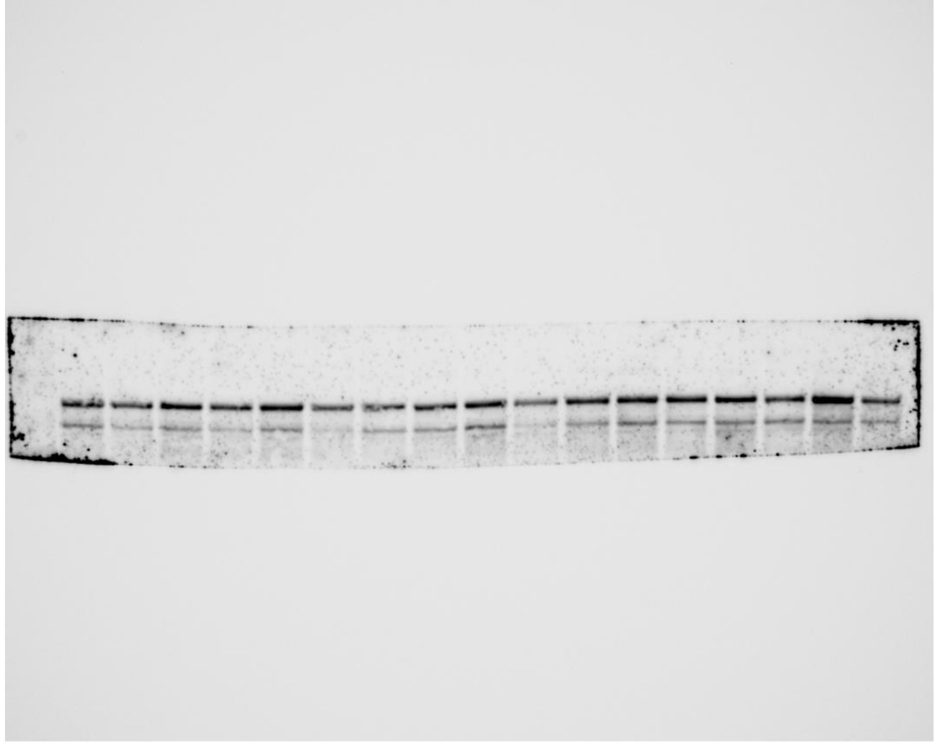

p-STAT5 females

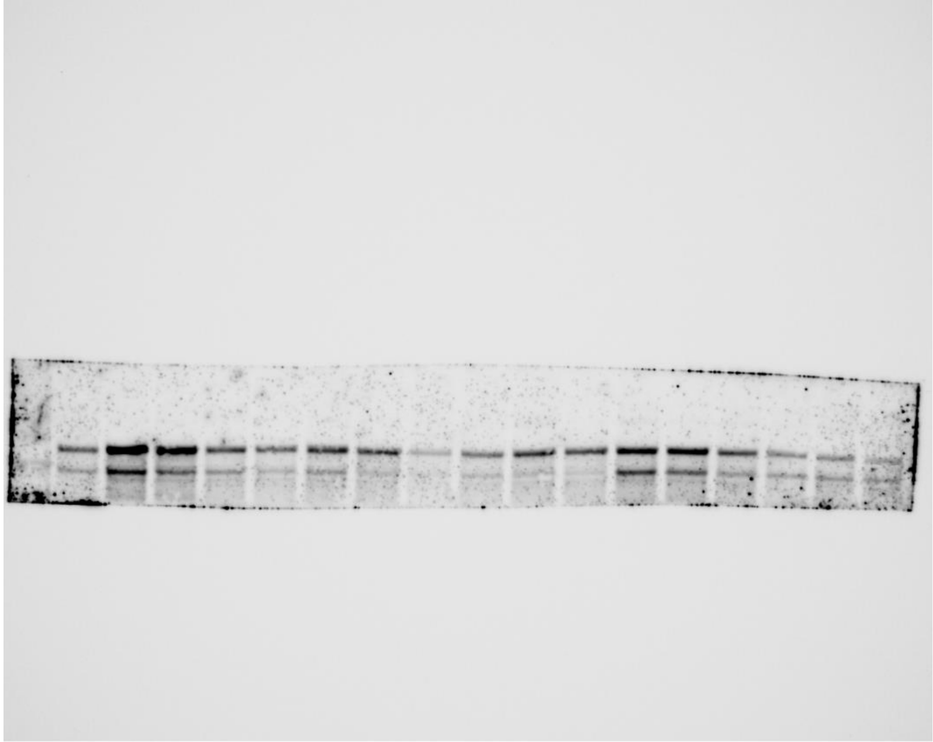

Figure S5. Unedited blots from Figure 6

STAT5 males

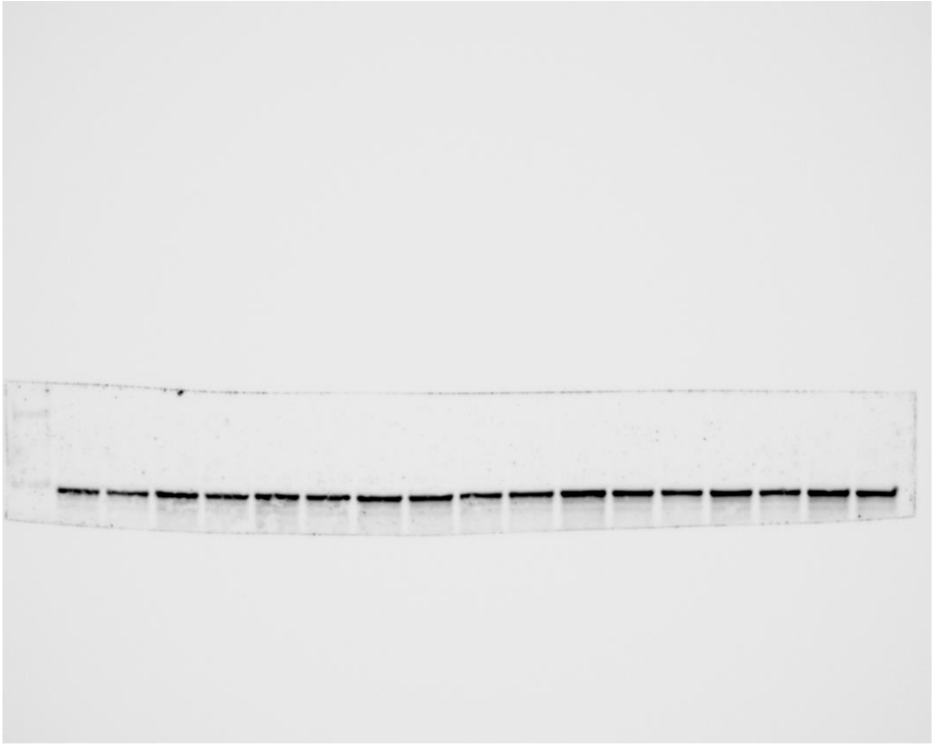

STAT5 females

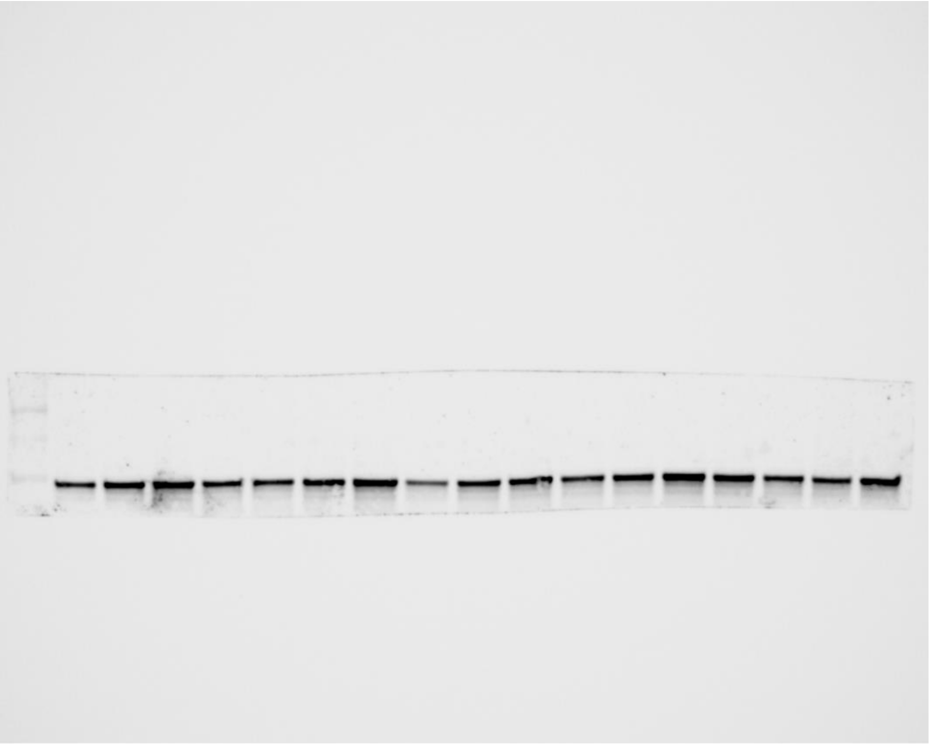

p-STAT3 males

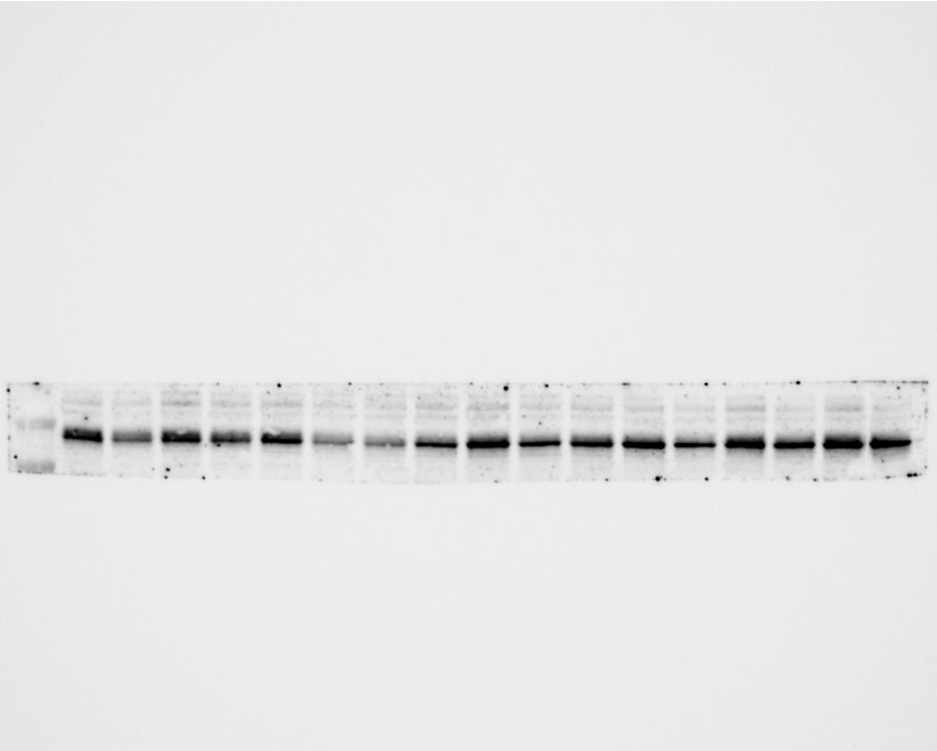

p-STAT3 females

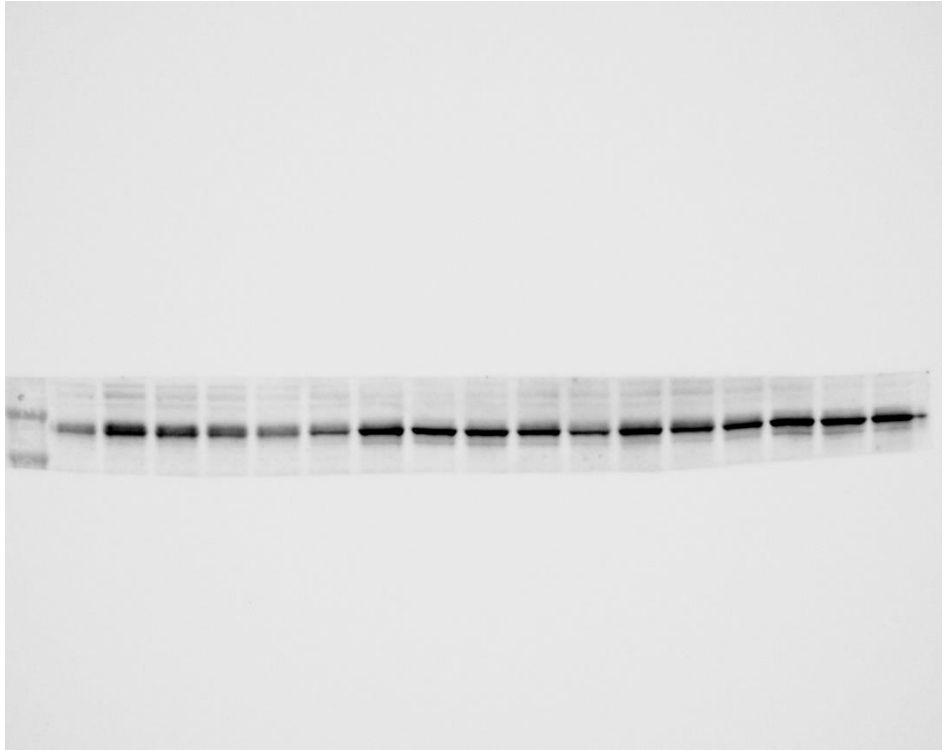

Figure S5. Unedited blots from Figure 6

STAT3 males

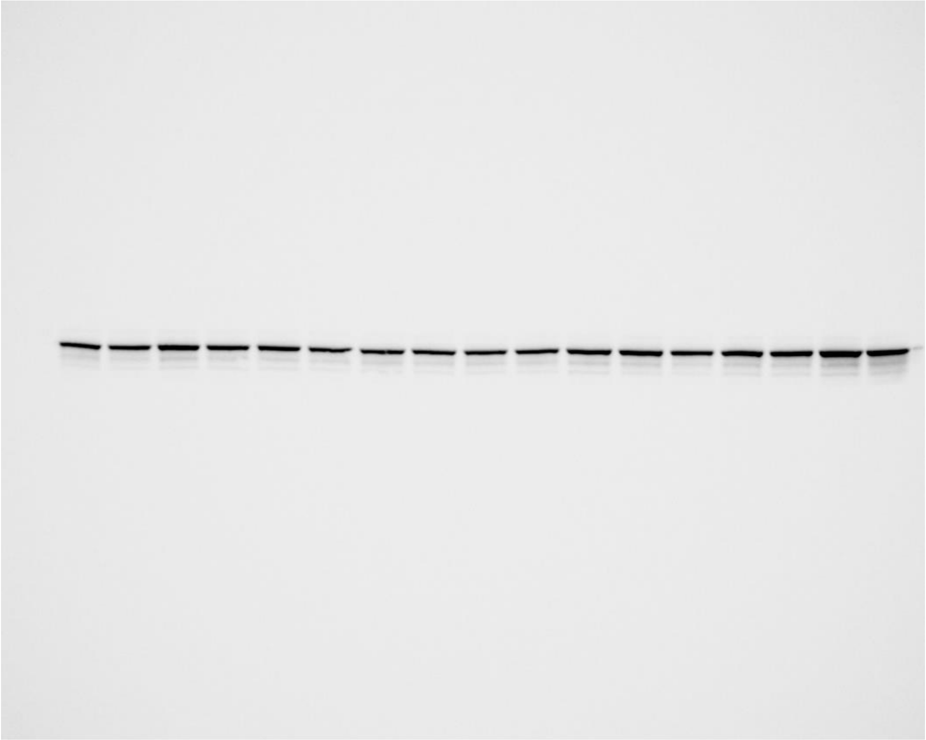

STAT3 females

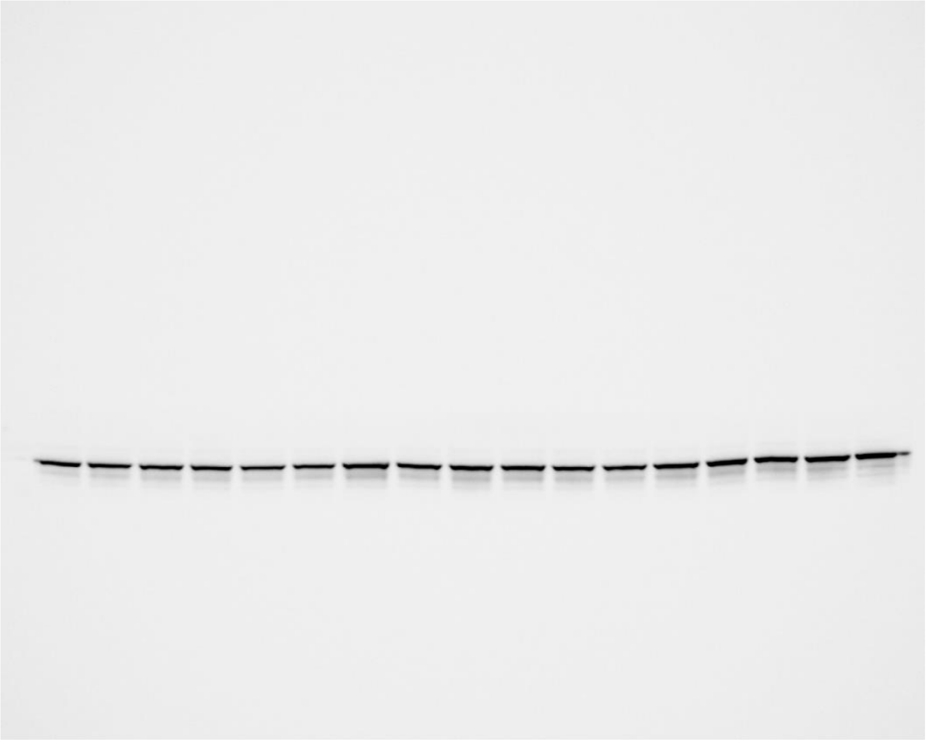

γ-adaptin males

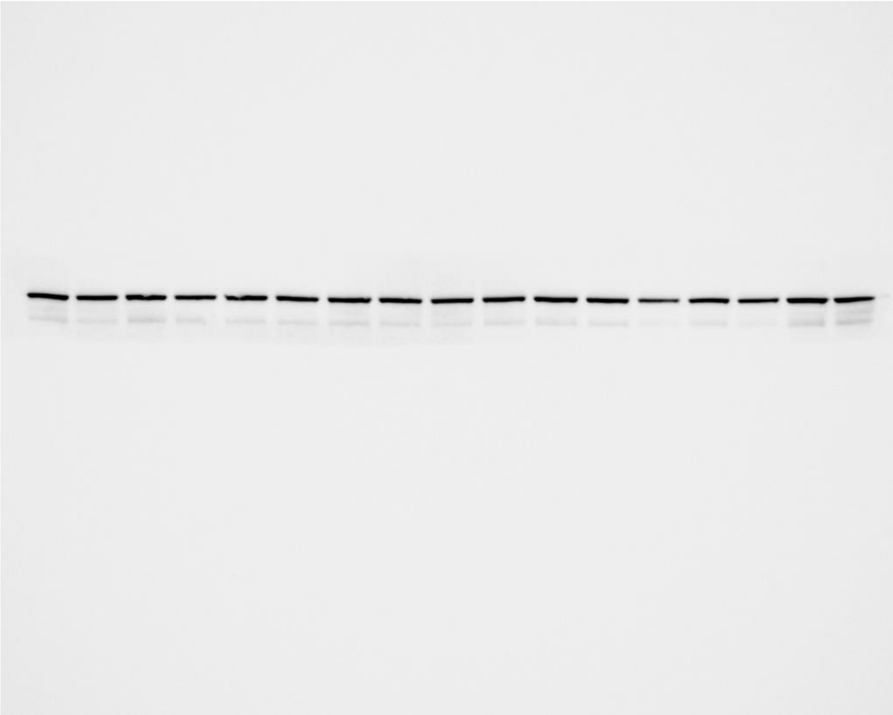

γ-adaptin females

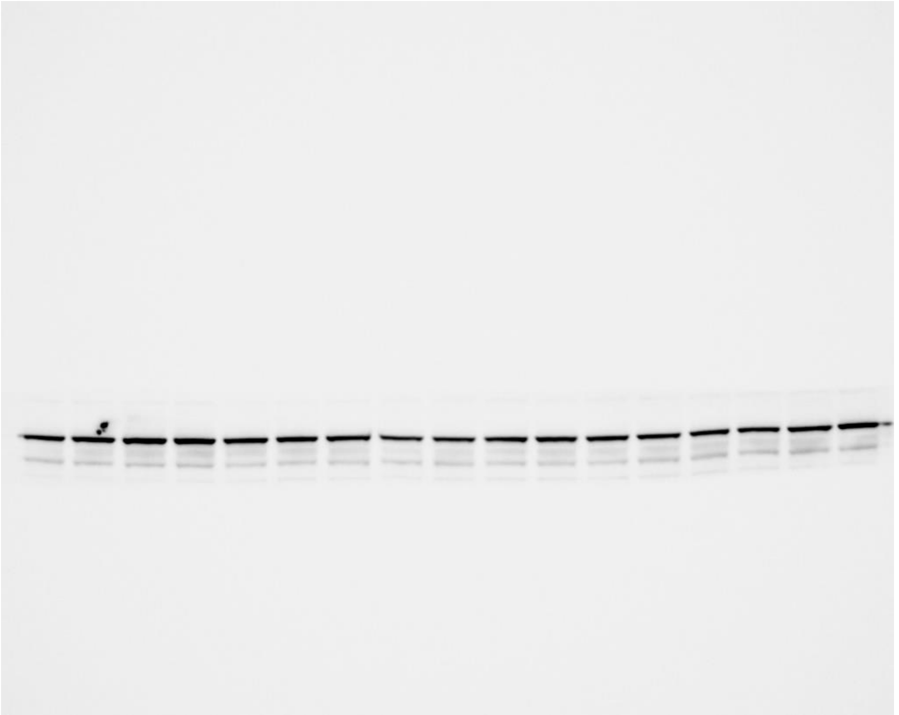

Figure S5. Unedited blots from Figure 6

Ponceau staining males

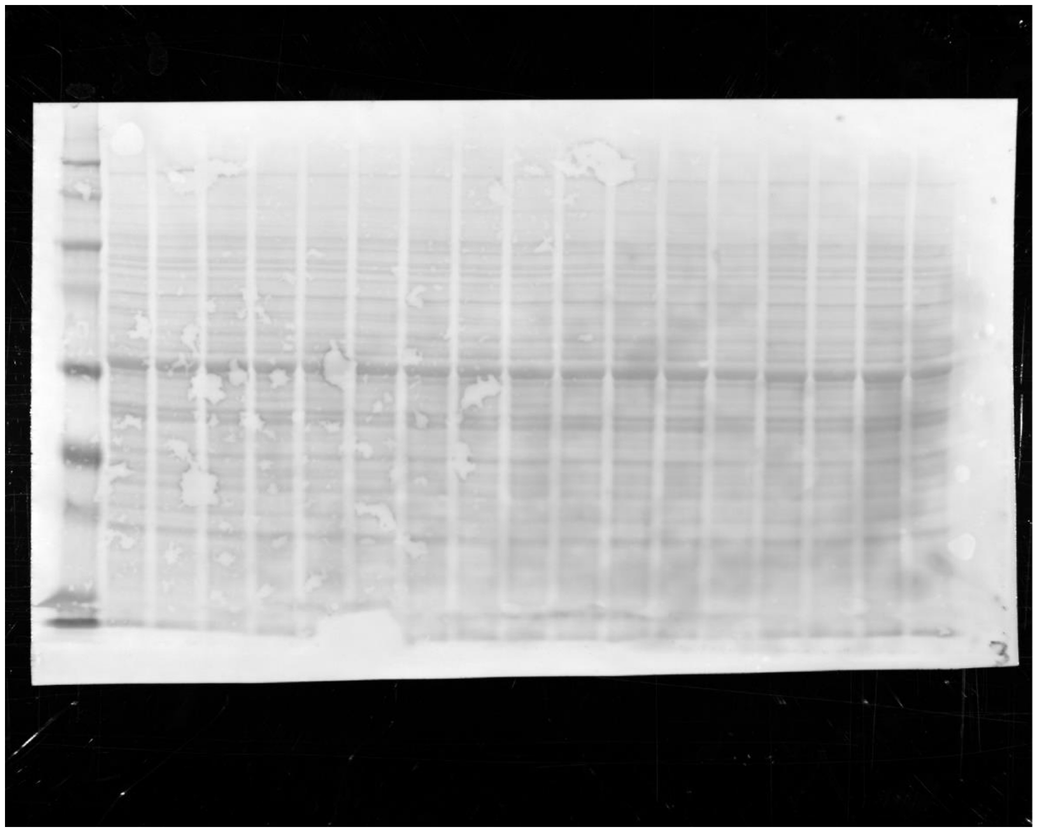

Ponceau staining females

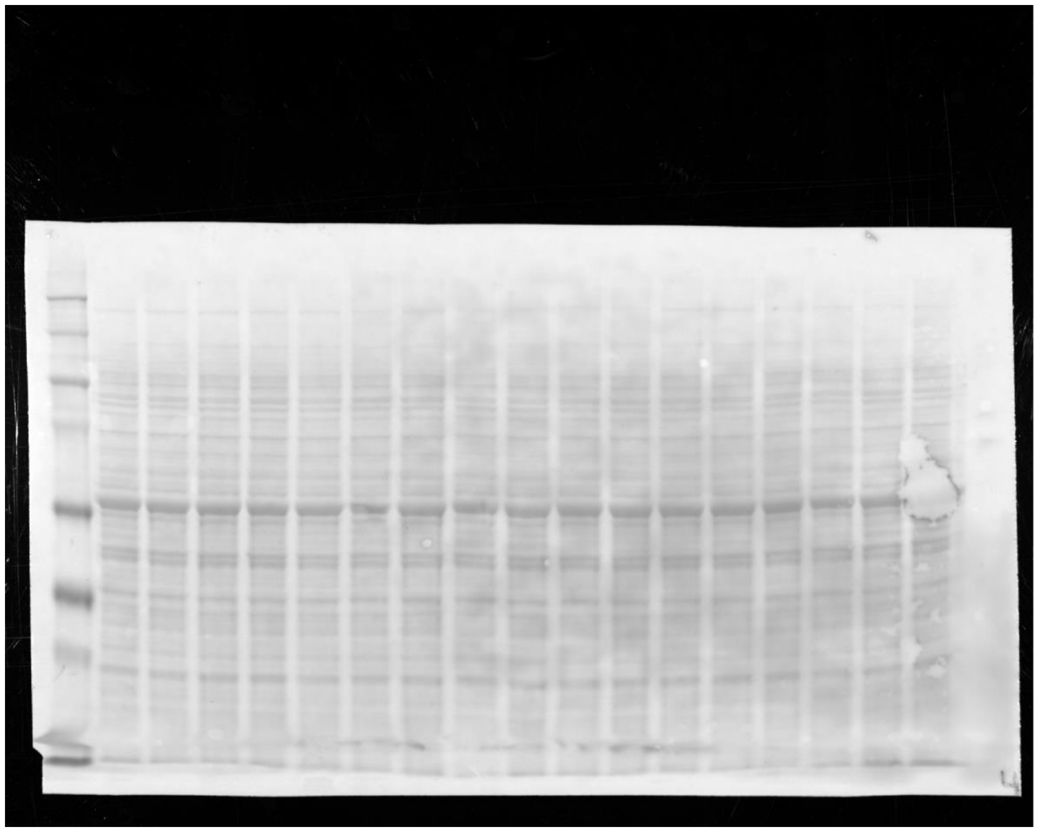

Figure S5. Unedited blots from Figure 7

p-mTOR males

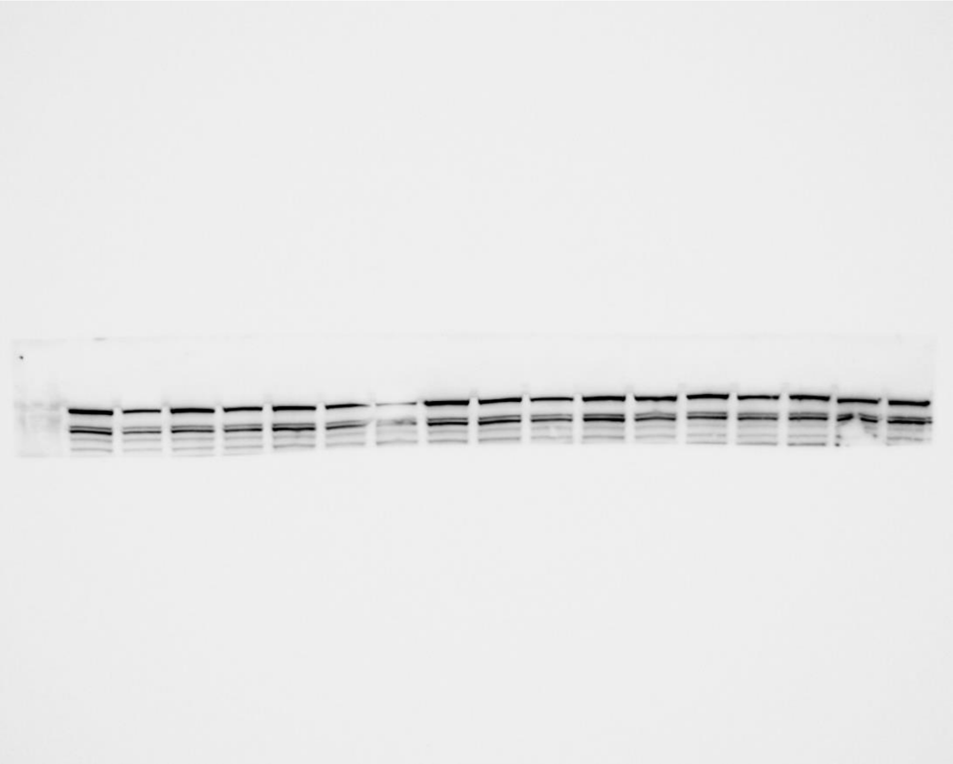

p-mTOR females

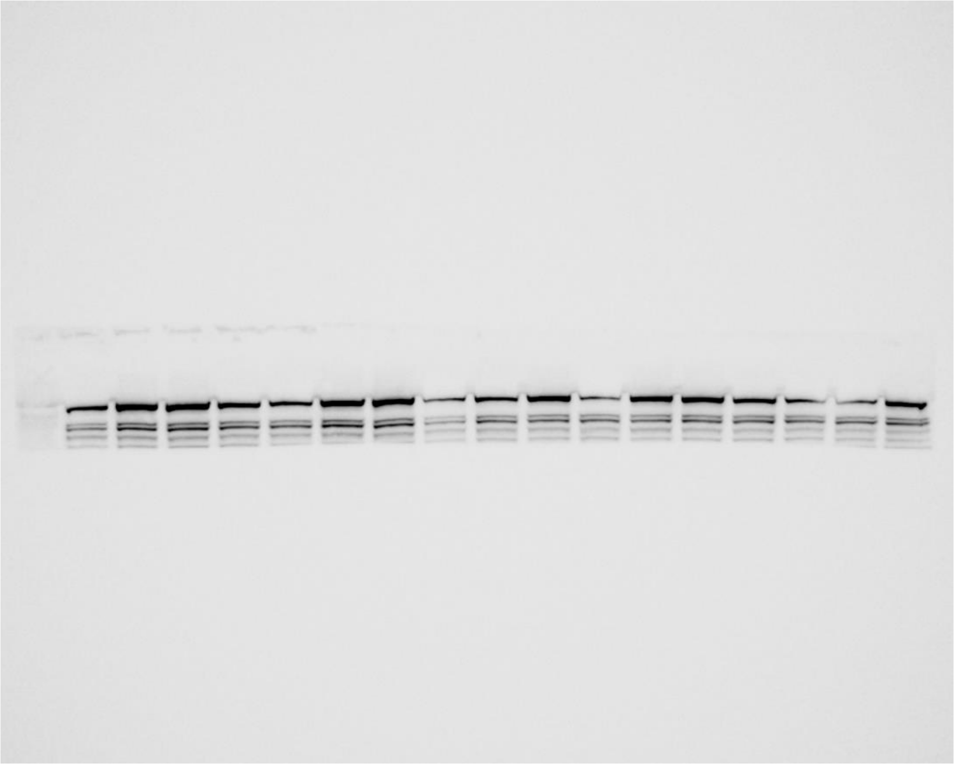

mTOR males

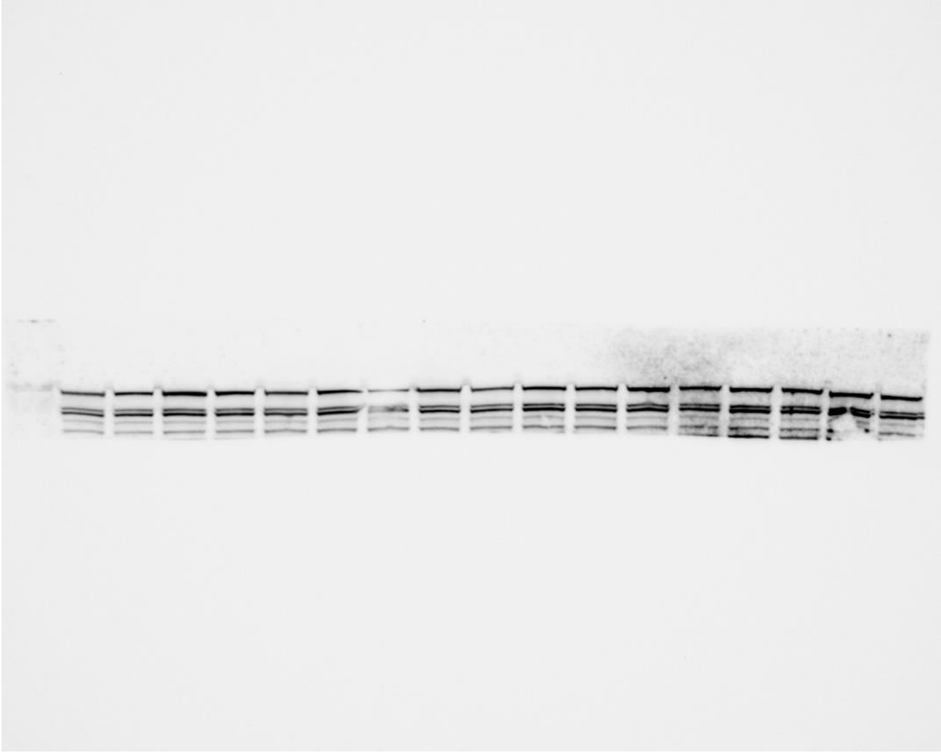

mTOR females

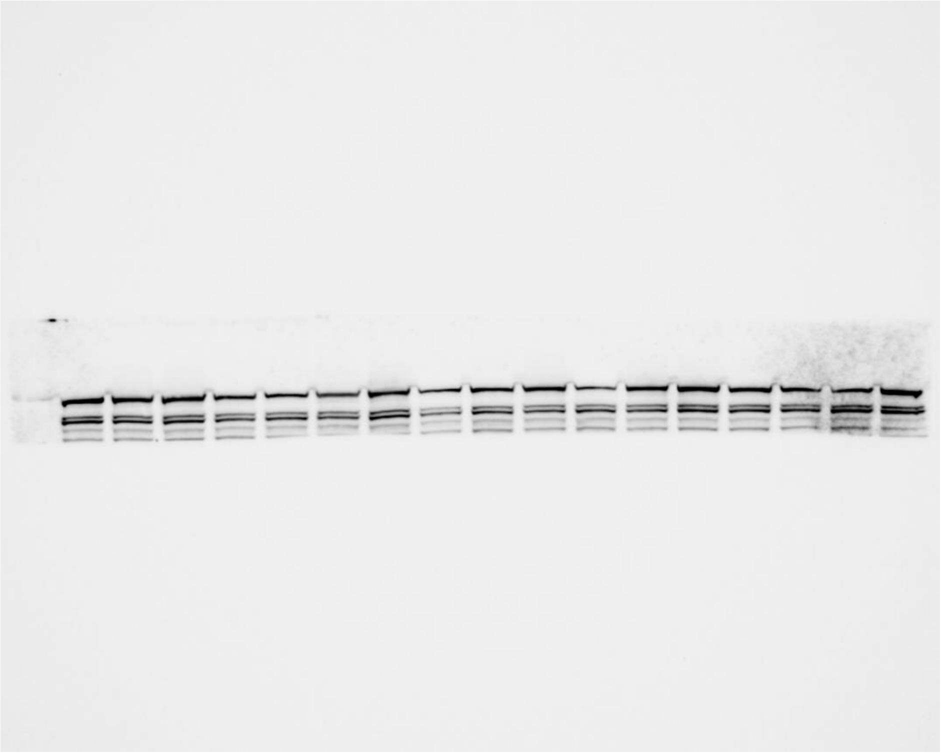

Figure S5. Unedited blots from Figure 7

p-AMPK males

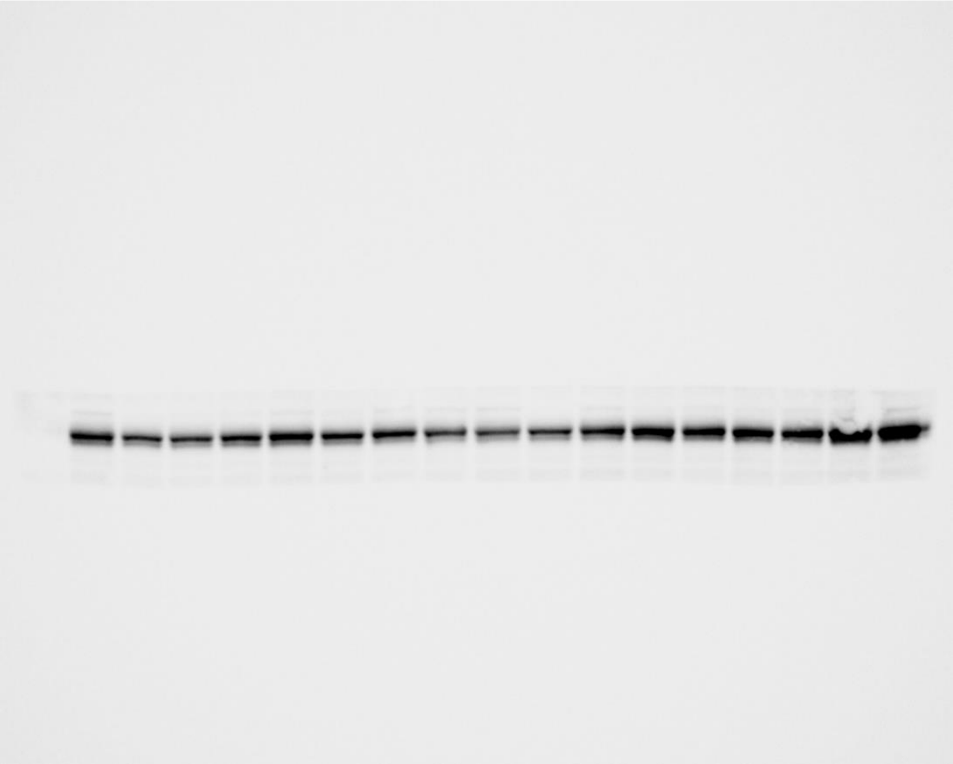

p-AMPK females

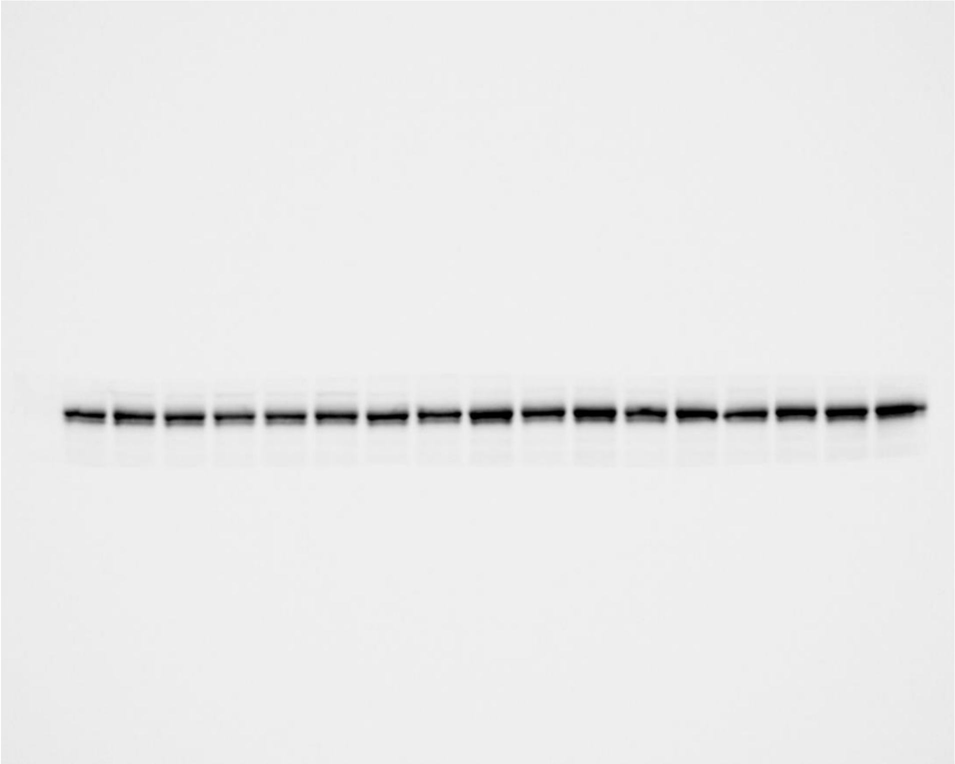

AMPK males

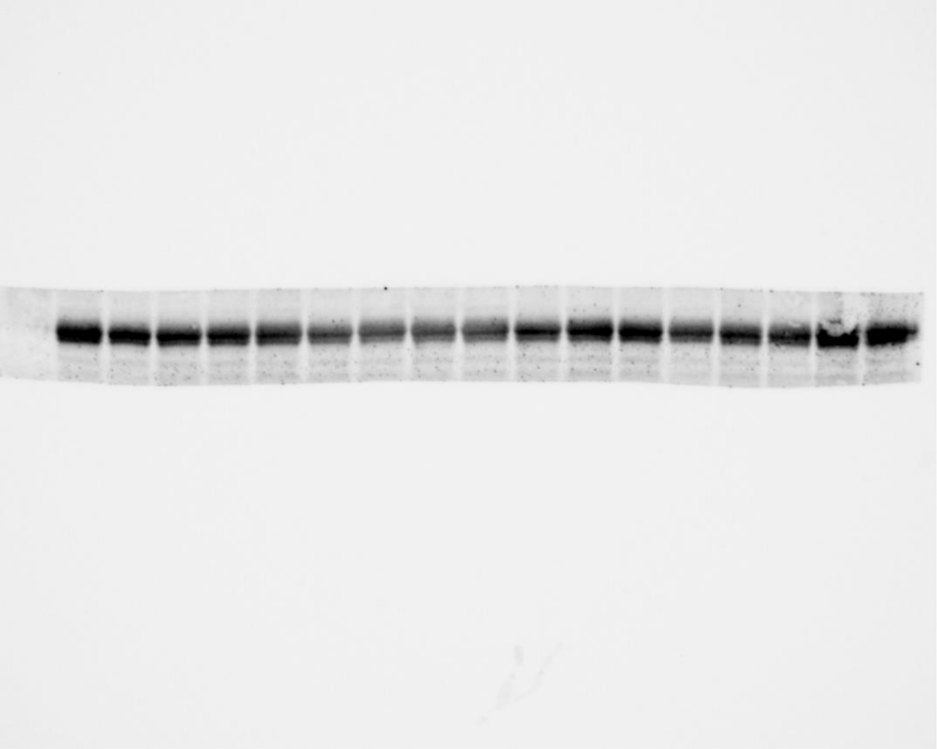

AMPK females

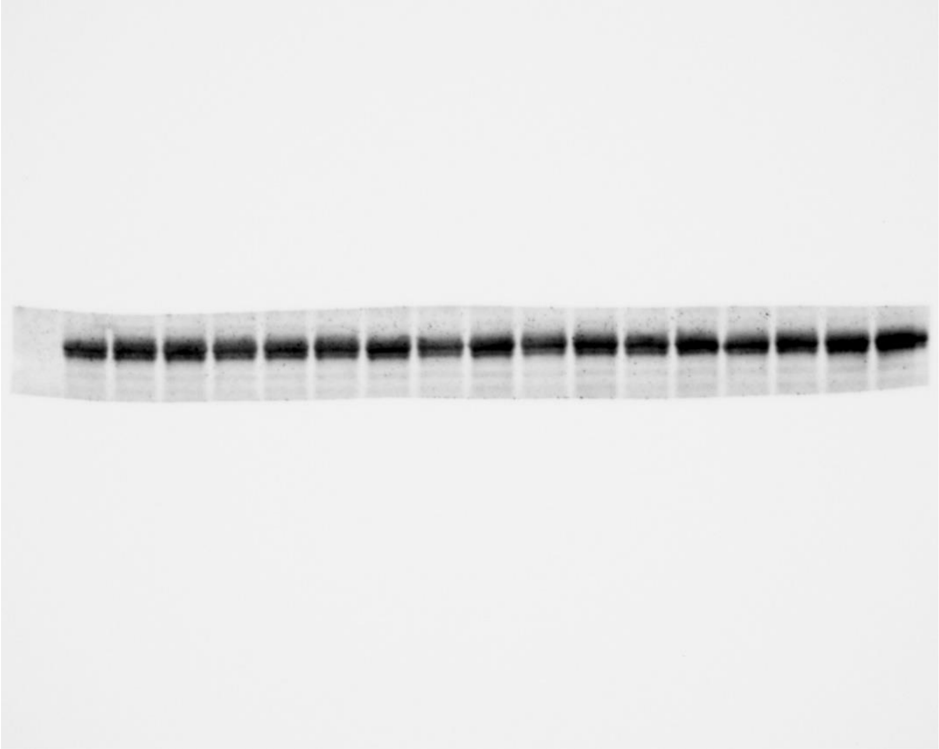

Figure S5. Unedited blots from Figure 7

p-ERK1 & p-ERK2

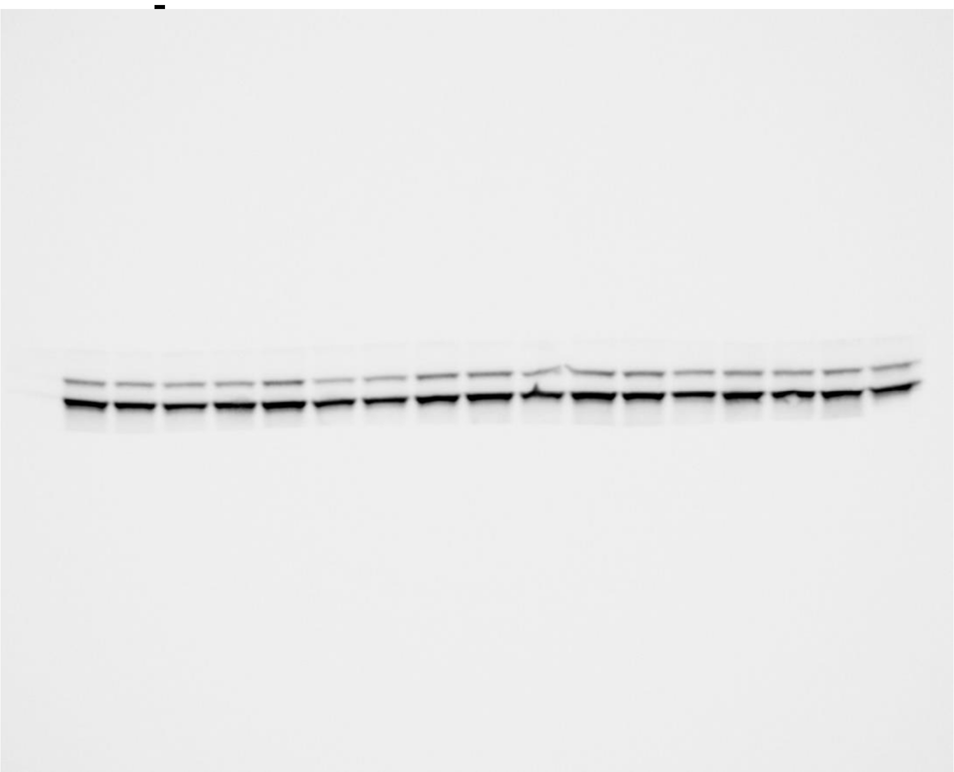

p-ERK1 & p-ERK2 females

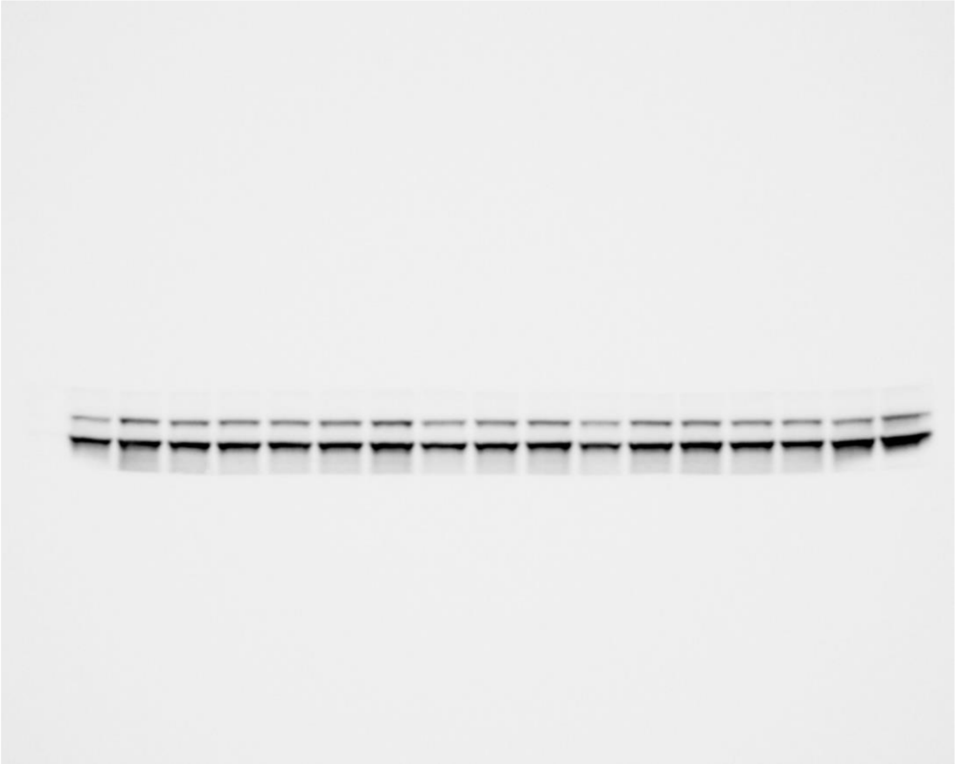

ERK1 & ERK2 males

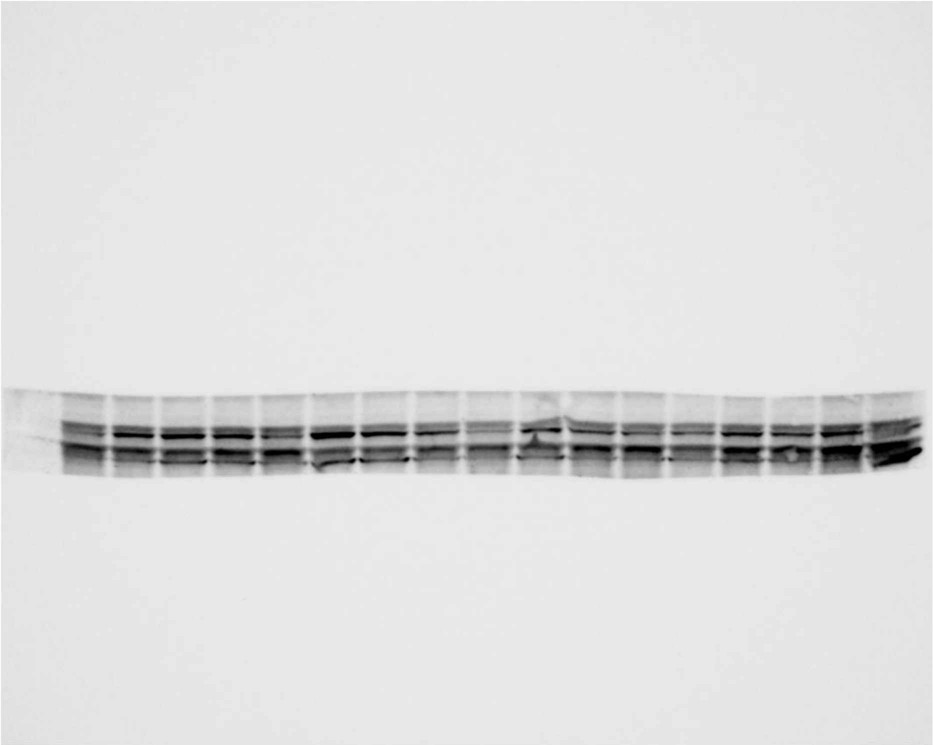

ERK1 & ERK2 females

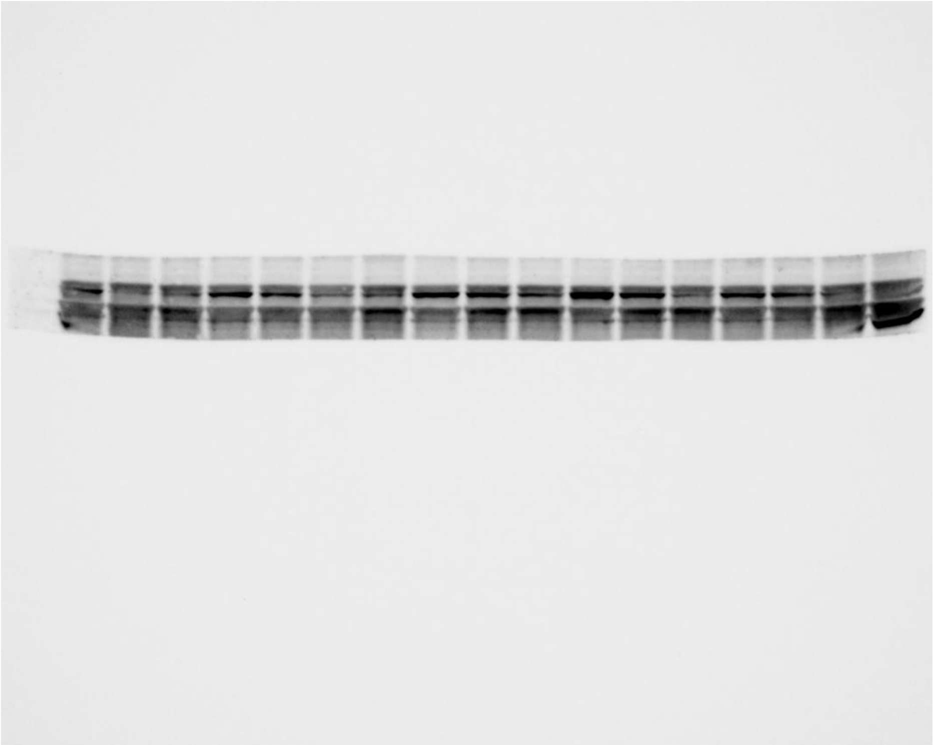

Figure S5. Unedited blots from Figure 7

γ-adaptin males

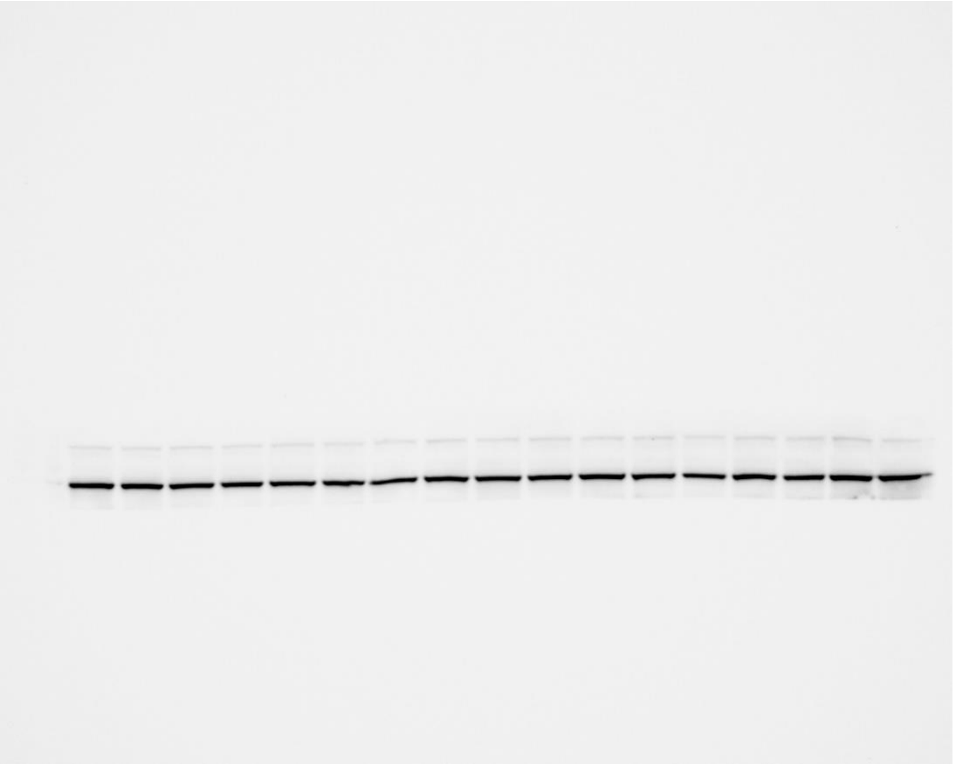

γ-adaptin females

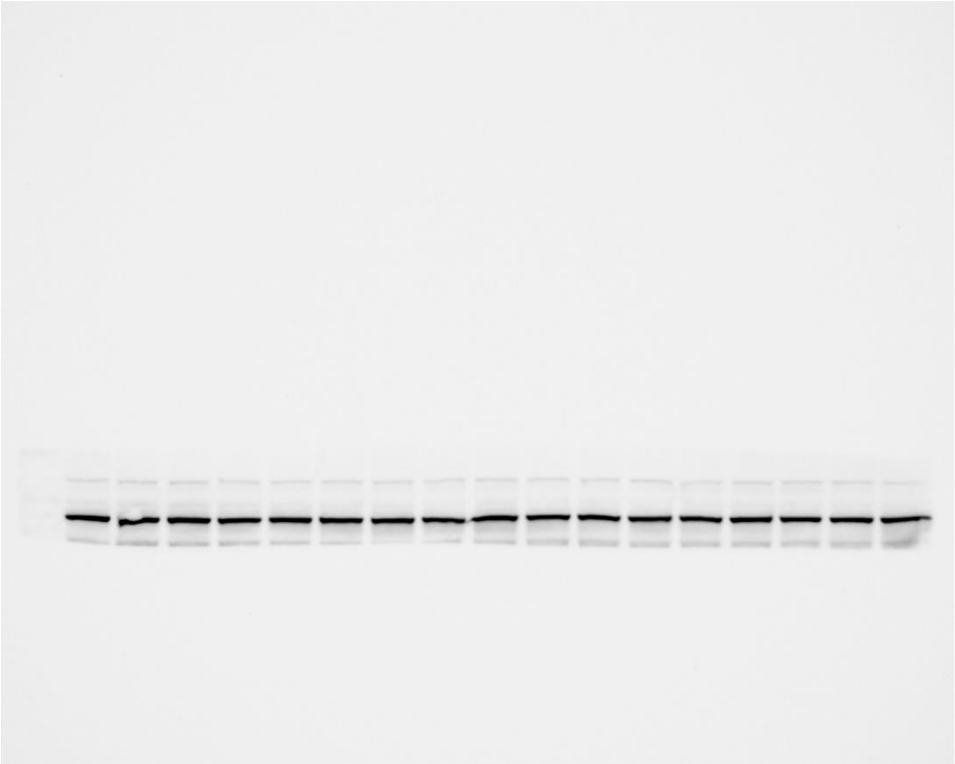

Ponceau staining males

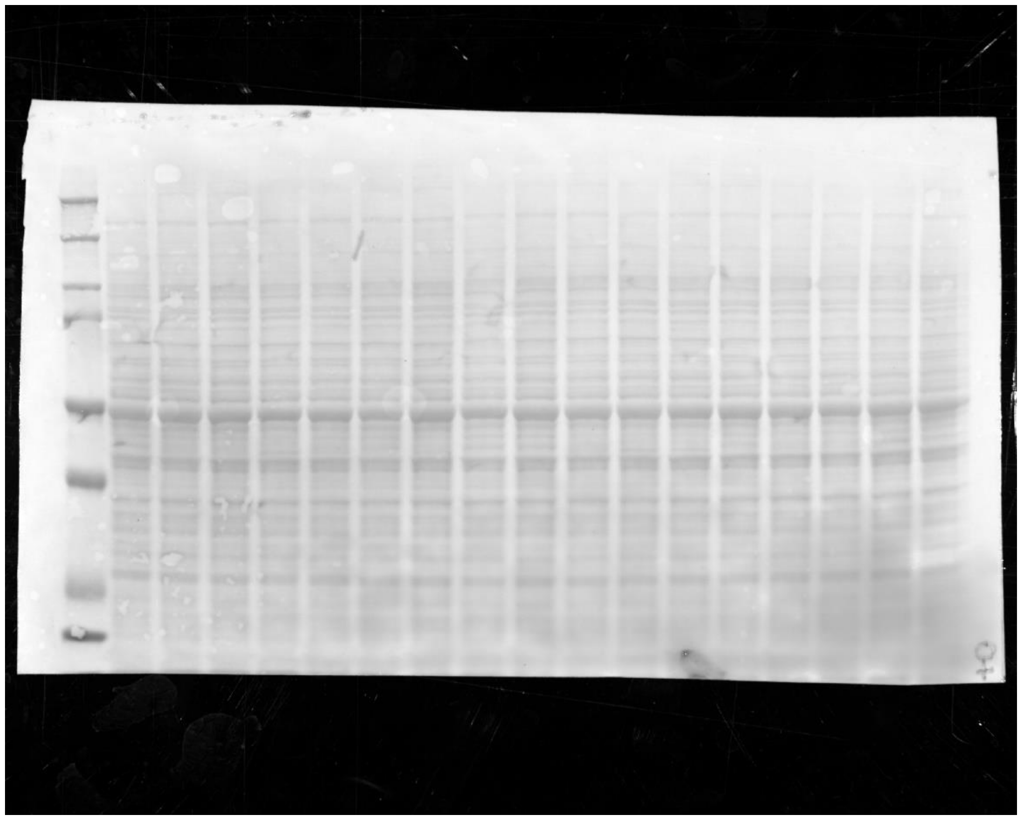

Ponceau staining females

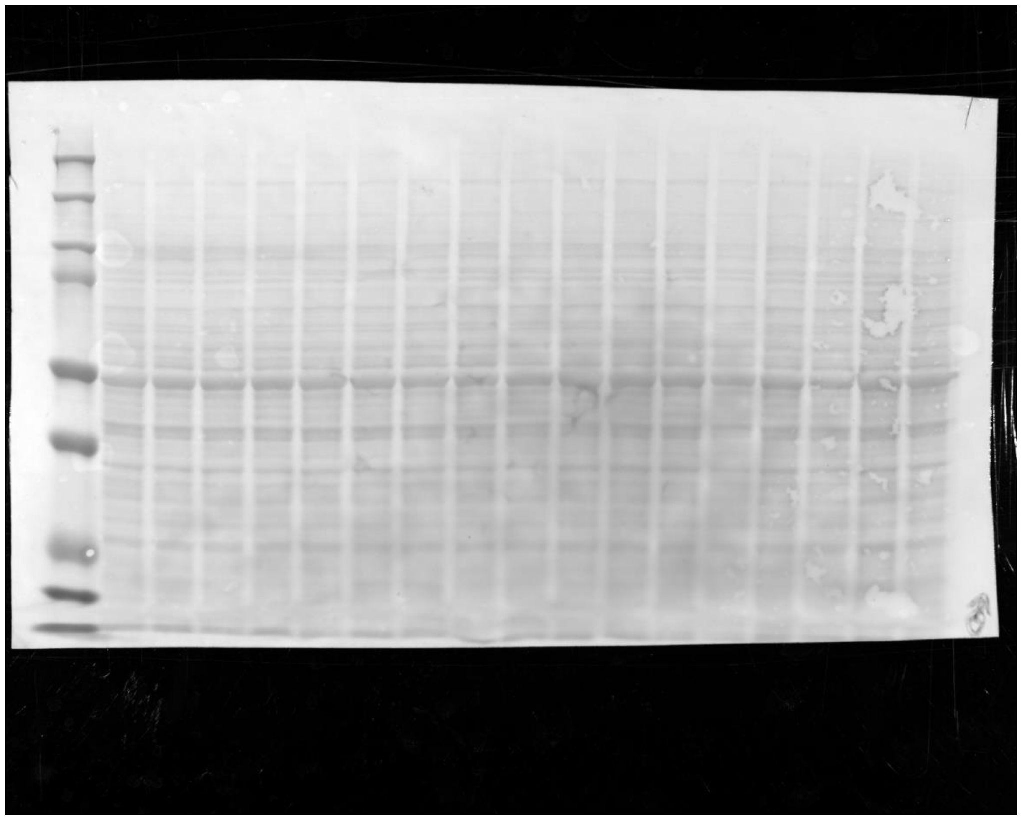

Figure S5. Unedited blots from Figure S3

UCP1 males

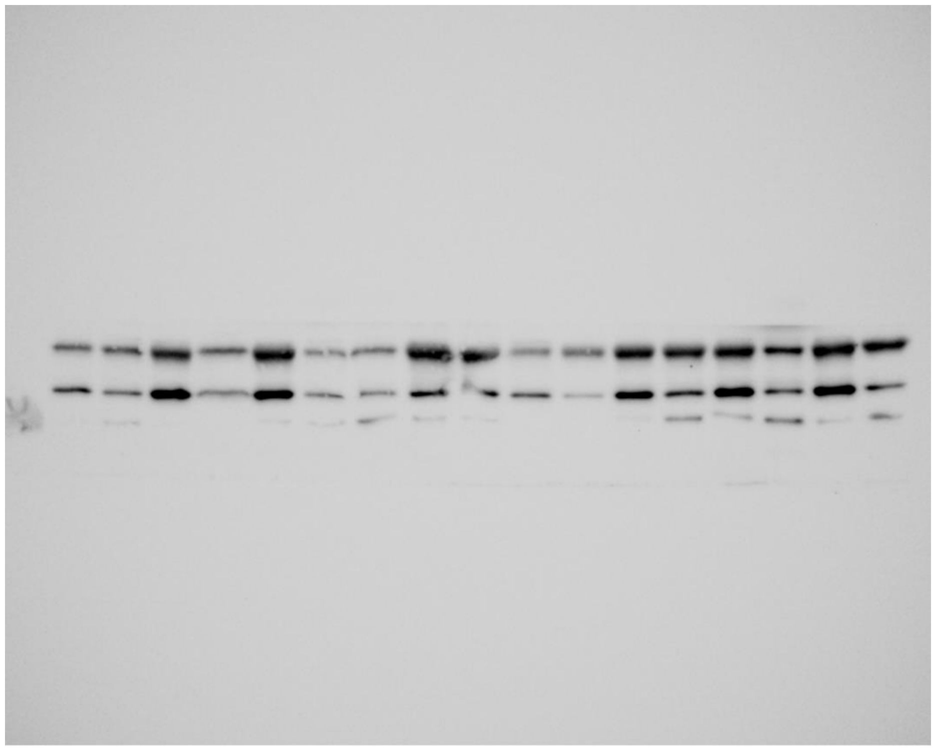

UCP1 females

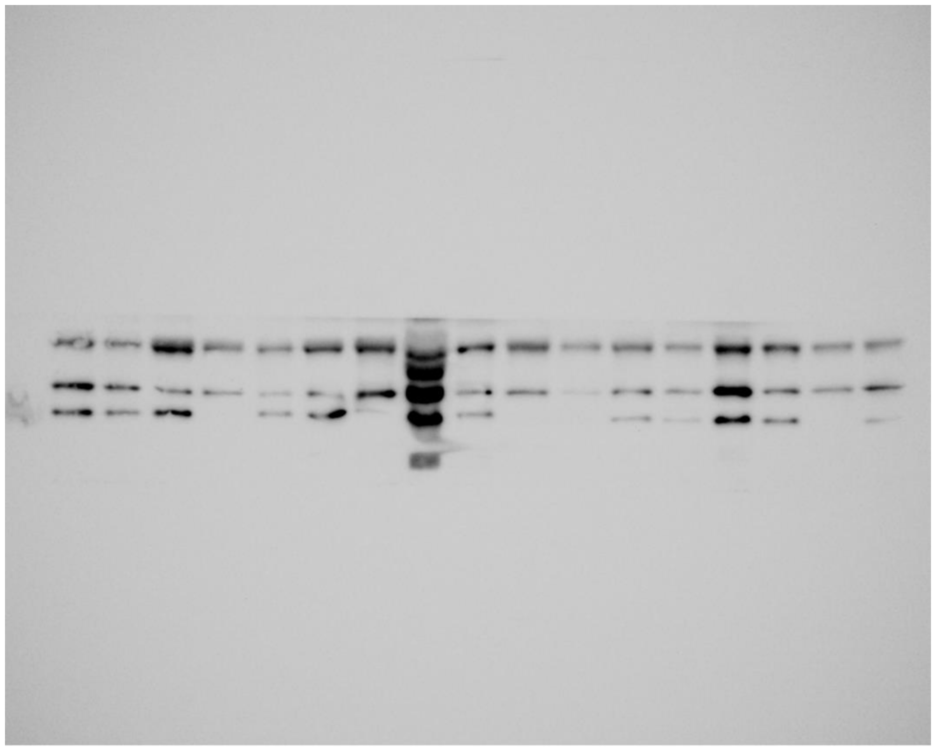

γ-adaptin males

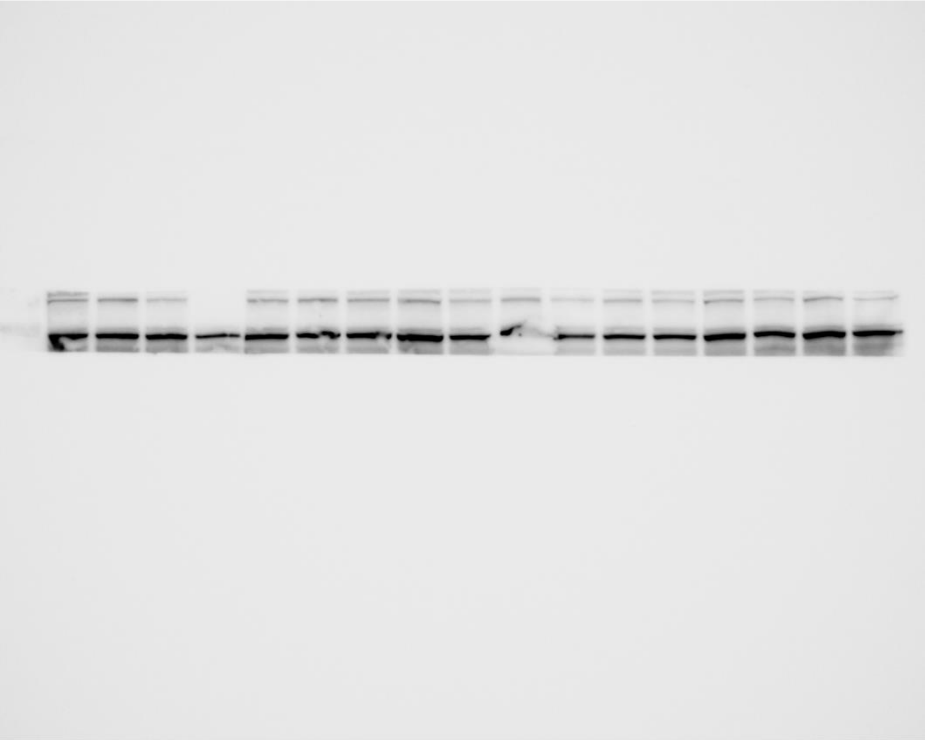

γ-adaptin females

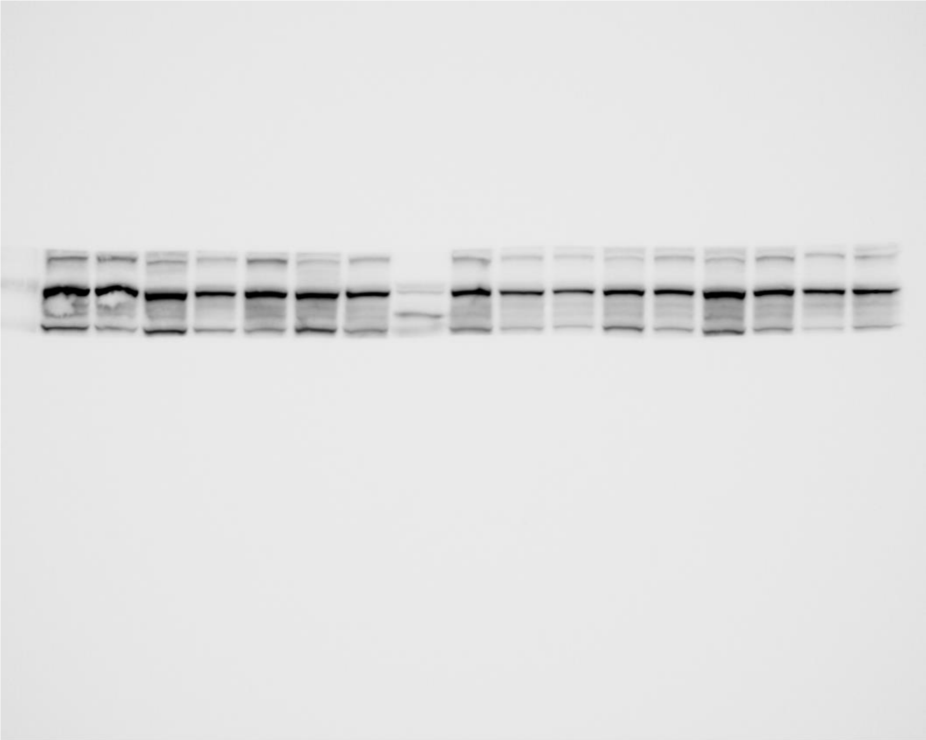

Figure S5. Unedited blots from Figure S3

Ponceau staining males

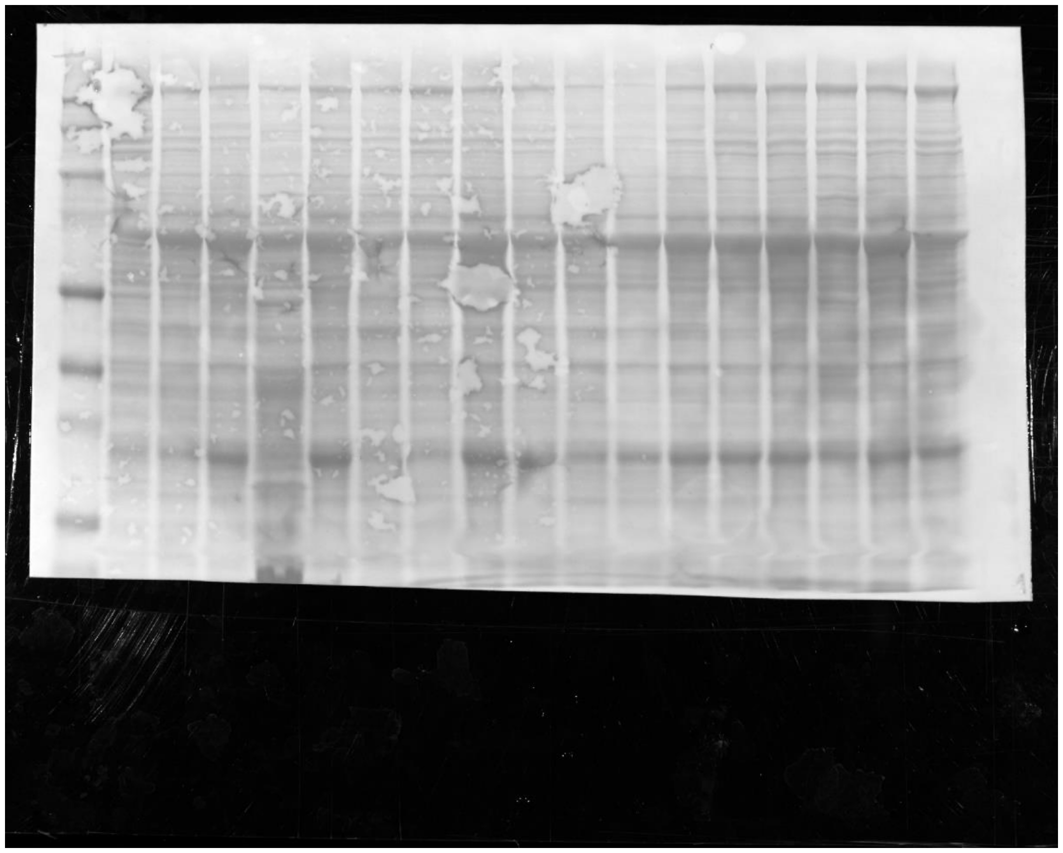

Ponceau staining females

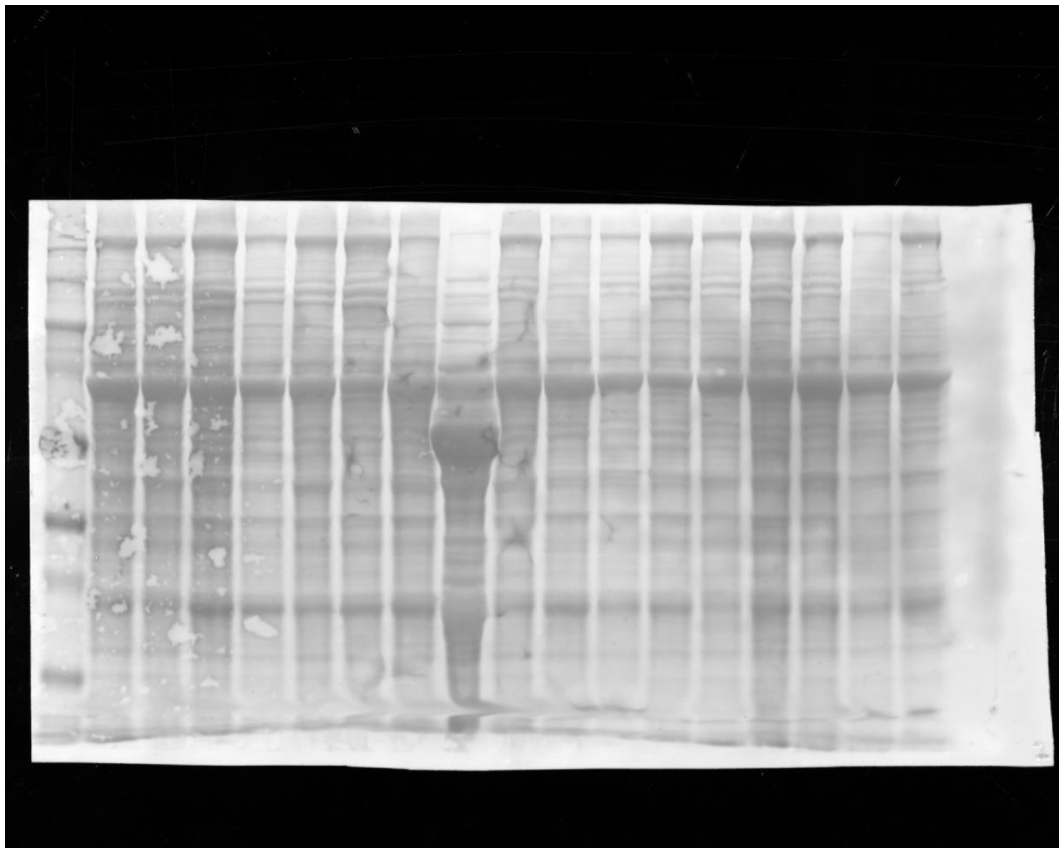

Figure S5. Unedited blots from Figure S4.

γ-adaptin males

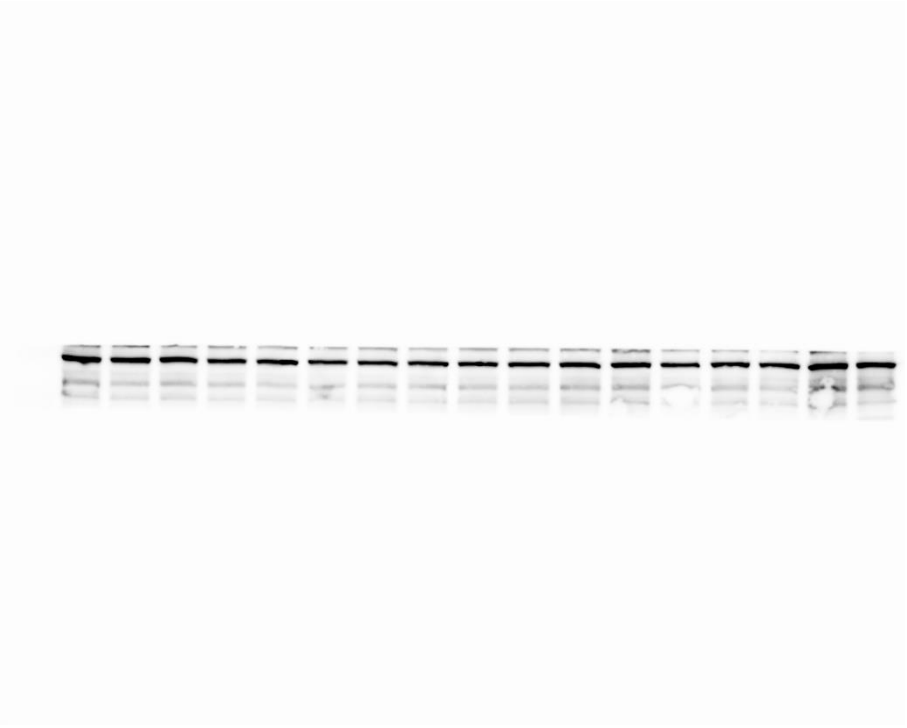

γ-adaptin females

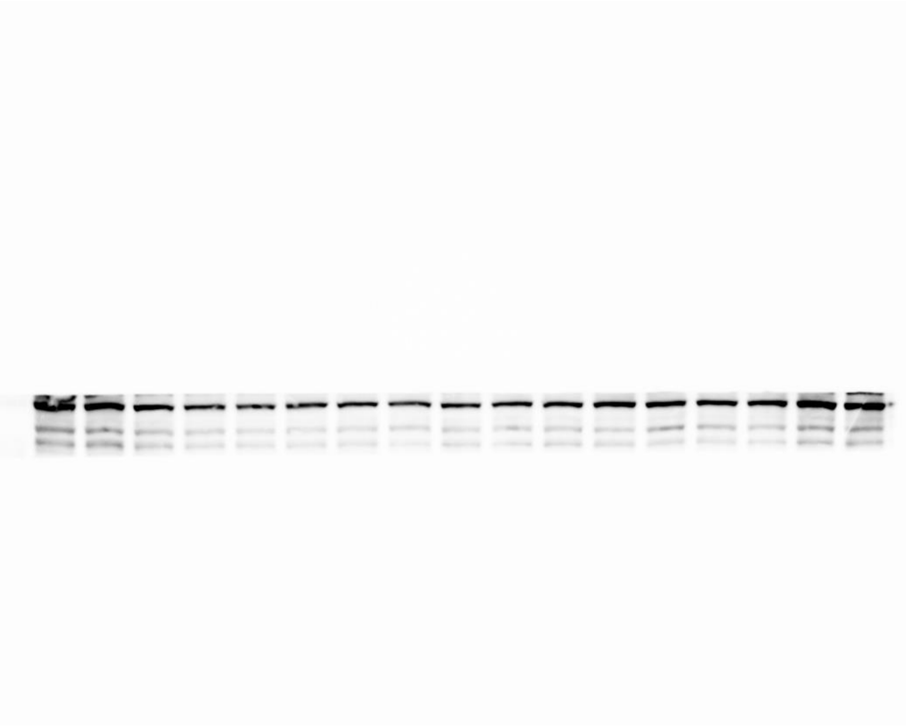

P(Ser9)-GSK3-β males

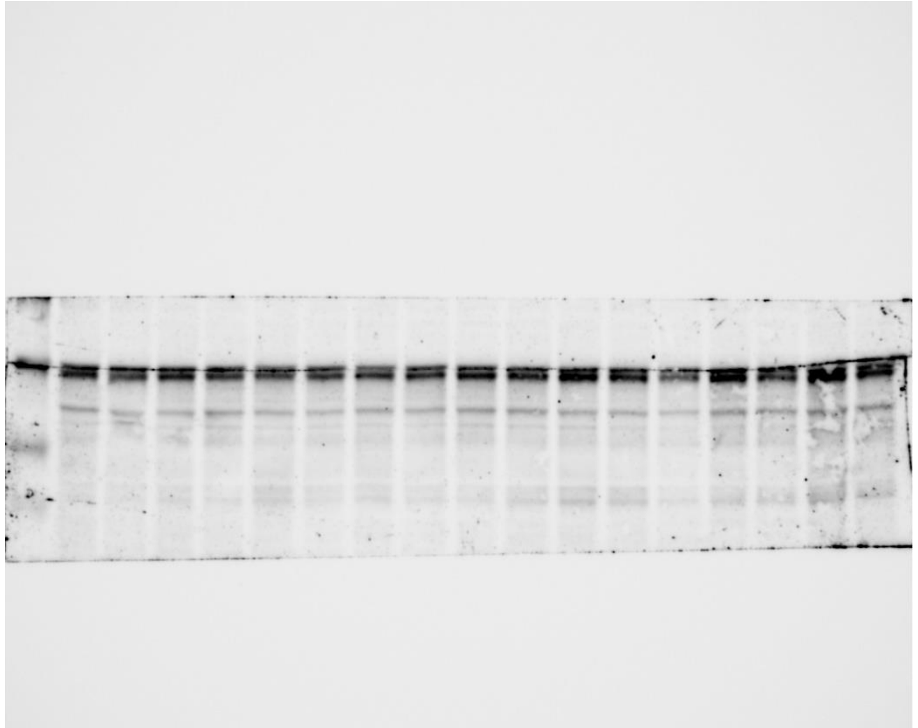

P(Ser9)-GSK3-β females

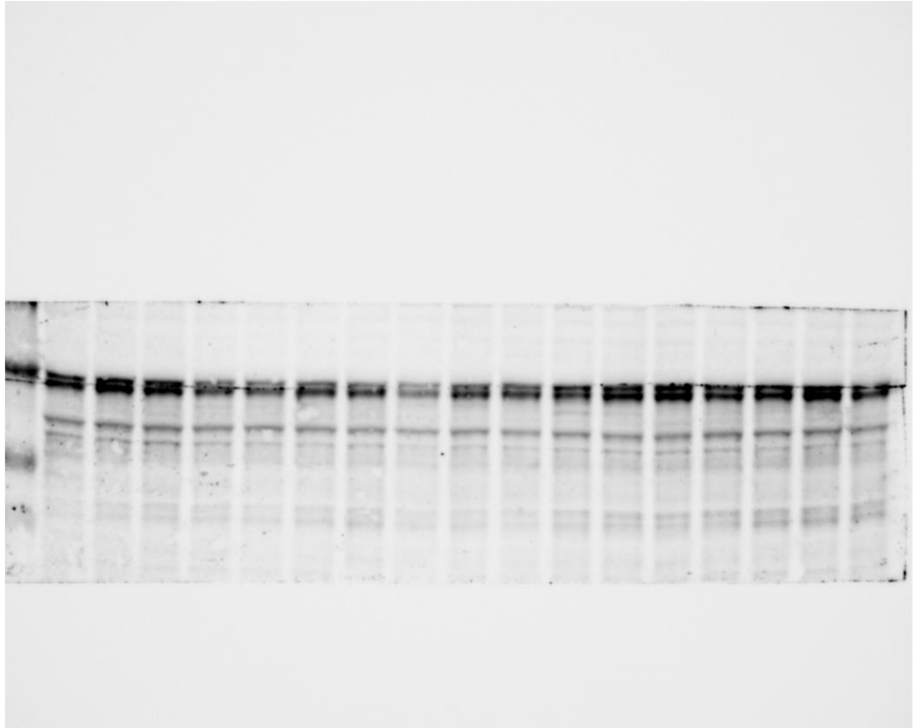

Figure S5. Unedited blots from Figure S4.

P(Tyr214/Tyr276)-GSK3-β males

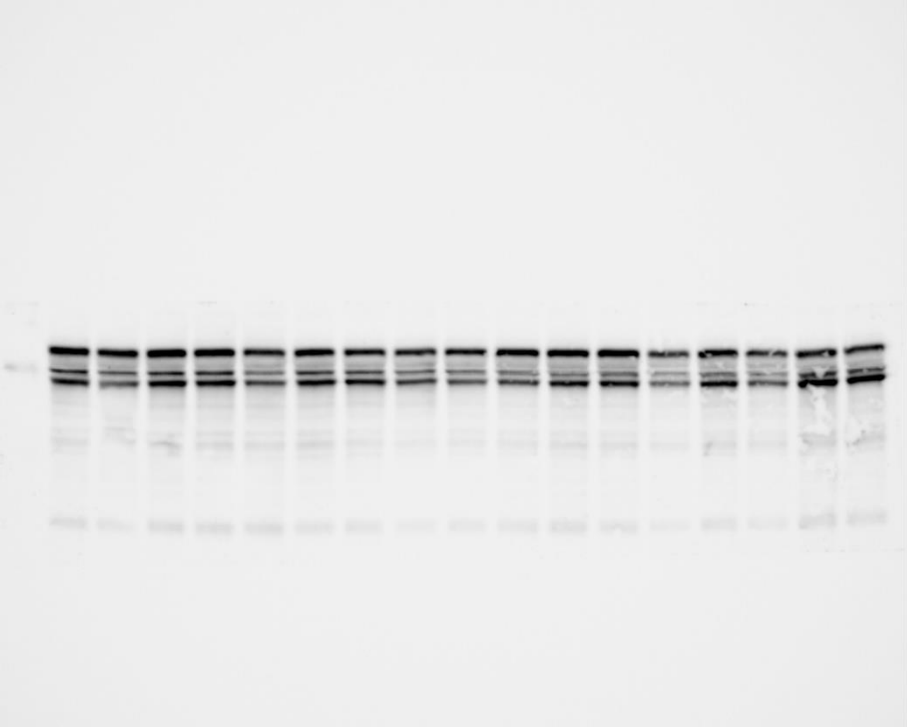

P(Tyr214/Tyr276)-GSK3-β females

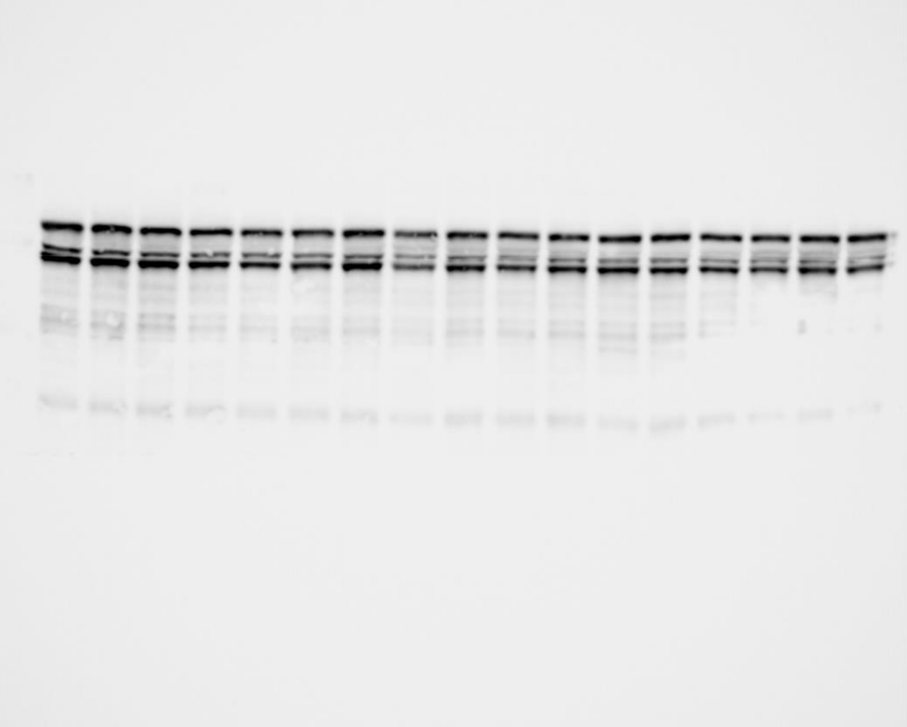

GSK3-β males

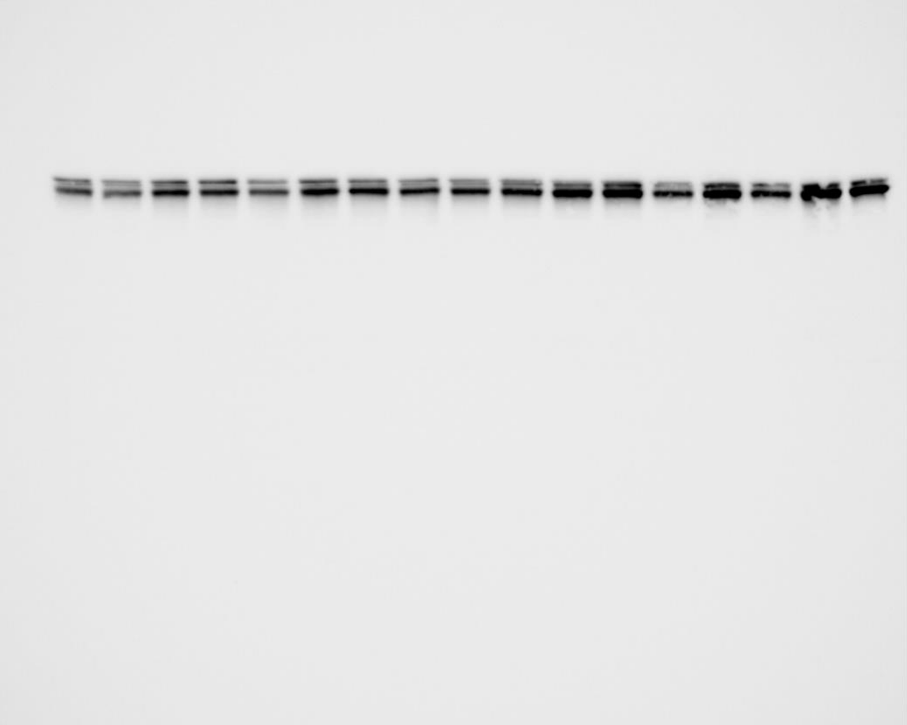

GSK3-β females

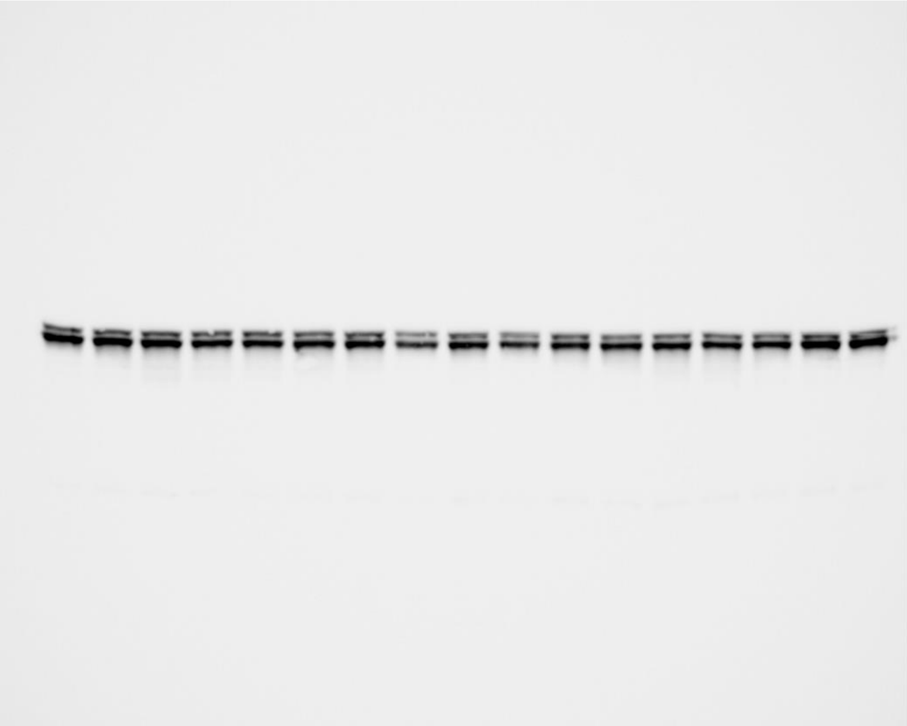

Figure S5. Unedited blots from Figure S4.

Ponceau staining males

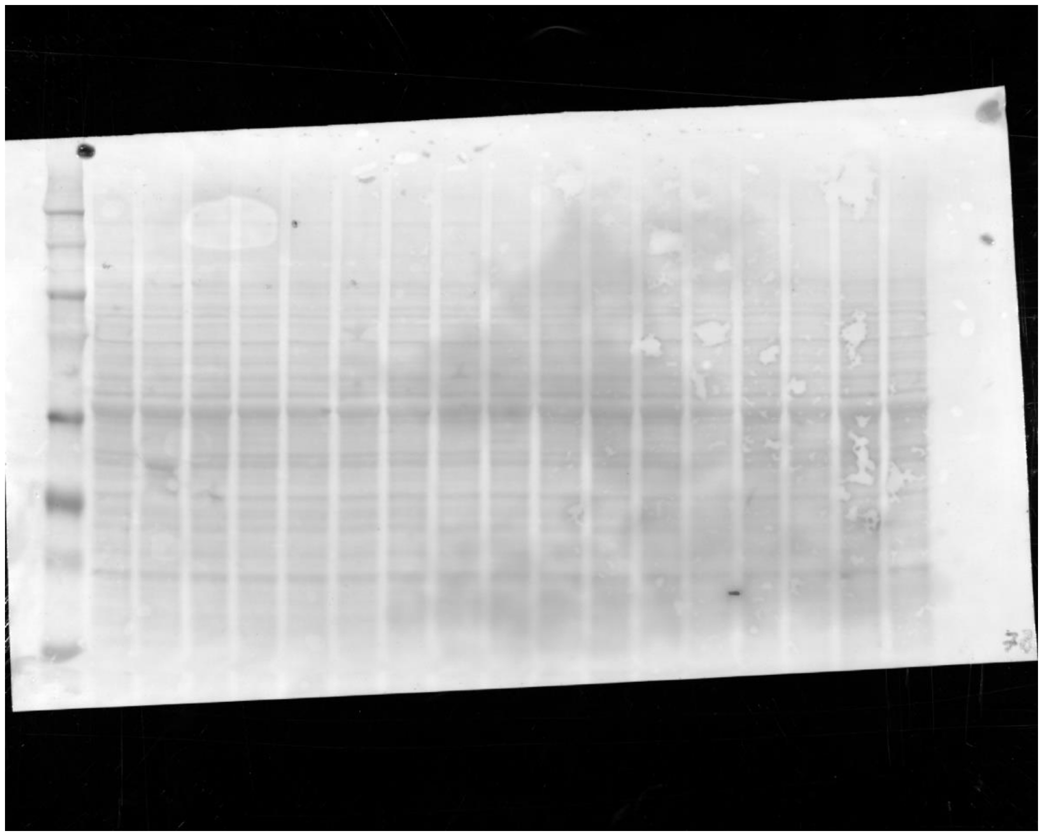

Ponceau staining females

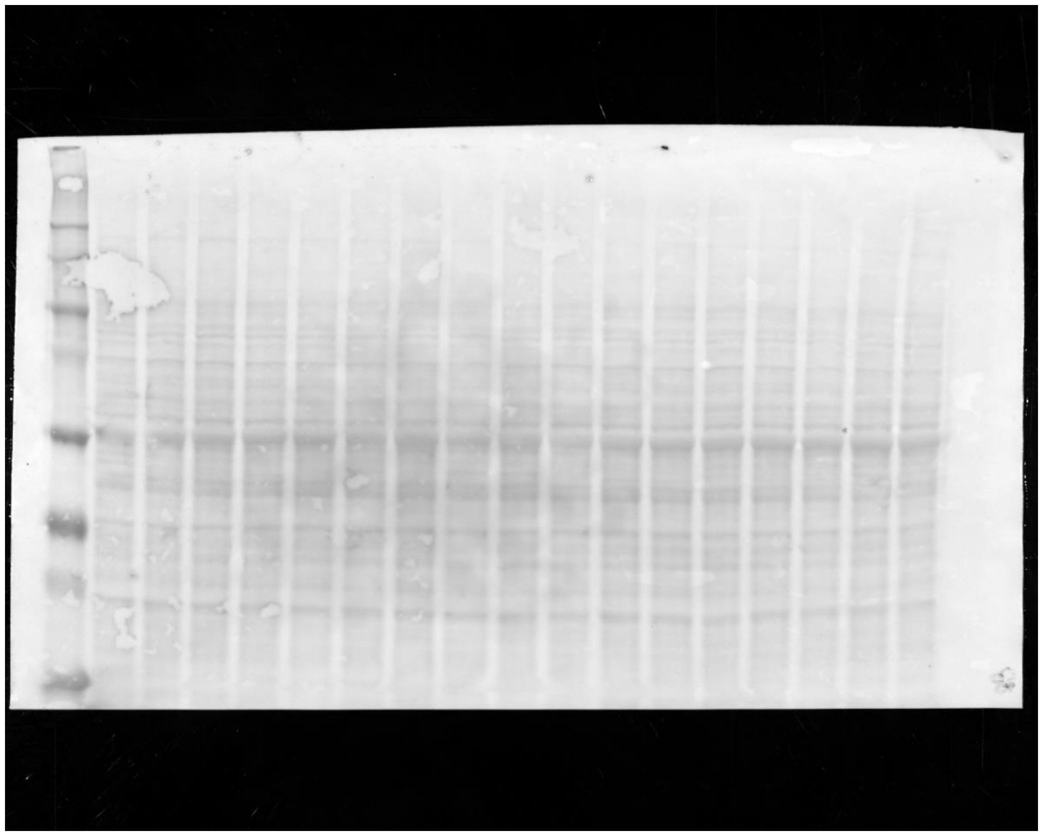

Supplement: Supplementary file 1 [file ijms-22-05365-s001.zip › Figure S5.pdf]
