# Supplementary material for: A Negative Energy Balance Is Associated with Metabolic Dysfunctions in the Hypothalamus of a Humanized Preclinical Model of Alzheimer’s Disease, the 5XFAD Mouse
Source: Int J Mol Sci. 2021 May 20;22(10):5365. doi: 10.3390/ijms22105365 (PMC8161294; doi:10.3390/ijms22105365)
Supplement: Supplementary file 1 [file ijms-22-05365-s001.zip › Tables S1 & S2.pdf]

Supplementary Tables

Table S1. Primer references for TaqMan® Gene Expression Assays (Applied Biosystems).

Gene ID	GenBank accession nº	Assay ID	Amplicon length
Actb	NM_007393.5	Mm02619580_g1	143
Agrp	NM_007427.3	Mm00475829_g1	86
Bdnf	NM_001048142.1	Mm04230607_s1	92
Blvra	NM_026678.4	Mm00466076_m1	73
Cartpt	NM_013732.7	Mm04210469_m1	102
Ghrl	NM_021488.5	Mm00612524_m1	104
Ghsr	NM_177330.4	Mm00616415_m1	63
Glp1r	NM_021332.2	Mm00445292_m1	70
Hcrt	NM_010410.2	Mm01964030_s1	168
Igf1r	NM_010513.2	Mm00802831_m1	106
Il1b	NM_008361.3	Mm00434228_m1	90
Il6	NM_031168.1	Mm00446190_m1	78
Insr	NM_010568.2	Mm01211879_m1	102
Lepr	NM_001122899.1	Mm00440181_m1	97
Npy	NM_023456.2	Mm01410146_m1	130
Pmch	NM_029971.2	Mm01242886_g1	78
Pomc	NM_001278581.1	Mm00435874_m1	60
Retn	NM_001204959.1	Mm00445641_m1	80
S100b	NM_009115.3	Mm00485897_m1	69
Tlr4	NM_021297.2	Mm00445273_m1	87
Tnf	NM_001278601.1	Mm00443258_m1	81

Table S2. Antibody references for Western Blot Assays.

Primary antibody	Specie	Manufacturing details	Dilution
AMPK	Rabbit	Cell Signaling (#5832)	1:1000
P(Ser202, Thr205)-Tau (AT8)	Mouse	ThermoFisher (#MN1020)	1:500
CDK5	Mouse	Cell Signaling (#12134)	1:1000
ERK1/2	Rabbit	Cell Signaling (#9102)	1:1000
GFAP	Mouse	Sigma-Aldrich (G3893)	1:500
GHSR	Rabbit	ThermoFisher (PA5-28752)	1:1000
GLP-1R	Rabbit	Abcam (ab39072)	1:500
P(Ser9)-GSK3-β	Rabbit	Cell Signaling (#9322)	1:1000
P(Tyr279/Tyr216)-GSK3	Mouse	Sigma-Aldrich (05-413)	1:1000
GSK3-β	Rabbit	Cell Signaling (#12456S)	1:1000
Iba1	Rabbit	Abcam (ab5076)	1:1000
Il-1β	Mouse	Cell Signaling (#12242)	1:1000
IR	Mouse	Cell Signaling (#3020)	1:1000
IRS1	Rabbit	Cell Signaling (#3407P)	1:1000
LepR [EPR911(N)]	Rabbit	Abcam (ab177469)	1:500
LRP1	Rabbit	Cell Signaling (#64099)	1:1000
mTOR	Rabbit	Cell Signaling (#2972)	1:1000
P(ser612)-IRS1	Rabbit	Cell Signaling (#3203P)	1:1000
P(Tyr892)-IRS1	Rabbit	Abcam (ab46800)	1:1000
P35/P25	Rabbit	Cell Signaling (#2680)	1:1000
P(Thr172)-AMPK	Rabbit	Cell Signaling (#2535)	1:1000
P(Thr202/Tyr204)-ERK1/2	Rabbit	Cell Signaling (#9101)	1:1000
P(Tyr1158, Tyr1162, Tyr1163)-IR	Rabbit	Abcam (ab5681)	1:1000
P(Tyr1077)-LepR	Rabbit	Sigma-Aldrich (07-1317)	1:1000
P(Ser2448)-mTOR	Rabbit	Cell Signaling (#2971)	1:1000
P(Tyr703)-STAT3	Rabbit	Cell Signaling (#91455)	1:1000
P(Tyr694)-STAT5	Rabbit	Cell Signaling (#9351)	1:500
STAT3	Mouse	Cell Signaling (#91395)	1:1000
STAT5	Rabbit	Cell Signaling (#9363)	1:1000
Tau	Rabbit	Abcam (ab203179)	1:500
TLR4	Rabbit	Abcam (ab217274)	1:1000
UCP1	Rabbit	Abcam (ab10983)	1:1000
γ-adaptin	Mouse	BD Biosciences (610385)	1:2000
